# Facile Synthesis of Saikosaponins

**DOI:** 10.3390/molecules26071941

**Published:** 2021-03-30

**Authors:** Ziqiang Wang, Bingcheng Wei, Tong Mu, Peng Xu, Biao Yu

**Affiliations:** 1 Department of Chemistry, University of Science and Technology of China, 96 Jinzhai Road, Hefei 230026, China; 18355122023@163.com; 2 State Key Laboratory of Bio-organic and Natural Products Chemistry, Center for Excellence in Molecular Synthesis, Shanghai Institute of Organic Chemistry, Chinese Academy of Sciences, 345 Lingling Road, Shanghai 200032, China; weibc@sioc.ac.cn (B.W.); mutong@sioc.ac.cn (T.M.); 3 School of Chemistry and Materials Science, Hangzhou Institute for Advanced Study, University of Chinese Academy of Sciences, 1 Sub-lane Xiangshan, Hangzhou 310024, China

**Keywords:** saikosaponin, triterpene, glycoside, glycosylation, gold(I)

## Abstract

Saikosaponin A (SSa) and D (SSd) are typical oleanane-type saponins featuring a unique 13,28-epoxy-ether moiety at D ring of the aglycones, which exhibit a wide range of biological and pharmacological activities. Herein, we report the first synthesis of saikosaponin A/D (**1**–**2**) and their natural congeners, including prosaikosaponin F (**3**), G (**4**), saikosaponin Y (**5**), prosaikogenin (**6**), and clinoposaponin I (**7**). The present synthesis features ready preparation of the aglycones of high oxidation state from oleanolic acid, regioselective glycosylation to construct the β-(1→3)-linked disaccharide fragment, and efficient gold(I)-catalyzed glycosylation to install the glycans on to the aglycones.

## 1. Introduction

**Figure 1:**
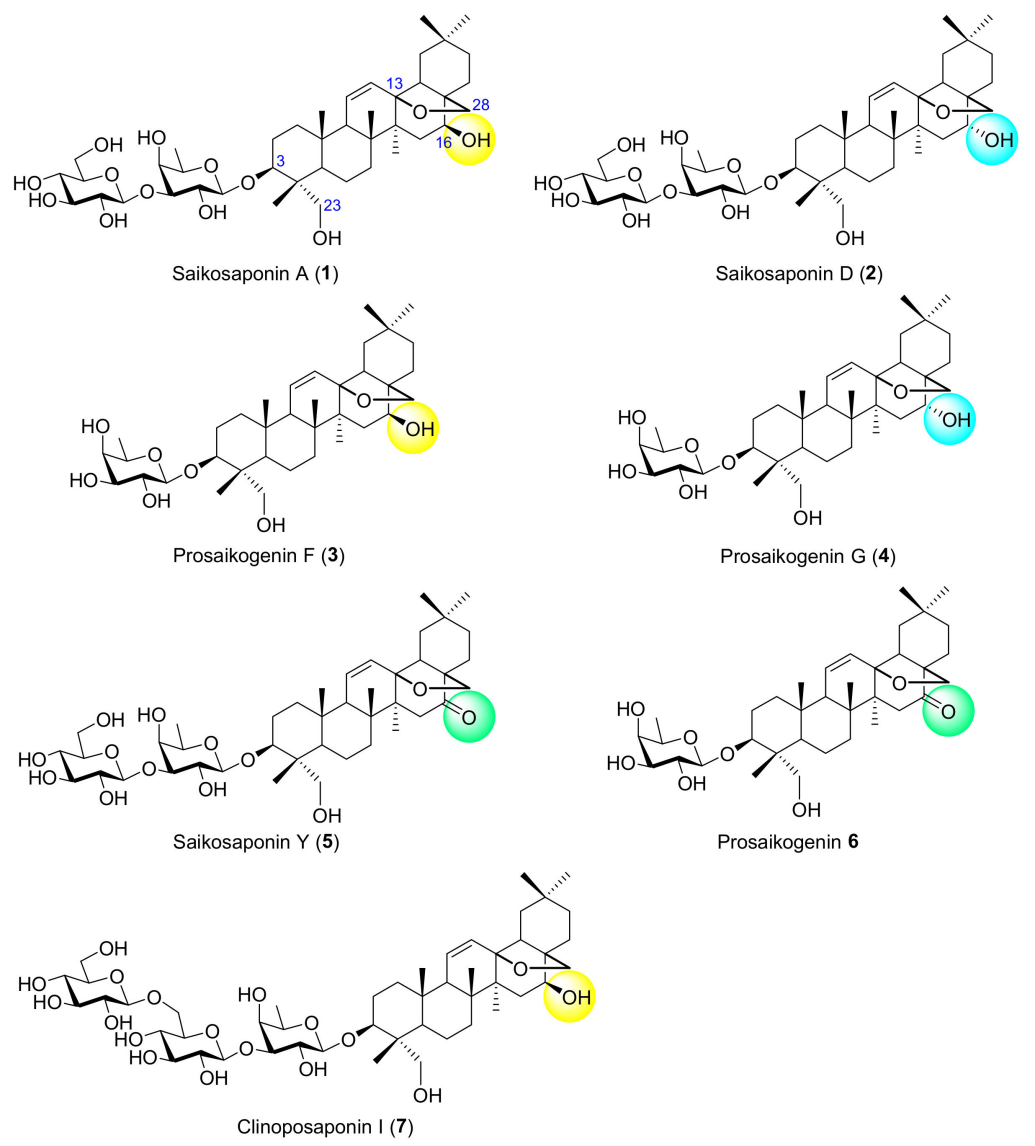
Saikosaponin A (**1**), D (**2**), and congeners (**3**–**7**).

The roots of *Radix Bupleuri*, one of the most common traditional Chinese medicines, have been frequently used for the treatment of common cold with fever, influenza, inflammation, and infectious diseases [1,2]. Saikosaponin A (SSa, **1**) and D (SSd, **2**) are the major compounds in *Radix Bupleuri* and are used as the markers for evaluation of the quality of the medicine (Figure 1) [3]. Indeed, these two triterpene glycosides together with their congeners exhibit a variety of pharmacological activities, including anti-inflammatory, anti-tumor, neuro-regulation, immuno-regulation, hepatoprotection, and antiviral effects [1,4]. Structurally, SSa (**1**) and SSd (**2**) are a pair of the oleanane-type disaccharide stereoisomers, with the aglycones, namely saikogenin F (SGF) and saikogenin G (SGG), respectively, featuring a 13,28-epoxy-ether moiety at the D ring and an opposite chirality at 16-OH. Interestingly, SSa (**1**) was reported to show a strong anti-inflammatory effect, whereas SSd (**2**) showed a strong antitumor effect [1]. The corresponding monosaccharides, named prosaikogenin F (PSF, **3**) and G (PSG, **4**) were identified as metabolites of SSa and SSd [5], which showed hemolytic activity and the absorbability on red blood cells [6,7]. Saikosaponin Y (**5**) is likely a biogenetic precursor/product of SSa and SSd, which has a higher oxidation state at C-16; this congener displayed potent cytotoxic and antiviral activities [8,9,10]. Monosaccharide **6**, named posaikogenin, was found as an artifact which might be derived from disaccharide **5**. A number of saikosaponin-like compounds have also been identified from *Clinopodium gracile* and other plants, among which Clinoposaponin I (**7**) bearing a trisaccharide residue is a typical one [11,12,13]. All these seven saponins (**1**–**7**) belong to type I saikosaponins, which bear the 13,28-epoxy-ether moiety, and this epoxy-ether moiety is found crucial to their cytotoxic activities.

Notwithstanding, these naturally extracted saikosaponins are associated with systemic toxicities and side effects [14,15]. Thus, saikosaponins could induce obvious hepatoxicity and necrosis upon 15 day administration to rats [14]. Furthermore, SSd (**2**) alone was found to stimulate mitochondrial apoptosis in human hepatocyte cells [15]. On the other hand, these saikosaponins are difficult to purify from plants; therefore, in-depth studies have not been carried out on their structure-activity relationship (SAR) and mode of action. Chemical synthesis of saikosaponins is also a challenging task, given the scarce availability of the triterpene aglycones that bear high oxidation state at the D/E rings [16,17,18,19]. Very recently, we disclosed a site-selective C-H hydroxylation reaction at the D/E rings of pentacyclic triterpenoids, thus paving a venue for the synthesis of saikosaponins [18,20]. Herein, we report the synthesis of saikosaponin A (**1**), D (**2**), and their congeners (**3**–**7**), using the largely available oleanolic acid as a starting material.

## 2. Results

**Scheme 1:**
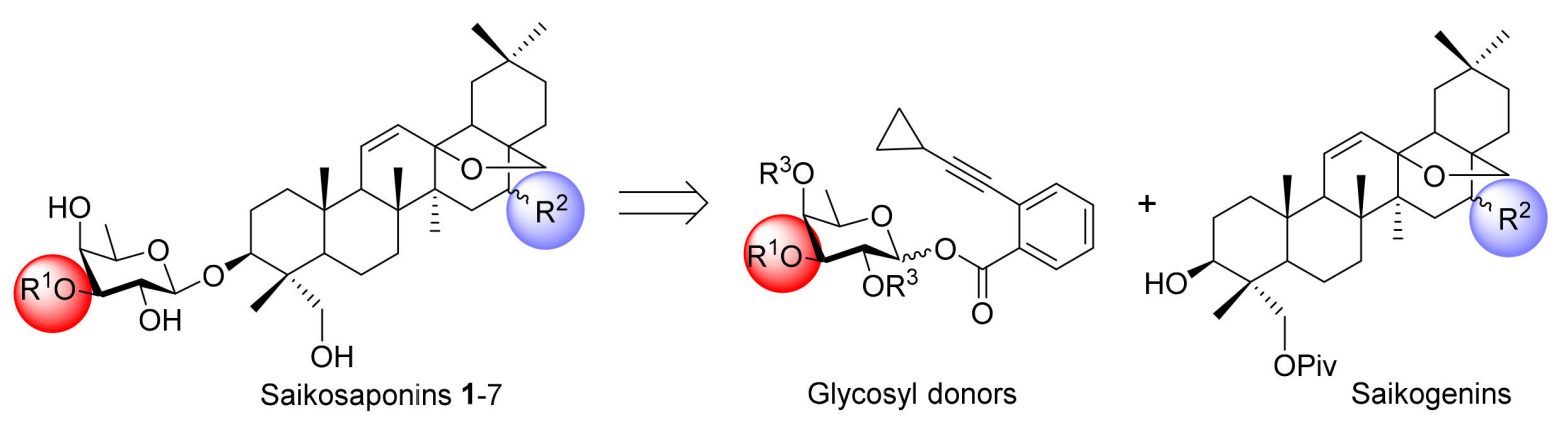
Retrosynthetic plan for saikosaponins **1**–**7**.

We envisioned the assembly of saikosaponins (**1**–**7**) from two pieces, i.e., a saikogenin derivative and a glycosyl donor (Scheme 1) [21]. Considering the susceptibility of the 13,28-epoxy-ether moiety in the aglycones, we planned to employ glycosyl *o*-alkynylbenzoates as donors that can proceed glycosylation under the mild gold(I)-catalyzed conditions.

**Scheme 2:**
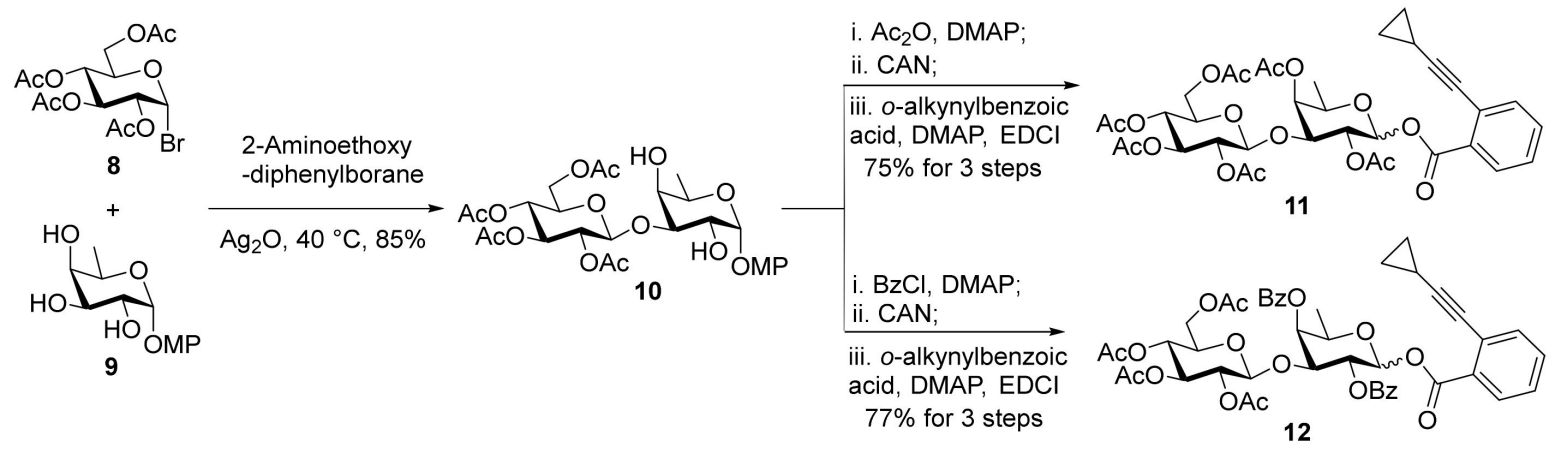
Preparation of disaccharide *o*-alkynylbenzoates **11** and **12**.

The requisite disaccharide *o*-alkynylbenzoates **11** and **12** were readily prepared via a regioselective glycosylation approach (Scheme 2) [22]. In view of the higher reactivity of the equatorial 3-OH versus the equatorial 2-OH and the axial 4-OH in *p*-methoxyphenyl β-d-fucopyranoside **9**, condensation of triol **9** with glucosyl bromide **8** under the modified Taylor’s conditions led to the desired (1→3)-disaccharide **10** smoothly (85%) [23,24]. Acetylation of diol **10**, oxidative cleavage of the anomeric MP group with CAN, and esterification with *o*-alkynylbenzoic acid furnished disaccharide *o*-alkynylbenzoate **11** (75% for 3 steps) [25,26]. Since the 2-*O*-acetyl group might result in orthoester formation or migration to the aglycone during the glycosylation reactions [27,28,29], 2,4-di*-O*-benzoyl *o*-alkynylbenzoate **12** was also prepared (77% for 3 steps).

**Scheme 3:**
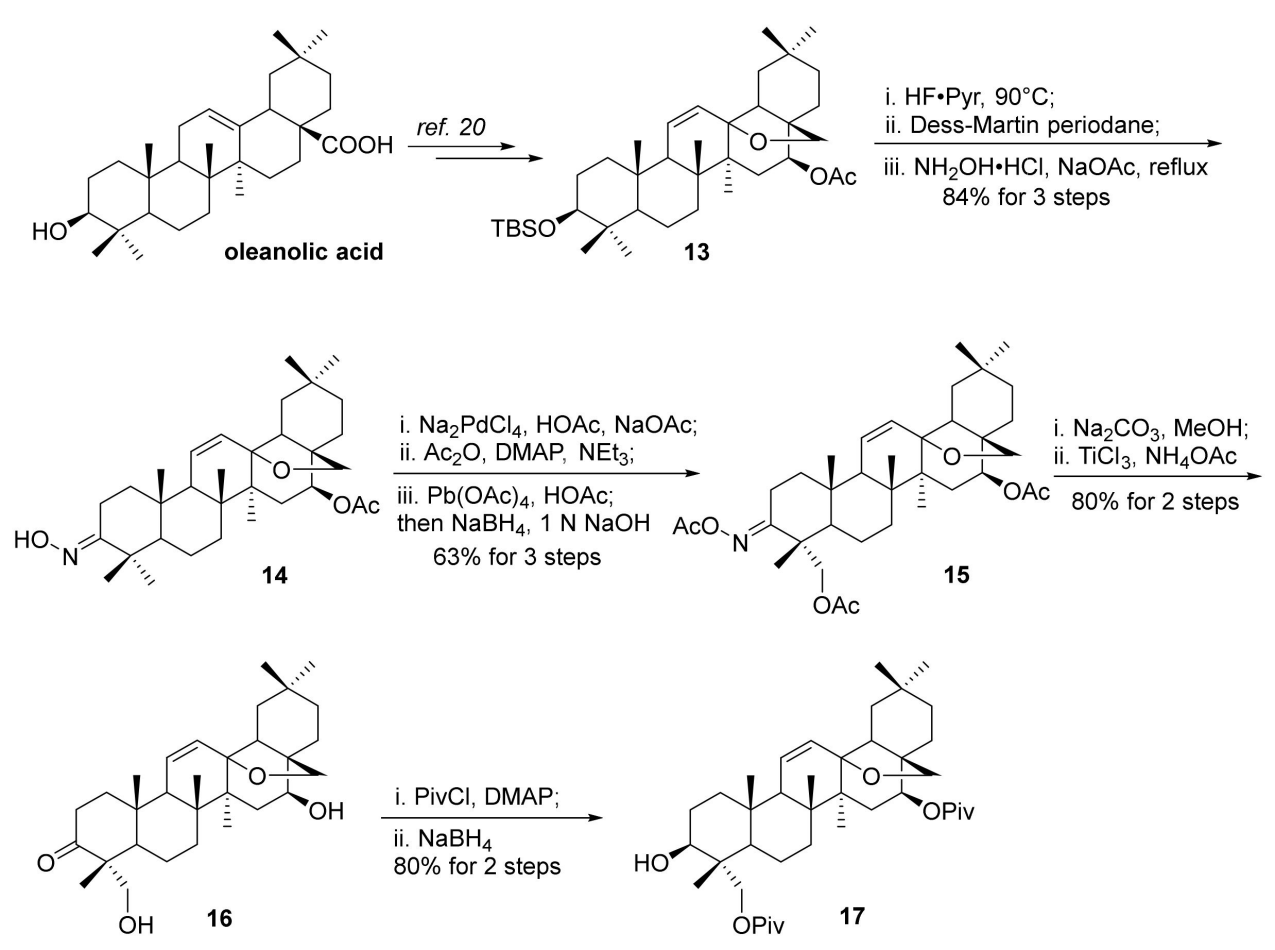
Preparation of aglycone derivative **17**.

The synthesis of saikogenins commenced with known compound **13**, which was readily prepared from oleanolic acid in 7 steps (Scheme 3) [20]. Desilylation with the Olah reagent, oxidation of the resultant 3-OH, and subsequent oxime formation with hydroxylamine hydrochloride afforded oxime **14** (84%). It was noted that desilylation with TBAF under reflux conditions led to unmask of the 16-*O*-acetyl group. Selective hydroxylation of the C-4 equatorial methyl group was then achieved following Baldwin’s method to furnish **15** (63%) [30,31,32,33,34]. that involved (1) cyclopalladation of the equatorial methyl group from oxime **14** with Na_2_PdCl_4_, (2) acetylation of the oxime hydroxyl group, and (3) oxidation with Pb(OAc)_4_ followed by reductive workup with NaBH_4_. Hydrolysis of **15** under basic conditions and then with TiCl_3_/NH_4_OAc gave 3-one-16-β-ol **16**. The nascent 16-β-OH and 23-OH were firstly protected with acetyl groups; however, subsequent reduction of the 3-ketone with NaBH_4_ led to a mixture, due to acetyl group migration under basic conditions [35,36]. Thus, diol **16** was masked with the bulkier pivaloyl groups and then the carbonyl was reduced with NaBH_4_ to afford the desired aglycone derivative **17** (80%) [37].

**Scheme 4:**
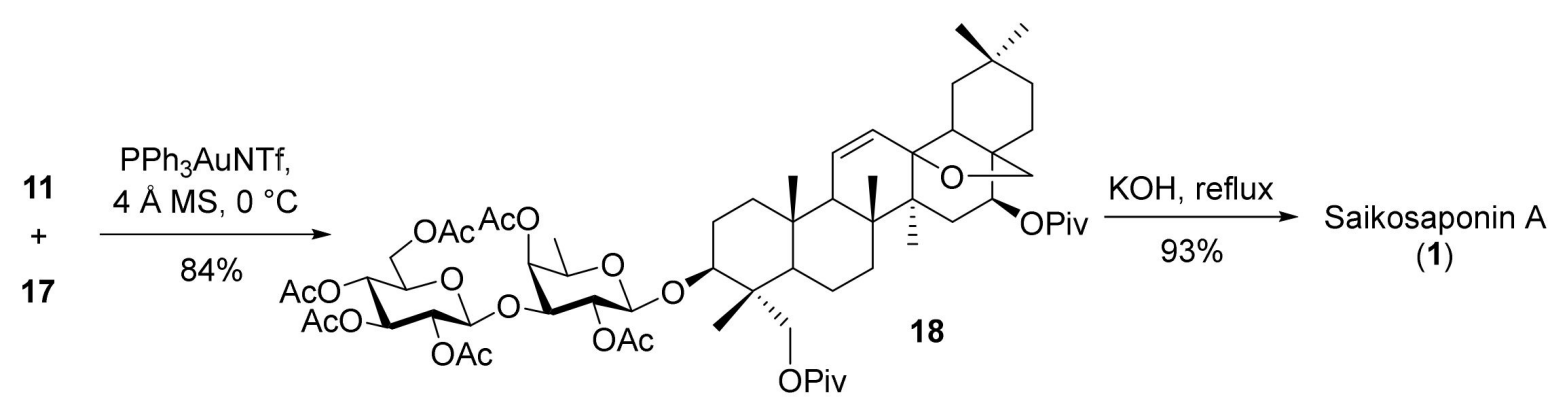
Gold(I)-catalyzed glycosylation and synthesis of saikosaponin A (**1**).

As expected, condensation of **17** with disaccharide *o*-alkynylbenzoate **11** proceeded smoothly under the catalysis of PPh_3_AuNTf_2_ (0.1 equiv.) [38], leading to glycoside **18** (84%) (Scheme 4). Alternatively, coupling of **17** with a relevant disaccharide trichloroacetimidate under the promotion of TMSOTf (0.1 equiv.) failed to provide glycoside **18**, due to decomposition of **17** under the acidic conditions [5,39]. Finally, saponification of **18** under basic and reflux conditions furnished SSa (**1**) in 93% yield [40].

**Scheme 5:**
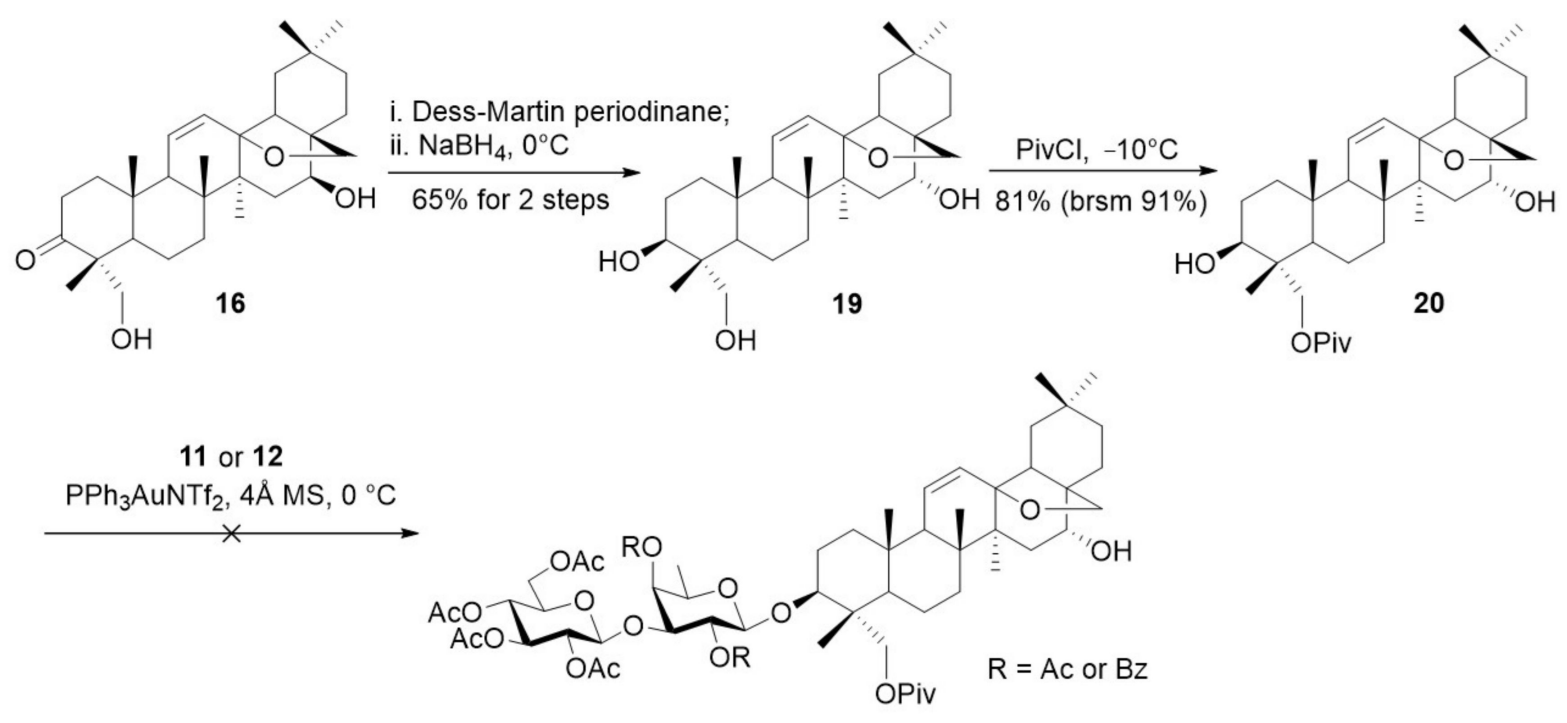
An attempt at glycosylation of diol **20**.

SSd (**2**) could be synthesized by modification of the synthetic approach to SSa (**1**) (Scheme 5). Thus, the configuration of 16-β-OH in **16** was inverted via Dess-Martin oxidation and NaBH_4_ reduction, giving SGG (**19**) in good yield (65%). Selective protection of the primary 23-OH with pivaloyl group provide diol **20** (81%). It was reported that regioselective acetylation of the triterpene C3-OH in the presence of C16-OH was feasible [41]. Thus, we attempted a regioselective glycosylation of 3,16-diol **20**. Disappointingly, the glycosylation reactions with disaccharide donors **11** and **12** were found to futile, leading to complex mixtures.

**Scheme 6:**
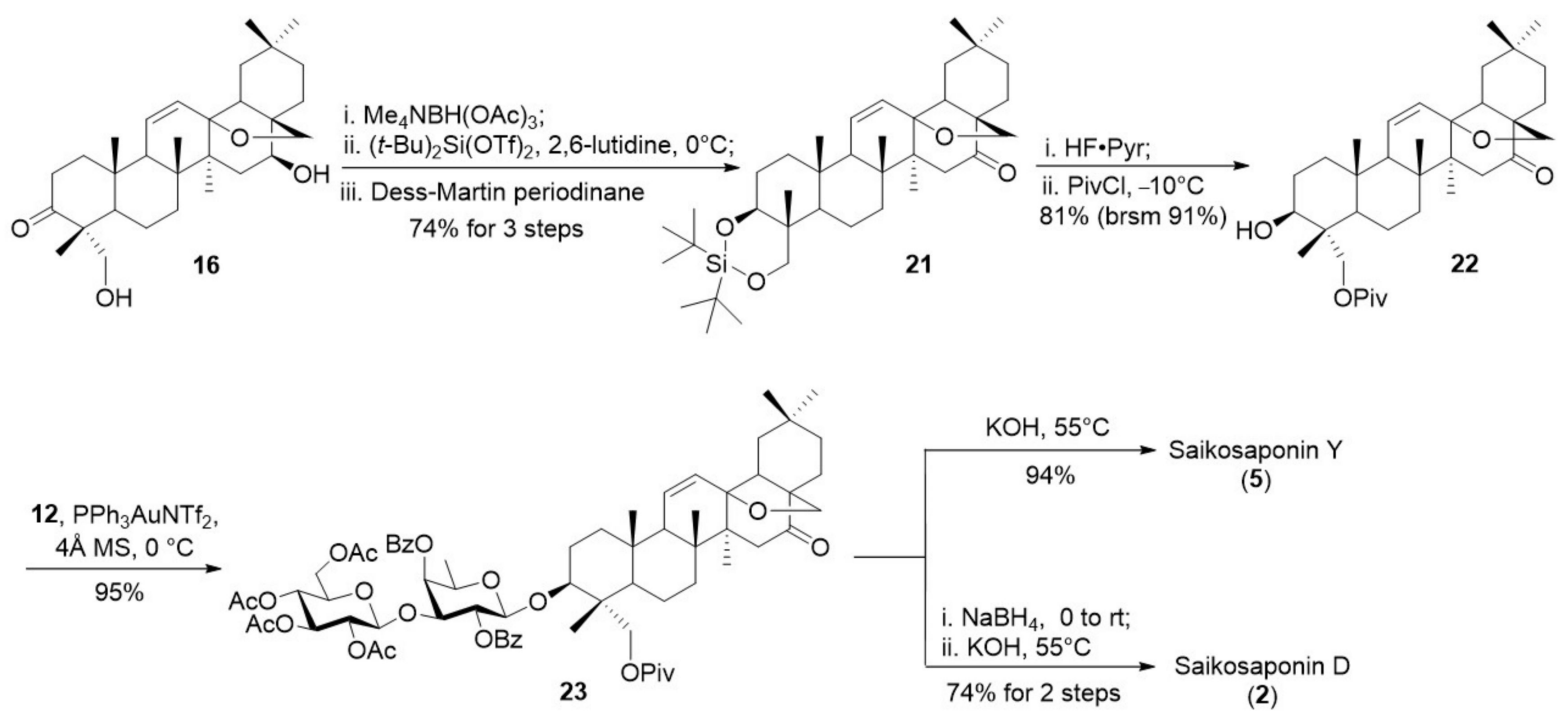
Synthesis of saikosaponin D (**2**) and saikosaponin Y (**5**).

Considering the influence of the 16-α-OH on the 3-*O*-glycosylation, we arranged the reduction of 16-ketone at a late stage (Scheme 6). Thus, the 3-ketone in **16** was reduced with Me_4_NBH(OAc)_3_ [42,43], the resultant 3,23-diol was then protected with silylidene ketal, subsequent Dess-Martin oxidation of the remaining 16-OH led to ketone **21** (74% for 3 steps). Next, the silylidene ketal in **21** was removed and the resulting primary 23-OH was selectively protected with pivaloyl group to furnish **22** (81%). Coupling of 3-ol **22** with disaccharide *o*-alkynylbenzoate **11** under the optimized gold(I)-catalyzed conditions provided the desired glycoside **23** in excellent yield (95%). Ketone **23** was then subjected to reduction with NaBH_4_ followed by saponification with KOH at 55 °C to afford SSd (**2**) in 74% yield. Direct saponification of **23** furnished saikosaponin Y (**5**) (94%).

**Scheme 7:**
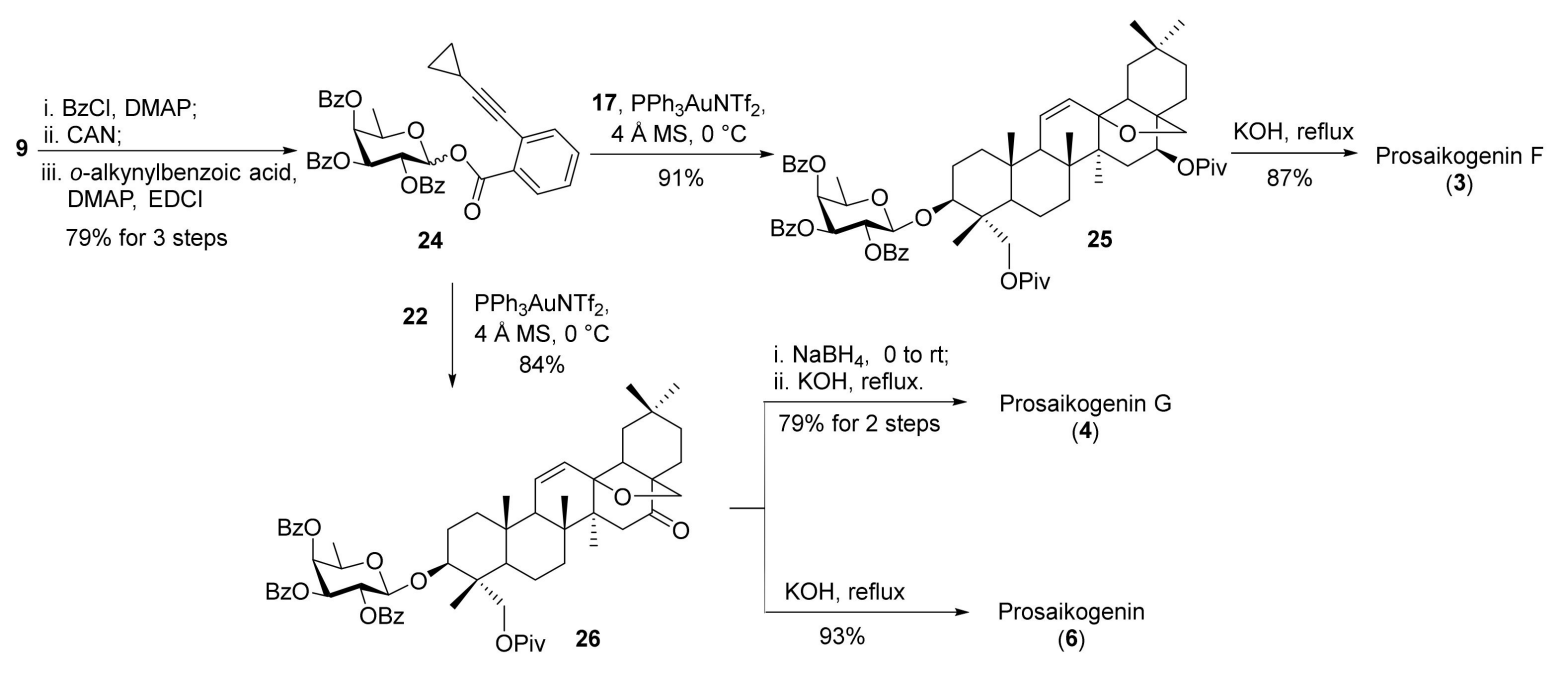
Synthesis of prosaikosaponin F (**3**), G (**4**), and prosaikogenin (**6**).

To synthesize monosaccharide saikosaponins **3**, **4**, and **6**, fucosyl *o*-alkynylbenzoate **24** was prepared from *p*-methoxyphenyl β-d-fucopyranoside **9** via protection with benzoyl groups, oxidative cleavage of the anomeric MP group, and esterification with *o*-alkynylbenzoic acid (79% for 3 steps) (Scheme 7). Glycosylation of aglycone derivatives **17** and **22** with *o*-alkynylbenzoate **24** led to glycosides **25** (91%) and **26** (84%). Saponification of **25** and **26** afforded prosaikogenin F (**3**) and prosaikogenin **6** in good yields. Alternatively, reduction of the 16-ketone in **26** with NaBH_4_ prior to saponification furnished prosaikogenin G (**4**) in 79% yield.

**Scheme 8:**
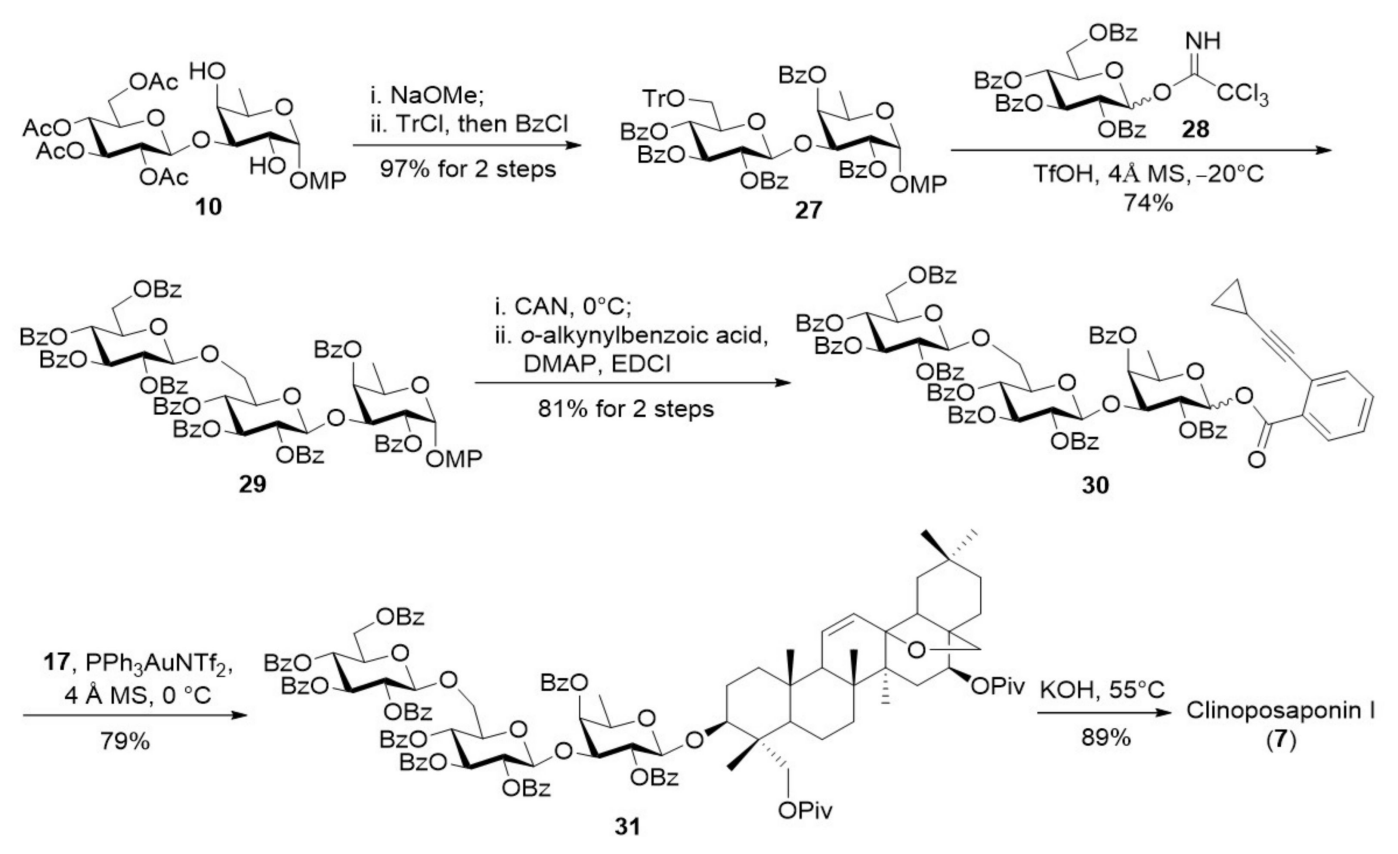
Synthesis of clinoposaponin I (**7**).

The synthesis of trisaccharide clinoposaponin I (**7**) was depicted in Scheme 8. Removal of the acetyl groups on disaccharide **10** followed by selective tritylation of the primary hydroxyl group and subsequent benzoylation led to disaccharide **27** (97%). Trityl ether **27** was successfully glycosylated with glucosyl trichloroacetimidate **28** under the action of TfOH (4 Å MS, CH_2_Cl_2_, −20 °C), affording trisaccharide **29** in 74% yield. Oxidative cleavage of the anomeric MP group on **29** followed by esterification provided the desired trisaccharide *o*-alkynylbenzoate **30** (81%). Under similar conditions as used in the previous glycosylations with mono- and disaccharide donors, coupling of aglycone **17** with trisaccharide *o*-alkynylbenzoate **30** led to the desired glycoside **31** in good yield (79%). Finally, saponification of **31** furnished clinoposaponin I (**7**) in 89% yield.

The analytical data of the synthetic saikosaponins **1**–**7** were in good agreement with those reported in the literatures for the natural products.

## 3. Experimental Section

### 3.1. General Information

Commercial reagents were used without further purification and made in China unless specified. Crushed 4Å molecular sieves (MS) were activated through flame-drying under high vacuum immediately prior to use. Dry CH_2_Cl_2_, DMF, and toluene were obtained by drying with activated MS. Dry pyridine and NEt_3_ were obtained by drying with anhydrous KOH. Anhydrous THF was obtained by refluxing with Na under argon. Thin layer chromatography (TLC) was performed on TLC silica gel 60 F254 (Merck, Darmstadt, Germany). The TLC plates were visualized with UV light and/or by staining with EtOH/H_2_SO_4_ (10%, *v*/*v*). Flash column chromatography was performed on silica gel SiliaFlash P60 (40−63 μm, Silicycle, Quebec, QC, Canada). NMR spectra were measured on a Bruker AM 400 (Switzerland), Agilent 500, or 600 MHz (U.S.) NMR spectrometer at 25 °C. ^1^H and ^13^C NMR signals were calibrated to the residual proton and carbon resonance of the solvent (CDCl_3_: *δ*H = 7.26 ppm, *δ*C = 77.16 ppm; CD_3_OD: *δ*H = 3.31 ppm, *δ*C = 49.00 ppm; DMSO-d6: *δ*H = 2.50 ppm, *δ*C = 39.52 ppm). High-resolution mass spectra were recorded with Shimadzu Biotech Axima Performance FTMS, maXis 4G FTMS, Thermo Scientific Q Exactive HF Orbitrap-FTMS, or Agilent-TOF/LC-MS 1260-6230 FTMS. Optical rotations were measured on an Anton Paar MCP5500 polarimeter.

### 3.2. Synthesis of Compounds **1**–**7**, **10**–**12**, **14**–**27**, and **29**–**31**

#### 3.2.1. Synthesis of Compound **10**

2,3,4,6-Tetra-*O*-acetyl-α-D-glucopyranosyl bromide **8** (115 mg, 0.3 mmol), compound **9** (50 mg, 0.2 mmol), silver(I) oxide (65 mg, 0.3 mmol), and 2-aminoethyl diphenylborinate (9 mg, 0.04 mmol) were added to an oven-dried round bottom flask under an argon atmosphere. Dry acetonitrile (1 mL) was added and the resulting mixture was stirred at room temperature. After 60 h, the reaction was quenched with a few drops of methanol, and the resulting mixture was diluted with CH_2_Cl_2_ and filtered through a plug of Celite. The crude product was purified by silica gel chromatography (petroleum ether/EtOAc = 2/1) to give compound **10** (76 mg, 85%) as a white foam. R*_f_* = 0.2 (silica, PE/EtOAc = 1:1); [α${]}_{D}^{25}$ = 82.8 (*c* = 1.0, CHCl_3_); ^1^H NMR (500 MHz,CDCl_3_): *δ* 7.08–6.93 (m, 2H), 6.87–6.75 (m, 2H), 5.41 (d, *J* = 4.1 Hz, 1H), 5.26 (m, 1H), 5.10–5.02 (m, 2H), 4.86 (d, *J* = 7.9 Hz, 1H), 4.24–4.20(m, 1H), 4.19–4.04 (m, 3H), 3.97 (m, 1H), 3.90 (s, 1H), 3.77 (d, *J* = 3.6 Hz, 1H), 3.76 (s, 3H), 2.08 (s, 3H), 2.07 (s,3H), 2.03 (s, 3H), 2.02 (s, 3H), 1.27 (d, *J* = 6.4 Hz, 3H). ^13^C NMR (125 MHz, CDCl_3_): *δ* 170.58, 170.18, 169.88, 169.41, 118.13, 114.64, 101.59, 98.42, 81.23, 72.30, 71.90, 71.40, 70.99, 68.46, 67.53, 66.19, 61.96, 55.62, 20.74, 20.69, 20.60, 20.57, 16.18. ESI-HRMS (*m*/*z*) calcd for C_27_H_36_NaO_15_ [M + Na]^+^ 623.1946, found 623.1951.

#### 3.2.2. Synthesis of Compound **11**

To a solution of compound **10** (63 mg, 0.1 mmol) in pyridine (1 mL), Ac_2_O (0.1 mL, 1.05 mmol) and DMAP (15 mg, 0.03 mmol) were added. The mixture was stirred at room temperature for 8 h before it was quenched with NaHCO_3_ and extracted with EtOAc. The combined organic phases were washed with brine, dried over anhydrous Na_2_SO_4_, filtered, and concentrated under vacuum. The residue was purified by flash column chromatography on silica gel (petroleum ether/EtOAc = 1/1) to give the corresponding acetylated product (73 mg, 96%) as a white solid.

To a stirred solution of the above product (50 mg, 0.07 mmol) in CH_3_CN/H_2_O (0.8 mL/0.2 mL), ceric ammonium nitrate (CAN) (89 mg, 0.16 mmol) was added. The mixture was stirred at room temperature for 2 h before it was quenched with Na_2_S_2_O_3_ and extracted with EtOAc. The combined organic phases were washed with brine, dried over anhydrous Na_2_SO_4_, filtered, and concentrated under vacuum. The residue was purified by flash column chromatography (petroleum ether/EtOAc, 1:1) to give the corresponding lactol product (33 mg, 80%) as an orange foam. The α/β anomers were difficult to separate.

The lactol (40 mg, 0.07 mmol), *ortho*-(cyclopropylethynyl) benzoic acid (18 mg, 0.09 mmol), EDCI (24 mg, 0.09mmol), and DMAP (12 mg, 0.09 mmol) were dissolved in CH_2_Cl_2_ (2 mL). The mixture was stirred at room temperature for 4 h and concentrated under vacuum. The residue was purified by flash chromatography (petroleum ether/EtOAc = 3:1) to give compound **11** (50 mg, 98%; α/β = 1:2.5) as a white foam.

**11α:** R*_f_* = 0.3 (silica, PE/EtOAc = 1:1); [α${]}_{D}^{25}$ = −3.3 (*c* = 1.0, CHCl_3_); ^1^H NMR (500 MHz, CDCl_3_): *δ* 7.94 (d, 1H), 7.55–7.44 (m, 2H), 7.42–7.30 (m, 1H), 6.56 (d, *J* = 3.6 Hz, 1H), 5.42 (d, *J* = 3.5 Hz, 1H), 5.37 (dd, *J* = 10.4, 3.6 Hz, 1H), 5.16–5.03 (m, 2H), 4.91 (dd, *J* = 9.4, 7.9 Hz, 1H), 4.69 (d, *J* = 7.9 Hz, 1H), 4.52 (q, *J* = 6.5 Hz, 1H), 4.37 (dd, *J* = 10.4, 3.5 Hz, 1H), 4.23 (m, 1H), 4.15 (m, 1H), 3.68 (m, 1H), 2.16 (s, 3H), 2.10 (s, 3H), 2.06 (s, 3H), 2.00 (s, 3H), 1.98 (s, 3H), 1.95 (s, 3H), 1.18 (d, *J* = 6.5 Hz, 3H), 1.0–0.8 (m, 4H). ^13^C NMR (125 MHz, CDCl_3_): *δ* 172.30, 170.92, 170.57, 170.45, 169.99, 169.45, 169.10, 164.64, 135.12, 132.37, 131.02, 127.50, 124.77, 75.18, 72.65, 72.54, 71.97, 71.40, 69.29, 68.63, 68.25, 61.53, 20.87, 20.77, 20.73, 20.72, 20.52, 16.28, 9.18, 9.16.

**11β:** R*_f_* = 0.25 (silica, PE/EtOAc = 1:1); [α${]}_{D}^{25}$ = −80.5 (*c* = 1.0, CHCl_3_); ^1^H NMR (500 MHz, CDCl_3_): *δ* 7.93 (d, *J* = 7.9 Hz, 1H), 7.46 (d, *J* = 7.6 Hz, 1H), 7.41 (t, *J* = 7.5 Hz, 1H), 7.28 (m, 1H), 5.82 (d, *J* = 8.3 Hz, 1H), 5.41 (t, *J* = 8.7 Hz, 1H), 5.30 (s, 1H), 5.10 (m, 2H), 4.91 (t, *J* = 8.4 Hz, 1H), 4.62 (d, *J* = 7.8 Hz, 1H), 4.26 (m, 1H), 4.15 (m, 1H), 3.94 (m, 2H), 3.69–3.58 (m, 1H), 2.15 (s, 3H), 2.10 (s, 3H), 1.99 (s, 9H), 1.97 (s, 3H), 1.51 (m, 1H), 1.20 (d, *J* = 5.3 Hz, 3H), 0.89 (m, 4H). ^13^C NMR (125 MHz, CDCl_3_): *δ* 170.84, 170.43, 170.41, 169.37, 169.32, 169.16, 163.64, 134.53, 132.58, 131.04, 129.35, 127.23, 125.79, 100.76, 100.45, 92.44, 74.46, 72.60, 71.95, 71.62, 71.19, 70.90, 70.00, 68.25, 61.43, 20.88, 20.76, 20.68, 20.48, 16.20, 9.02. ESI-HRMS (*m*/*z*) calcd for C_36_H_42_NaO_17_ [M + Na]^+^ 769.2314, found 7769.2311.

#### 3.2.3. Synthesis of Compound **12**

To a stirred solution of compound **10** (830 mg, 1.4 mmol) in pyridine (5 mL), benzoyl chloride (0.48 mL, 4.14 mmol) and DMAP (50 mg, 0.4 mmol) were added. The mixture was stirred at room temperature for 8 h before it was quenched with NaHCO_3_ and extracted with EtOAc. The combined organic phases were washed with brine, dried over anhydrous Na_2_SO_4_, and filtered. The filtrate was concentrated under vacuum. The residue was purified by flash column chromatography on silica gel (petroleum ether/EtOAc = 3/1) to give the corresponding benzoylated product (1.07 g, 96%) as a white solid.

To a stirred solution of the above product (800 mg, 0.99 mmol) in CH_3_CN/H_2_O (12 mL/3 mL), ceric ammonium nitrate (1.35 g, 2.5 mmol) was added. The mixture was stirred at room temperature for 2 h before it was quenched with Na_2_S_2_O_3_ and extracted with EtOAc. The combined organic phases were washed with brine, dried over anhydrous Na_2_SO_4_, filtered, and concentrated under vacuum. The residue was purified by flash column chromatography (petroleum ether/EtOAc = 1:1) to give the corresponding lactol product (560 mg, 81%) as an orange foam. The α/β anomers were difficult to separate.

The lactol (400 mg, 0.07 mmol), *ortho*-(cyclopropylethynyl) benzoic acid (167 mg, 0.89 mmol), EDCI (172 mg, 0.89 mmol), and DMAP (110 mg, 0.89 mmol) were dissolved in CH_2_Cl_2_ (5 mL). The mixture was stirred at room temperature for 4 h and then concentrated under vacuum. The residue was purified by flash chromatography (petroleum ether/EtOAc = 3:1) to give compound **12** (552 mg, 99%; α/β = 1:2.3) as a white foam.

**12α**: R*_f_* = 0.2 (silica, PE/EtOAc = 3:1); [α${]}_{D}^{25}$ = 107.9 (*c* = 1.0, CHCl_3_); ^1^H NMR (500 MHz, CDCl_3_): *δ* 8.11 (d, *J* = 7.5 Hz, 2H), 7.94 (d, *J* = 7.6 Hz, 2H), 7.90 (d, *J* = 7.8 Hz, 1H), 7.66–7.44 (m, 6H), 7.37 (m, 3H), 6.79 (d, *J* = 3.5 Hz, 1H), 5.84 (m, 1H), 5.75 (d, *J* = 2.5 Hz, 1H), 5.05–4.92 (m, 2H), 4.78 (m, 2H), 4.72–4.57 (m, 2H), 4.14 (m, 2H), 3.65 (m, 1H), 2.01 (s, 3H), 1.96 (s, 3H), 1.85 (s, 3H), 1.44 (m, 1H), 1.36 (s, 3H), 1.31–1.24 (m, 3H), 0.87 (m, 4H). ^13^C NMR (125 MHz, CDCl_3_): *δ* 170.90, 170.32, 169.32, 168.83, 166.02, 165.43, 164.59, 135.00, 133.62, 133.31, 132.32, 131.06, 130.95, 130.14, 129.83, 129.79, 129.29, 128.68, 128.60, 127.44, 124.76, 100.92, 99.57, 91.39, 75.07, 74.29, 73.08, 72.75, 71.85, 71.22, 69.36, 68.88, 68.15, 61.60, 20.80, 20.69, 20.60, 19.75, 16.66, 9.24, 9.19.

**12β:** R*_f_* = 0.15 (silica, PE/EtOAc = 3:1); [α${]}_{D}^{25}$ = 47.7 (*c* = 1.0, CHCl_3_); ^1^H NMR (500 MHz, CDCl_3_): *δ* 8.13 (d, *J* = 7.5 Hz, 2H), 7.97 (d, *J* = 7.6 Hz, 2H), 7.93 (d, *J* = 7.9 Hz, 1H), 7.57 (dt, *J* = 22.9, 7.4 Hz, 2H), 7.49 (t, *J* = 7.6 Hz, 2H), 7.41 (q, *J* = 7.7 Hz, 3H), 7.36 (m, 1H), 7.22 (d, *J* = 7.4 Hz, 1H), 6.10 (d, *J* = 8.4 Hz, 1H), 5.93–5.83 (m, 1H), 5.64 (d, *J* = 3.2 Hz, 1H), 5.03–4.93 (m, 2H), 4.79 (m, 1H), 4.67 (d, *J* = 7.8 Hz, 1H), 4.22 (m, 1H), 4.13 (m, 1H), 3.63 (d, *J* = 8.5 Hz, 1H), 2.02 (s, 3H), 1.96 (s, 3H), 1.86 (s, 3H), 1.50 (m, 1H), 1.44 (s, 3H), 1.32 (d, *J* = 6.3 Hz, 3H), 0.85 (m, 4H). ^13^C NMR (125 MHz, CDCl_3_): *δ* 170.91, 170.35, 169.32, 168.93, 166.14, 165.10, 163.84, 134.44, 133.63, 133.27, 132.48, 131.09, 130.23, 129.81, 129.48, 129.32, 128.71, 128.53, 127.20, 125.76, 110.13, 101.15, 100.42, 92.71, 78.23, 74.46, 72.78, 72.53, 71.86, 71.46, 71.12, 70.33, 68.08, 61.47, 31.75, 29.77, 20.82, 20.67, 20.60, 19.79, 16.65. ESI-HRMS (*m*/*z*) calcd for C_46_H_46_NaO_17_ [M + Na]^+^ 893.2627, found 893.2632.

#### 3.2.4. Synthesis of Compound **14**

Compound **13** (100 mg, 0.17 mmol) was dissolved in pyridine (5 mL), to which was added HF·pyr (0.2 mL) at room temperature. After stirring at 90 °C for 24 h, the mixture was quenched with saturated NaHCO_3_ and extracted with EtOAc. The combined organic phases were washed with brine, dried over anhydrous Na_2_SO_4_, filtered, and concentrated under vacuum. The residue was purified by flash column chromatography (petroleum ether/EtOAc = 2:1) to give the 3-ol product (76 mg, 88%) as a white foam. R*_f_* = 0.2 (silica, PE/EtOAc = 5:1).

The above product (3.6 g, 7.2 mmol) was dissolved in CH_2_Cl_2_ (40 mL), to which Dess-Martin periodinane (DMP) (3.7 g, 8.6 mmol) was added. After being stirred for 2 h at room temperature, the reaction mixture was quenched with a saturated aqueous Na_2_S_2_O_3_ solution, and the aqueous phase was extracted with CH_2_Cl_2_. The combined organic extracts were dried over anhydrous Na_2_SO_4_ and concentrated in vacuo.

The crude product was dissolved in MeOH/CH_2_Cl_2_ (80 mL/40 mL), to which NH_2_OH·HCl (692 mg, 10.8 mmol) and NaOAc (1.76 g, 21.6 mmol) were added at room temperature. After stirring two hours at reflux, the reaction mixture was diluted by addition of brine, then extracted with CH_2_Cl_2_. The extract was dried over anhydrous Na_2_SO_4_, followed by filtration and concentration in vacuo. The residue was purified by silica gel flash column chromatography (petroleum ether/EtOAc = 6/1 to 3/1) to give compound **14** (3.55 g, 96%) as a white foam. R*_f_* = 0.5 (silica, PE/EtOAc = 2:1); [α${]}_{D}^{25}$ = 76.7 (*c* = 1.0, CHCl_3_); ^1^H NMR (500 MHz, CDCl_3_): *δ* 9.30–9.23 (m, 1H), 5.88–5.86 (m, 1H), 5.43–5.38 (m, 2H), 3.97 (d, *J* = 7.4 Hz, 1H), 3.18 (d, *J* = 7.6, 1H), 3.11–3.07 (m, 1H), 2.26–2.19 (m, 1H), 2.04 (s, 3H), 1.14 (s, 3H), 1.10 (s, 3H), 1.03 (s, 3H), 1.00 (s, 3H), 0.99 (s, 3H), 0.96 (s, 3H), 0.88 (s, 3H). ^13^C NMR (125 MHz, CDCl_3_): *δ* 170.80, 166.65, 132.60, 129.92, 83.88, 73.24, 68.79, 55.34, 52.16, 51.68, 45.34, 45.27, 41.88, 40.61, 38.39, 37.41, 36.51, 34.28, 33.44, 31.97, 31.52, 31.12, 26.95, 25.44, 23.76, 22.66, 21.34, 20.47, 19.43, 18.34, 17.37, 16.94. ESI-HRMS (*m*/*z*) calcd for C_32_H_50_NO_4_ [M + H]^+^ 512.3734, found 512.3739.

#### 3.2.5. Synthesis of Compound **15**

To a solution of compound **14** (1.9 g, 3.62 mmol) in HOAc (170 mL), NaOAc (350 mg, 4.3 mmol) and Na_2_PdCl_4_ (1.3 g, 4.3 mmol) were added at room temperature. The mixture was stirred at room temperature for 3 d, and then ice water (300 mL) was added. The resulting mixture was stirred for an additional 20 min and then filtered. The filtered residue was washed with water several times, dried, and used without further purification.

The residue was dissolved in CH_2_Cl_2_ (20 mL) at room temperature, to which NEt_3_ (1 mL, 7.24 mmol), DMAP (88 mg, 0.72 mmol), and Ac_2_O (0.68 mL, 7.24 mmol) were added. The resulting mixture was stirred at room temperature for 3 h and then diluted with EtOAc. The mixture was thoroughly washed with water and brine, and the organic layer was dried with anhydrous Na_2_SO_4_. After filtration, the solution was concentrated under vacuum to give a residue. The residue was dissolved in THF (120 mL), and anhydrous pyridine (0.3 mL) was added dropwise. After the mixture was stirred for 40 min, a solution of Pb(OAc)_4_ (1.5 mg, 3.2 mmol) in HOAc (51 mL) was added dropwise at −78 °C. The mixture was warmed to room temperature and then stirred for 24 h. The reaction mixture was cooled to 0 °C, and a solution of NaBH_4_ (136 mg, 1.7 mmol) in NaOH (1M, 46 mL) was slowly added. The resulting solution was stirred at 0 °C for an additional 1 h and diluted with EtOAc. The mixture was washed with a saturated NaHCO_3_ solution and then brine, and the organic layer was dried with anhydrous Na_2_SO_4_. After filtration, the solution was concentrated to give a residue, which was purified by flash column chromatography on silica gel (petroleum/EtOAc = 7:1) to give compound **15** (1.4 g, 63% for 3 steps) as a white foamy solid. R*_f_* = 0.3 (silica, PE/EtOAc = 4:1); [α${]}_{D}^{25}$ = 98.9 (*c* = 1.0, CHCl_3_); ^1^H NMR (500 MHz, CDCl_3_): *δ* 5.81 (d, *J* = 10.3 Hz, 1H), 5.44–5.36 (m, 2H), 4.17–4.07 (m, 2H), 3.95 (d, *J* = 7.5 Hz, 1H), 3.16 (d, *J* = 7.5 Hz, 1H), 2.80–2.75 (m, 1H), 2.57–2.50 (m, 1H), 2.13 (s, 3H), 2.02 (d, *J* = 3.1 Hz, 6H), 1.12 (s, 3H), 1.08 (s, 3H), 1.00 (s, 3H), 0.93 (s, 6H), 0.86 (s, 3H). ^13^C NMR (126 MHz, CDCl_3_): *δ* 170.88, 170.67, 170.06, 169.77, 131.93, 130.23, 83.63, 73.20, 68.54, 67.63, 51.72, 51.63, 47.88, 45.24, 45.19, 44.09, 41.60, 37.36, 37.01, 35.91, 34.17, 33.37, 31.85, 31.45, 30.44, 25.34, 23.69, 21.26, 21.01, 20.40, 20.06, 19.95, 19.02, 18.71, 18.56, 17.44. ESI-HRMS (*m*/*z*) calcd for C_36_H_53_NNaO_7_ [M + Na]^+^ 634.3714, found 634.3717.

#### 3.2.6. Synthesis of Compound **16**

A solution of compound **15** (1.0 g, 1.6 mmol) and Na_2_CO_3_ (866 mg, 8.2 mmol) in MeOH (120 mL) was stirred at room temperature for 24 h. After removing MeOH in vacuo, the resulting colorless solid was dissolved in Et_2_O. The organic layer was washed with 1 N HCl solution, saturated NaHCO_3_ solution and brine, dried over anhydrous Na_2_SO_4_, and filtered. The filtrate was evaporated in vacuo to give a solid.

To a solution of the above product in THF (55 mL), NH_4_OAc (1.5 g, 20 mmol) and a buffered solution of TiCl_3_ (3.4 mL of HCl solution containing 15% TiCl_3_, 4 mmol) were added. The mixture was stirred at room temperature for 8 h. The mixture was washed with saturated NaHCO_3_ solution and brine, dried over anhydrous Na_2_SO_4_, filtered, and evaporated in vacuo to give a solid. The solid was purified by silica gel flash column chromatography (petroleum ether/EtOAc = 2/1) to give compound **16** (613 mg, 80%) as a white foam. R*_f_* = 0.5 (silica, PE/EtOAc = 1:1); [α${]}_{D}^{25}$ = 89.4 (*c* = 1.0, CHCl_3_); ^1^H NMR (500 MHz, CDCl_3_): *δ* 5.91–5.81 (m, 1H), 5.47 (dd, *J* = 10.3, 3.1 Hz, 1H), 4.21 (dd, *J* = 10.1, 5.9 Hz, 1H), 3.92 (d, *J* = 7.4 Hz, 1H), 3.70 (d, *J* = 11.4 Hz, 1H), 3.40 (d, *J* = 11.4 Hz, 1H), 3.13 (d, *J* = 7.5 Hz, 1H), 2.68 (m, 1H), 2.31 (m, 1H), 2.11 (m, 1H), 1.14 (s, 3H), 1.11 (s, 3H), 1.01 (s, 3H), 1.00 (s. 3H), 0.99 (s, 3H), 0.91 (s, 3H). ^13^C NMR (125 MHz, CDCl_3_): *δ* 218.37, 131.71, 130.37, 72.28, 66.39, 64.99, 52.75, 51.80, 51.52, 48.41, 46.21, 45.51, 41.70, 38.73, 37.33, 35.91, 35.29, 35.01, 34.17, 33.53, 31.46, 30.67, 29.69, 24.94, 23.67, 20.61, 19.36, 18.31, 17.28, 16.25. ESI-HRMS (*m*/*z*) calcd for C_30_H_46_NaO_4_ [M + Na]^+^ 493.3288, found 493.3283.

#### 3.2.7. Synthesis of Compound **17**

To a stirred solution of compound **16** (90 mg, 0.2 mmol) in pyridine (4 mL), pivaloyl chloride (0.5 mL, 4 mmol) and DMAP (6 mg, 0.06 mmol) were added. The mixture was stirred at room temperature for 8 h before it was quenched with NaHCO_3_ and extracted with EtOAc. The combined organic phases were washed with brine, dried over anhydrous Na_2_SO_4_, filtered, and concentrated under vacuum. The residue was purified by flash column chromatography on silica gel (petroleum ether/EtOAc = 8/1) to give the acylated product (115 mg, 94%) as a white solid. R*_f_* = 0.5 (silica, PE/EtOAc = 3:1); [α${]}_{D}^{25}$ = 113.9 (*c* = 1.0, CHCl_3_); ^1^H NMR (500 MHz, CDCl_3_): *δ* 5.90 (d, *J* = 10.3 Hz, 1H), 5.48 (dd, *J* = 10.3, 3.1 Hz, 1H), 5.39 (m, 1H), 4.13–4.03 (m, 2H), 4.00 (d, *J* = 7.4 Hz, 1H), 3.22 (d, *J* = 7.1 Hz, 1H), 2.60 (m, 1H), 2.46 (m, 1H), 1.20 (s, 9H), 1.17 (s, 9H), 1.15 (s, 3H), 1.06 (s, 3H), 1.05 (s, 3H), 1.00 (s, 3H), 0.97 (s, 3H), 0.90 (s, 3H). ^13^C NMR (125 MHz, CDCl_3_): *δ* 213.52, 178.05, 177.81, 130.99, 83.74, 73.20, 68.03, 66.70, 51.75, 51.65, 50.54, 47.52, 45.34, 45.21, 41.61, 38.93, 38.77, 37.66, 37.33, 35.65, 34.58, 34.03, 33.31, 31.57, 31.39, 30.47, 27.20, 25.20, 23.65, 20.27, 19.18, 18.56, 16.97, 16.56. ESI-HRMS (*m*/*z*) calcd for C_40_H_62_NaO_6_ [M + Na]^+^ 661.4439, found 661.4445.

To a stirred solution of the above product (90 mg, 0.14 mmol) in MeOH (3 mL) at 0 °C, NaBH_4_ (23 mg, 0.5 mmol) was added. The stirring continued for 15 min, the solution was quenched by addition of saturated NH_4_Cl solution, then diluted with brine and extracted with CH_2_Cl_2_. The extract was dried over anhydrous Na_2_SO_4_, filtered, and concentrated in vacuo. The residue was purified by silica gel column chromatography (petroleum ether/EtOAc = 4/1) to give compound **17** (76 mg, 85%) as a white foam. R*_f_* = 0.3 (silica, PE/EtOAc = 3:1); [α${]}_{D}^{25}$ = 65.3 (*c* = 1.0, CHCl_3_); ^1^H NMR (500 MHz, CDCl_3_): *δ* 5.88 (d, *J* = 10.3 Hz, 1H), 5.42 (dd, *J* = 10.2, 3.0 Hz, 1H), 5.40–5.35 (m, 1H), 4.19 (d, *J* = 11.6 Hz, 1H), 3.99 (d, *J* = 7.4 Hz, 1H), 3.75 (d, *J* = 11.5 Hz, 1H), 3.36 (t, *J* = 8.2 Hz, 1H), 3.21 (d, *J* = 7.4 Hz, 1H), 2.33 (t, *J* = 7.5 Hz, 1H), 1.21 (s, 9H), 1.20 (s, 9H), 1.09 (s, 3H), 1.03 (s, 3H), 0.96 (s, 3H), 0.93 (s, 3H), 0.88 (s, 3H), 0.75 (s, 3H). ^13^C NMR (125 MHz, CDCl_3_): *δ* 178.76, 178.14, 132.52, 129.64, 83.88, 73.14, 72.26, 68.19, 65.77, 52.75, 51.62, 47.73, 45.31, 45.17, 42.42, 41.68, 38.32, 37.32, 36.08, 34.05, 33.32, 31.56, 31.38, 31.05, 29.68, 27.31, 27.19, 25.91, 25.21, 23.64, 20.44, 19.43, 18.16, 17.37, 11.39. ESI-HRMS (*m*/*z*) calcd for C_40_H_64_NaO_6_ [M + Na]^+^ 663.4595, found 663.4598.

#### 3.2.8. Synthesis of Compound **18**

To a stirred mixture of compound **11** (180 mg, 0.24 mmol), compound **17** (120 mg, 0.18 mmol), and 4Å molecular sieves (400 mg) in CH_2_Cl_2_ (4 mL), PPh_3_AuNTf_2_ (26 mg, 0.04 mmol) was added. The mixture was stirred at room temperature for 1 h before it was quenched with NEt_3_. The mixture was filtered through a pad of celite and washed with EtOAc. The filtrate was concentrated under vacuum. The residue was purified by flash column chromatography (petroleum ether/EtOAc = 4:1) to give compound **18** (190 mg, 84%) as a white foam. R*_f_* = 0.3 (silica, PE/EtOAc = 3:1); [α${]}_{D}^{25}$ = 43.9 (*c* = 1.0, CHCl_3_); ^1^H NMR (600 MHz, CDCl_3_): *δ* 5.91–5.78 (m, 1H), 5.47–5.38 (m, 1H), 5.36 (dd, *J* = 9.6, 6.2 Hz, 1H), 5.18 (d, *J* = 3.5 Hz, 1H), 5.10 (m, 3H), 4.87 (t, *J* = 8.5 Hz, 1H), 4.58 (d, *J* = 7.9 Hz, 1H), 4.26 (m, 2H), 4.13 (m, 1H), 3.97 (d, *J* = 7.4 Hz, 1H), 3.81 (d, *J* = 11.3 Hz, 1H), 3.77–3.71 (m, 2H), 3.66–3.62 (m, 1H), 3.61 (d, *J* = 6.5 Hz, 1H), 3.36 (dd, *J* = 11.5, 4.7 Hz, 1H), 3.19 (d, *J* = 7.4 Hz, 1H), 2.11 (s, 3H), 2.10 (s, 3H), 2.09 (s, 3H), 2.00 (s, 3H), 1.99 (s, 3H), 1.97 (s, 3H), 1.24 (s, 9H), 1.19 (s, 9H), 1.17 (s, 3H), 1.07 (s, 3H), 1.00 (s, 3H), 0.95 (s, 3H), 0.90 (s, 3H), 0.88 (s, 3H), 0.71 (s, 3H). ^13^C NMR (150 MHz, CDCl_3_): *δ* 102.85, 100.52, 73.15, 72.60, 71.75, 71.68, 71.26, 71.10, 69.30, 68.31, 68.19, 65.00, 61.31, 52.72, 51.62, 47.66, 45.32, 45.17, 42.04, 41.69, 39.06, 38.96, 38.11, 37.35, 35.79, 34.08, 33.36, 31.55, 31.41, 30.98, 27.38, 27.32, 27.20, 25.28, 25.23, 23.65, 20.98, 20.76, 20.59, 20.40, 20.37, 19.44, 17.94, 17.22, 16.35, 12.17. ESI-HRMS (*m*/*z*) calcd for C_64_H_96_NaO_21_ [M + Na]^+^ 1223.6336, found 1223.6318.

#### 3.2.9. Synthesis of Saikosaponin A (**1**)

To a stirred solution of compound **18** (110 mg, 0.1 mmol) in MeOH (3 mL), KOH (261 mg, 4.6 mmol) was added at room temperature. The mixture was stirred at reflux for 24 h before it was quenched with acetic acid. The mixture was concentrated under vacuum. The residue was purified by reversed-phase silica gel column chromatography (ODS RP-18) (MeOH/H_2_O = 5:1 to 7:1) to give saikosaponin A (**1**) (69 mg, 93%) as a white powder. Rf = 0.4 (silica, CHCl_3_/MeOH = 5:1); [α${]}_{D}^{25}$ = −5.9 (*c* = 1.0, CH_3_OH); ^1^H NMR (600 MHz, pyridine-*d*_5_): *δ* 6.07–5.99 (m, 1H), 5.73–5.63 (m, 1H), 5.35 (d, *J* = 7.8 Hz, 1H), 5.00 (d, *J* = 7.8 Hz, 1H), 4.57 (m, 4H), 4.45–4.37 (m, 4H), 4.30 (m, 2H), 4.23 (m, 1H), 4.09–4.01 (m, 3H), 3.75–3.67 (m, 2H), 3.36 (d, *J* = 6.9 Hz, 1H), 2.52 (m, 1H), 2.35 (m, 1H), 1.45 (d, *J* = 6.3 Hz, 3H), 1.41 (s, 3H), 1.12 (s, 3H), 1.01 (s, 3H), 0.95 (s, 3H), 0.94 (s, 3H), 0.91 (s, 3H). ^13^C NMR (150 MHz, Pyridine-*d*_5_): *δ* 132.14, 131.11, 106.58, 105.95, 85.12, 83.94, 81.53, 78.71, 78.36, 75.76, 72.97, 72.09, 71.77, 71.49, 70.96, 64.00, 63.92, 62.64, 53.03, 52.09, 47.23, 46.94, 45.58, 43.68, 42.14, 38.60, 37.67, 36.20, 36.06, 34.62, 33.59, 31.55, 31.52, 26.06, 25.70, 23.77, 20.79, 19.99, 18.69, 17.49, 17.22, 13.00. ESI-HRMS (*m*/*z*) calcd for C_42_H_68_NaO_13_ [M + Na]^+^ 803.4552, found 803.4549.

#### 3.2.10. Synthesis of Compound **19**

Compound **16** (290 mg, 0.6 mmol) was dissolved in CH_2_Cl_2_ (10 mL), to which DMP (636 mg, 1.5 mmol) was added. After being stirred for 40 min at room temperature, the reaction mixture was quenched with a saturated Na_2_S_2_O_3_ solution and the aqueous phase was extracted with CH_2_Cl_2_. The combined organic extracts were dried over anhydrous Na_2_SO_4_ and concentrated in vacuo.

To a solution of the above product in MeOH (10 mL) under stirring at 0 °C, NaBH_4_ (95 mg, 2.5 mmol) was added. The mixture was stirred for 15 min before it was quenched by addition of saturated NH_4_Cl solution. The mixture was diluted with brine and extracted with CH_2_Cl_2_. The combined organic phases were dried over anhydrous Na_2_SO_4_, filtered, and concentrated in vacuo. The residue was purified by silica gel column chromatography (petroleum ether/EtOAc = 2/1) to give compound **19** (190 mg, 65%) as a white foam. R*_f_* = 0.3 (silica, PE/EtOAc = 2:1); [α${]}_{D}^{25}$ = 11.2 (*c* = 1.0, Pyridine); ^1^H NMR (500 MHz, Pyridine-*d*_5_): *δ* 6.05 (m, 1H), 5.74 (d, *J* = 8.8 Hz, 1H), 3.74 (d, *J* = 9.8 Hz, 1H), 3.62 (d, *J* = 5.6 Hz, 1H), 3.36 (d, *J* = 5.4 Hz, 1H), 2.69 (m, 1H), 2.52 (m, 1H), 1.64 (s, 3H), 1.41 (s, 3H), 1.10 (s, 6H), 1.06 (s, 3H), 1.00 (s, 3H). ^13^C NMR (125 MHz, Pyridine-*d*_5_): *δ* 132.46, 132.39, 85.30, 78.25, 77.58, 73.76, 68.15, 53.43, 51.80, 48.87, 45.79, 44.05, 43.50, 42.31, 39.10, 38.87, 37.25, 37.02, 35.88, 34.21, 32.34, 32.03, 31.74, 28.03, 24.89, 19.99, 19.14, 18.55, 18.44, 12.98. ESI-HRMS (*m*/*z*) calcd for C_30_H_48_NaO_4_ [M + Na]^+^ 495.3445, found 495.3437.

#### 3.2.11. Synthesis of Compound **20**

To a stirred solution of compound **19** (187 mg, 0.4 mmol) in CH_2_Cl_2_ (4 mL), pivaloyl chloride (58 μL, 0.48 mmol) and pyridine (0.57 mL, 7.2 mmol) were added. The mixture was stirred at −10 °C for 8 h before it was quenched with NaHCO_3_ and extracted with EtOAc. The combined organic phases were washed with brine, dried over anhydrous Na_2_SO_4_, filtered, and concentrated under vacuum. The residue was purified by flash column chromatography on silica gel (petroleum ether/EtOAc = 8/1) to give compound **20** (178 mg, 81%) as a white solid. R*_f_* = 0.3 (silica, PE/EtOAc = 3:1); [α${]}_{D}^{25}$ = 41.7 (*c* = 1.0, CHCl_3_); ^1^H NMR (500 MHz, CDCl_3_): *δ* 5.87 (d, *J* = 10.4 Hz, 1H), 5.42 (dd, *J* = 10.3, 3.0 Hz, 1H), 4.17 (d, *J* = 11.5 Hz, 1H), 4.00 (d, *J* = 4.9 Hz, 1H), 3.78 (d, *J* = 11.5 Hz, 1H), 3.45 (d, *J* = 7.3 Hz, 1H), 3.40–3.36 (m, 1H), 3.17 (d, *J* = 7.3 Hz, 1H), 2.20 (m, 1H), 1.25 (s, 3H), 1.21 (s, 9H), 1.05 (s, 3H), 0.95 (s, 3H), 0.93 (s, 3H), 0.91 (s, 3H), 0.75 (s, 3H). ^13^C NMR (125 MHz, CDCl_3_): *δ* 178.84, 132.14, 130.83, 84.74, 78.12, 72.49, 66.10, 52.88, 50.47, 47.92, 45.00, 42.98, 42.57, 41.46, 39.16, 38.41, 38.13, 36.79, 36.25, 35.10, 33.55, 31.72, 31.17, 30.47, 29.82, 27.47, 27.19, 26.13, 24.26, 19.11, 18.36, 18.00, 17.61, 11.52. ESI-HRMS (*m*/*z*) calcd for C_35_H_56_NaO_5_ [M + Na]^+^ 579.4020, found 579.4016.

#### 3.2.12. Synthesis of Compound **21**

A solution of tetramethylammonium triacetoxyborohydride (1.9 g, 7.2 mmol) in MeCN (27 mL) and AcOH (1.12 mL) was cooled to −40 °C. To the mixture was added a solution of the compound **16** (680 mg, 1.4 mmol) in CH_2_Cl_2_ (9 mL). The mixture was stirred at room temperature for 0.5 h before it was quenched with Rochelle salt (sat. aq.) and extracted with EtOAc. The combined organic phases were washed with brine, dried over anhydrous Na_2_SO_4_, filtered, and concentrated under vacuum.

The above product was dissolved into DMF (5 mL), to which 2,6-lutidine (2.5 mL) was added, and the solution was cooled to 0 °C. Di-*tert*-butylsilylbis(trifluoromethansulfonate) (0. 6 mL, 1.7 mmol) was added. The mixture was stirred at room temperature for 2 h before it was quenched with NaHCO_3_ and extracted with EtOAc. The combined organic phases were washed with brine, dried over anhydrous Na_2_SO_4_, filtered, and concentrated under vacuum.

The above product was dissolved in CH_2_Cl_2_ (5 mL), then the solution was added DMP (736 mg, 1.7 mmol). After being stirred for 40 min at room temperature, the reaction mixture was quenched with a saturated Na_2_S_2_O_3_ solution, and the aqueous phase was extracted with CH_2_Cl_2_. The combined organic extracts were dried over anhydrous Na_2_SO_4_ and concentrated in vacuo. The residue was purified by flash chromatography (petroleum ether/EtOAc = 15:1) to give compound **21** (507 mg, 74%) as a white solid. R*_f_* = 0.5 (silica, PE/EtOAc = 6:1); [α${]}_{D}^{25}$ = 12.9 (*c* = 1.0, CHCl_3_); ^1^H NMR (500 MHz, CDCl_3_): *δ* 5.95 (d, *J* = 10.3 Hz, 1H), 5.56–5.52 (m, 1H), 3.86 (m, 3H), 3.72 (d, *J* = 10.3 Hz, 1H), 3.44 (d, *J* = 7.9 Hz, 1H), 2.68 (m, 1H), 2.23 (m, 1H), 2.15 (m, 1H), 1.09 (s, 9H), 1.07 (s, 3H), 1.05 (s, 3H), 1.03 (s, 9H), 0.98 (s, 3H), 0.95 (s, 3H), 0.92 (s, 3H), 0.86 (s, 3H). ^13^C NMR (125 MHz, CDCl_3_): *δ* 213.14, 213.12, 133.20, 128.84, 84.15, 80.53, 75.56, 56.35, 55.03, 52.37, 52.28, 49.63, 44.49, 41.78, 41.04, 38.89, 38.79, 36.64, 35.54, 33.40, 31.68, 31.09, 29.24, 27.94, 27.83, 26.68, 24.31, 23.70, 23.29, 20.50, 20.23, 19.52, 18.75, 17.14, 12.28. ESI-HRMS (*m*/*z*) calcd for C_38_H_63_O_4_Si [M + H]^+^ 611.4490, found 611.4488.

#### 3.2.13. Synthesis of Compound **22**

The compound **21** was dissolved in THF (5 mL), to which HF·pyr (1 mL) was added at room temperature. After stirring at room temperature for 1 h, the mixture was quenched with saturated NaHCO_3_ and extracted with EtOAc. The combined organic phases were washed with brine, dried over anhydrous Na_2_SO_4_, filtered, and concentrated under vacuum. The residue was purified by flash column chromatography (petroleum ether/EtOAc = 2:1) to give the diol product (500 mg, 99%) as a white foam. R*_f_* = 0.3 (silica, PE/EtOAc = 3:1); [α${]}_{D}^{25}$ = 12.9 (*c* = 1.0, Pyridine); ^1^H NMR (500 MHz, Pyridine-*d*_5_): *δ* 6.11 (d, *J* = 10.4 Hz, 1H), 5.74 (m, 1H), 4.26 (dd, *J* = 11.2, 5.3 Hz, 1H), 4.22 (d, *J* = 10.4 Hz, 1H), 3.96 (d, *J* = 7.8 Hz, 1H), 3.74 (d, *J* = 10.4 Hz, 1H), 3.54 (d, *J* = 7.8 Hz, 1H), 2.87 (m, 1H), 2.36 (m, 1H), 1.42 (s, 3H), 1.07 (s, 3H), 1.05 (s, 3H), 1.03 (s, 3H), 0.87 (s, 3H), 0.83 (s, 3H). ^13^C NMR (125 MHz, pyridine-*d*_5_): *δ* 212.80, 133.74, 129.94, 84.73, 75.90, 73.18, 67.47, 56.85, 55.59, 53.25, 50.29, 48.50, 45.25, 43.62, 42.56, 39.47, 39.02, 36.97, 36.36, 33.78, 32.12, 31.86, 28.05, 24.97, 23.60, 20.70, 20.28, 18.98, 18.28, 13.08. ESI-HRMS (*m/z*) calcd for C_30_H_47_O_4_ [M + H]^+^ 471.3469, found 471.3463.

To a stirred solution of the above product (470 mg, 1 mmol) in CH_2_Cl_2_ (10 mL), pivaloyl chloride (0.15 mL, 1.2 mmol) and pyridine (0.8 mL, 12 mmol) were added. The mixture was stirred at −10 °C for 30 h before it was quenched with NaHCO_3_ and extracted with EtOAc. The combined organic phases were washed with brine, dried over anhydrous Na_2_SO_4_, filtered, and concentrated under vacuum. The residue was purified by flash column chromatography on silica gel (petroleum ether/EtOAc = 9/1) to give compound **22** (487 mg, 81%) as a white solid. R*_f_* = 0.4 (silica, PE/EtOAc = 3:1); [α${]}_{D}^{25}$ = 12.9 (*c* = 1.0, CHCl_3_); ^1^H NMR (500 MHz, CDCl_3_): *δ* 5.94 (d, *J* = 10.3 Hz, 1H), 5.52 (dd, *J* = 10.3, 2.8 Hz, 1H), 4.15 (d, *J* = 11.5 Hz, 1H), 3.85 (d, *J* = 8.0 Hz, 1H), 3.75 (d, *J* = 11.5 Hz, 1H), 3.41 (t, *J* = 11.6 Hz, 1H), 3.37–3.28 (m, 1H), 2.75–2.56 (m, 1H), 1.18 (s, 9H), 1.16 (s, 3H), 0.95 (s, 3H), 0.92 (s, 3H), 0.88 (s, 3H), 0.83 (s, 3H), 0.73 (s, 3H). ^13^C NMR (125 MHz, CDCl_3_): *δ* 213.04, 178.70, 133.13, 128.79, 84.14, 75.50, 72.23, 65.91, 56.26, 54.97, 52.70, 49.55, 47.87, 44.39, 42.47, 41.69, 39.09, 38.85, 38.35, 36.17, 35.47, 33.33, 31.57, 31.08, 27.37, 26.00, 24.21, 23.21, 20.11, 19.46, 18.16, 17.44, 11.50. ESI-HRMS (*m*/*z*) calcd for C_35_H_54_NaO_5_ [M + Na]^+^ 577.3863, found 577.3868.

#### 3.2.14. Synthesis of Compound **23**

To a stirred mixture of compound **22** (50 mg, 0.09 mmol), compound **12** (102 mg, 0.11 mmol), and 4Å molecular sieves (200 mg) in CH_2_Cl_2_ (2 mL), PPh_3_AuNTf_2_ (13 mg, 0.02 mmol) was added. The mixture was stirred at room temperature for 1 h before it was quenched with NEt_3_. The mixture was filtered through a pad of celite and washed with EtOAc. The filtrate was concentrated under vacuum. The residue was purified by flash column chromatography (petroleum ether/EtOAc = 3:1) to give compound **23** (107 mg, 95%) as a white foam. R*_f_* = 0.3 (silica, PE/EtOAc = 4:1); [α${]}_{D}^{25}$ = 53.3 (*c* = 1.0, CHCl_3_); ^1^H NMR (500 MHz, CDCl_3_): *δ* 8.09 (d, *J* = 7.3 Hz, 4H), 7.60–7.56 (m, 2H), 7.47 (t, *J* = 6.7 Hz, 4H), 5.93 (d, *J* = 10.4 Hz, 1H), 5.60–5.45 (m, 3H), 5.04–4.86 (m, 3H), 4.75 (m, 2H), 4.61 (d, *J* = 7.8 Hz, 1H), 4.50 (d, *J* = 7.9 Hz, 1H), 4.19 (d, *J* = 10.1 Hz, 2H), 4.14–4.09 (m, 2H), 4.04 (m, 1H), 3.85 (d, *J* = 7.8 Hz, 1H), 3.77 (t, *J* = 9.7 Hz, 2H), 3.59 (d, *J* = 9.2 Hz, 1H), 3.51 (d, *J* = 11.6 Hz, 1H), 3.45–3.37 (m, 2H), 2.66 (m, 1H), 2.22 (m, 1H), 2.13 (m, 1H), 1.98 (s, 3H), 1.94 (s, 3H), 1.83 (s, 3H), 1.44 (s, 3H), 1.25 (d, J = 6.0 Hz, 3H), 1.21 (s, 9H), 1.12 (s, 3H), 0.93 (s, 3H), 0.89 (s, 3H), 0.88 (s, 3H), 0.85 (s, 3H), 0.50 (s, 3H). ^13^C NMR (125 MHz, CDCl_3_): *δ* 213.11, 177.70, 170.94, 170.33, 169.31, 168.99, 166.38, 165.05, 133.54, 133.21, 133.15, 130.27, 129.97, 129.56, 128.74, 128.46, 102.87, 101.00, 84.16, 81.63, 78.02, 75.55, 72.82, 71.74, 71.66, 71.08, 69.97, 68.10, 64.75, 61.43, 56.30, 55.01, 52.71, 49.60, 47.70, 44.42, 42.22, 41.70, 39.15, 38.89, 38.26, 35.86, 35.52, 33.38, 31.63, 31.00, 27.49, 27.43, 25.46, 24.27, 23.24, 20.78, 20.66, 20.59, 20.08, 19.78, 19.47, 17.86, 17.21, 16.89, 12.01. ESI-HRMS (*m*/*z*) calcd for C_69_H_90_NaO_20_ [M + Na]^+^ 1261.5918, found 1261.5925.

#### 3.2.15. Synthesis of Saikosaponin D (**2**)

To a solution of compound **23** (102 mg, 0.08 mmol) in *i*-PrOH (2 mL) at −20 °C, NaBH_4_ (15 mg, 0.4 mmol) was added. The mixture was stirred for 3 h before it was quenched by addition of saturated NH_4_Cl solution and diluted with brine and extracted with CH_2_Cl_2_. The extract was dried over anhydrous Na_2_SO_4_, filtered, and concentrated in vacuo. The residue was purified by silica gel column chromatography (petroleum ether/EtOAc = 6/1) to give the 16-OH product (90 mg, 89%) as a white foam.

To a solution of the above product (49 mg, 0.04 mmol) in MeOH (3 mL), KOH (110 mg, 2 mmol) was added at room temperature. The mixture was stirred at 55 °C for 24 h before it was quenched with acetic acid. The mixture was concentrated under vacuum. The residue was purified by reversed-phase silica gel column chromatography (ODS RP-18) (CHCl_3_/MeOH = 5:1 to 7:1) to give saikosaponin D (**2**) (26 mg, 83%) as a white powder. R*_f_* = 0.6 (silica, CHCl_3_/MeOH = 3:1); [α${]}_{D}^{25}$ = 40.4 (*c* = 1.0, CH_3_OH); ^1^H NMR (500 MHz, pyridine-*d*_5_): *δ* 6.31 (d, *J* = 4.1 Hz, 1H), 6.06 (d, *J* = 10.3 Hz, 1H), 5.73 (d, *J* = 2.8 Hz, 1H), 5.71 (d, *J* = 2.6 Hz, 1H), 5.36 (d, *J* = 7.8 Hz, 1H), 5.00 (d, *J* = 7.8 Hz, 1H), 4.57 (m, 2H), 4.46–4.37 (m, 2H), 4.28 (m, 5H), 4.09–4.02 (m, 3H), 3.73 (d, *J* = 10.8 Hz, 1H), 3.71–3.67 (m, 1H), 3.62 (d, *J* = 3.2 Hz, 1H), 3.60 (d, *J* = 7.0 Hz, 1H), 3.35 (d, *J* = 7.0 Hz, 1H), 2.74–2.64 (m, 1H), 1.65 (s, 3H), 1.46 (s, 3H), 1.45 (s, 3H), 1.38 (s, 3H), 1.04 (s, 3H), 0.98 (s, 3H), 0.96 (s,3H). ^13^C NMR (126 MHz, pyridine-*d*_5_): *δ* 150.59, 150.46, 150.25, 150.03, 136.22, 136.13, 135.93, 135.73, 132.41, 132.35, 124.20, 124.10, 123.90, 123.70, 107.09, 106.40, 85.63, 85.28, 82.03, 79.20, 78.83, 78.20, 77.52, 76.22, 72.55, 72.22, 71.93, 71.42, 64.43, 63.08, 53.42, 51.74, 50.07, 47.75, 45.73, 44.14, 43.99, 42.27, 39.05, 38.76, 37.21, 36.68, 35.85, 34.15, 32.29, 31.93, 31.69, 26.53, 24.80, 19.95, 19.24, 18.50, 17.96, 17.65, 13. ESI-HRMS (*m*/*z*) calcd for C_42_H_69_NaO_13_ [M + H]^+^ 781.4733, found 781.4736.

#### 3.2.16. Synthesis of Saikosaponin Y (**5**)

To a stirred solution of the compound **23** (85 mg, 0.07 mmol) in MeOH (3 mL), KOH (307 mg, 5.5 mmol) was added at room temperature. The mixture was stirred at 55 °C for 24 h before it was quenched with acetic acid. The mixture was concentrated under vacuum. The residue was purified by reversed-phase silica gel column chromatography (ODS RP-18) (CHCl_3_/MeOH = 5:1 to 7:1) to give saikosaponin Y (**5**) (50 mg, 94%) as a white powder. Rf = 0.3 (silica, CHCl_3_/MeOH = 4:1); [α${]}_{D}^{25}$ = 26.6 (c = 1.0, CH_3_OH); ^1^H NMR (500 MHz, CD_3_OD): *δ* 6.02 (d, *J* = 10.3 Hz, 1H), 5.52 (dd, *J* = 10.2, 3.1 Hz, 1H), 4.53 (d, *J* = 7.7 Hz, 1H), 4.37 (d, *J* = 7.4 Hz, 1H), 3.89(d, *J* = 7.4 Hz, 1H), 3.97–3.71 (m, 2H), 3.76–3.53 (m, 7H), 3.43 (d, *J* = 7.9 Hz, 1H), 3.40–3.15 (m, 7H), 2.77 (m, 1H), 2.26 (m, 1H), 2.09 (m, 1H), 1.25 (d, *J* = 6.2 Hz, 3H), 1.19 (s, 3H), 1.00 (s, 3H), 0.96 (s, 3H), 0.91 (s, 3H), 0.89 (s, 3H), 0.70 (s, 3H). ^13^C NMR (125 MHz, CD_3_OD): *δ* 214.74, 134.56, 129.61, 105.65, 85.59, 85.12, 82.97, 77.88, 77.66, 76.29, 75.33, 72.33, 71.82, 71.32, 71.18, 62.35, 57.35, 56.24, 53.73, 50.84, 47.97, 45.39, 44.05, 42.98, 39.98, 39.18, 37.07, 36.64, 33.84, 32.45, 32.03, 26.14, 25.08, 23.52, 20.75, 20.16, 18.88, 18.12, 16.98, 12.80. ESI-HRMS (*m*/*z*) calcd for C_42_H_67_O_13_ [M + H]^+^ 779.4576, found 779.4569.

#### 3.2.17. Synthesis of Compound **24**

To a stirred solution of compound **9** (290 mg, 1.1 mmol) in pyridine (5 mL), benzoyl chloride (0.8 mL, 4.9 mmol) and DMAP (39 mg, 0.3 mmol) were added. The mixture was stirred at room temperature for 8 h before it was quenched with NaHCO_3_ and extracted with EtOAc. The combined organic phases were washed with brine, dried over anhydrous Na_2_SO_4_, filtered, and concentrated under vacuum. The residue was purified by flash column chromatography on silica gel (petroleum ether/EtOAc = 9/1) to give corresponding benzoylated product (625 mg, 99%) as a white solid.

To a stirred solution of the above product (582 mg, 1.0 mmol) in CH_3_CN/H_2_O (8 mL/2 mL), ceric ammonium nitrate (1.2 g, 2.1 mmol) was added. The mixture was stirred at room temperature for 2 h before it was quenched with Na_2_S_2_O_3_ and extracted with EtOAc. The combined organic phases were washed with brine, dried over anhydrous Na_2_SO_4_, filtered, and concentrated under vacuum. The residue was purified by flash column chromatography (petroleum ether/EtOAc = 2:1) to give the lactol (375 mg, 81%) as an orange foam. The α/β anomers were difficult to separate.

The lactol (298 mg, 0.62 mmol), *ortho*-(cyclopropylethynyl) benzoic acid (138 mg, 0.82 mmol), EDCI (156 mg, 0.82 mmol), and DMAP (100 mg, 0.82 mmol) were dissolved in CH_2_Cl_2_ (5 mL). The mixture was stirred at room temperature for 4 h and concentrated under vacuum. The residue was purified by flash chromatography (petroleum ether/EtOAc = 3:1) to give compound **24** (401 mg, 99%; α/β = 1:2.1) as a white foam.

**24α:** R*_f_* = 0.3 (silica, PE/EtOAc = 4:1); [α${]}_{D}^{25}$ = 205.6 (*c* = 1.0, CHCl_3_); ^1^H NMR (500 MHz,CDCl_3_): *δ* 8.14 (d, *J* = 7.3 Hz, 2H), 8.01 (d, *J* = 7.6 Hz, 1H), 7.89 (d, *J* = 7.4 Hz, 2H), 7.82 (d, *J* = 7.4 Hz, 2H), 7.64 (t, *J* = 7.4 Hz, 1H), 7.50 (m, 6H), 7.37 (t, *J* = 7.2 Hz, 1H), 7.28 (m, 4H), 6.94 (d, *J* = 3.6 Hz, 1H), 6.12 (dd, *J* = 10.7, 3.3 Hz, 1H), 6.02 (dd, *J* = 10.7, 3.6 Hz, 1H), 5.93 (d, *J* = 2.7 Hz, 1H), 4.82 (q, *J* = 6.4 Hz, 1H), 1.60 (m, 1H), 1.35 (d, *J* = 6.5 Hz, 3H), 0.95–0.82 (m, 4H). ^13^C NMR (125 MHz, CDCl_3_): *δ* 166.05, 165.76, 164.52, 135.08, 133.67, 133.44, 133.34, 132.41, 130.98, 130.54, 130.03, 129.88, 129.77, 129.26, 129.11, 128.95, 128.76, 128.46, 128.40, 127.41, 125.06, 99.97, 91.16, 74.95, 71.56, 69.31, 68.30, 67.70, 16.38, 9.13, 9.10.

**24β:** R*_f_* = 0.25 (silica, PE/EtOAc = 4:1); [α${]}_{D}^{25}$ = 216.5 (*c* = 1.0, CHCl_3_); ^1^H NMR (500 MHz,CDCl_3_): *δ* 8.16–8.12 (m, 2H), 8.01–7.98 (m, 1H), 7.92–7.88 (m, 2H), 7.81 (dd, *J* = 8.2, 7.1 Hz, 2H), 7.63 (t, *J* = 7.4 Hz, 1H), 7.50 (t, *J* = 7.7 Hz, 2H), 7.47–7.38 (m, 4H), 7.32 (t, *J* = 7.8 Hz, 2H), 7.29–7.24 (m, 3H), 6.26 (d, *J* = 8.4 Hz, 1H), 6.03 (dd, *J* = 10.3, 8.4 Hz, 1H), 5.81 (d, *J* = 3.0 Hz, 1H), 5.70 (dd, *J* = 10.4, 3.5 Hz, 1H), 4.33 (q, *J* = 6.2 Hz, 1H), 1.54 (m, 1H), 1.39 (d, *J* = 6.4 Hz, 3H), 0.90 (m, 4H). ^13^C NMR (125 MHz, CDCl_3_): *δ* 166.07, 165.69, 165.51, 163.75, 134.49, 133.65, 133.48, 133.42, 132.53, 131.08, 130.15, 129.89, 129.85, 129.50, 129.29, 129.04, 128.92, 128.72, 128.51, 128.43, 127.20, 125.84, 100.53, 92.90, 74.44, 72.22, 71.14, 71.01, 68.89, 16.33, 9.15, 9.11. ESI-HRMS (*m*/*z*) calcd for C_39_H_32_NaO_9_ [M + Na]^+^ 667.1339, found 667.1934.

#### 3.2.18. Synthesis of Compound **25**

To a stirred mixture of compound **24** (108 mg, 0.17 mmol), compound **17** (90mg, 0.14 mmol), and 4Å molecular sieves (300 mg) in CH_2_Cl_2_ (3 mL), PPh_3_AuNTf_2_ (21 mg, 0.03 mmol) was added. The mixture was stirred at room temperature for 1 h before it was quenched with NEt_3_. The mixture was filtered through a pad of celite and washed with EtOAc. The filtrate was concentrated under vacuum. The residue was purified by flash column chromatography (petroleum ether/EtOAc = 3:1) to give compound **25** (140 mg, 91%) as a white foam. R*_f_* = 0.3 (silica, PE/EtOAc = 4:1); [α${]}_{D}^{25}$ = 216.5 (*c* = 1.0, CHCl_3_); ^1^H NMR (500 MHz, CDCl_3_): *δ* 8.10 (d, *J* = 7.5 Hz, 2H), 7.97 (d, *J* = 7.5 Hz, 2H), 7.76 (d, *J* = 7.6 Hz, 2H), 7.60 (t, *J* = 7.4 Hz, 1H), 7.49 (m, 3H), 7.43–7.34 (m, 3H), 7.22 (t, *J* = 7.7 Hz, 2H), 5.89 (d, *J* = 10.4 Hz, 1H), 5.76 (dd, *J* = 10.2, 8.2 Hz, 1H), 5.70 (d, *J* = 3.0 Hz, 1H), 5.48 (dd, *J* = 10.5, 3.4 Hz, 1H), 5.42 (dd, *J* = 10.2, 2.2 Hz, 1H), 5.36 (dd, *J* = 9.2, 6.1 Hz, 1H), 4.69 (d, *J* = 7.9 Hz, 1H), 4.04–3.92 (m, 2H), 3.72 (d, *J* = 10.3 Hz, 1H), 3.65 (d, *J* = 11.6 Hz, 1H), 3.54 (dd, *J* = 11.6, 4.6 Hz, 1H), 3.20 (d, *J* = 7.2 Hz, 1H), 1.33 (d, *J* = 6.2 Hz, 3H), 1.24 (s, 9H), 1.19 (s, 9H), 1.05 (s, 3H), 1.00 (s, 3H), 0.96 (s, 3H), 0.90 (s, 3H), 0.88 (s, 3H), 0.57 (s, 3H). ^13^C NMR (125 MHz, CDCl_3_): *δ* 178.27, 177.40, 166.23, 165.83, 165.44, 133.51, 133.39, 133.29, 132.67, 130.17, 129.95, 129.85, 129.71, 129.37, 129.18, 128.97, 128.62, 128.54, 128.36, 103.35, 83.95, 82.76, 73.26, 72.31, 71.17, 69.92, 69.65, 68.27, 64.49, 52.81, 51.70, 47.54, 45.42, 45.26, 42.25, 41.77, 39.12, 39.06, 38.28, 37.42, 35.91, 34.17, 33.48, 31.64, 31.53, 31.04, 29.83, 27.54, 27.32, 25.47, 25.34, 23.78, 20.50, 19.58, 18.08, 17.19, 16.59, 12.07. ESI-HRMS (*m*/*z*) calcd for C_67_H_86_NaO_13_ [M + Na]^+^ 1121.5961 found, 1121.5967.

#### 3.2.19. Synthesis of Compound Prosaikogenin F (**3**)

To a stirred solution of compound **25** (105 mg, 0.1 mmol) in MeOH (3 mL), KOH (272 mg, 5.0 mmol) was added. The mixture was stirred at 55 °C for 24 h before it was quenched with acetic acid. The mixture was concentrated under vacuum. The residue was purified by reversed-phase silica gel column chromatography (ODS RP-18) (CHCl_3_/MeOH = 5:1 to 7:1) to give prosaikogenin F (**3**) (51 mg, 87%) as a white powder. R*_f_* = 0.6 (silica, CHCl_3_/MeOH = 4:1); [α${]}_{D}^{25}$ = 78.6 (c = 1.0, CH_3_OH); ^1^H NMR (500 MHz, pyridine-*d5*): *δ* 6.03 (d, *J* = 10.4 Hz, 1H), 5.69 (dd, *J* = 10.3, 2.9 Hz, 1H), 4.98 (d, *J* = 7.7 Hz, 1H), 4.56–4.50 (m, 1H), 4.44–4.35 (m, 3H), 4.31 (dd, *J* = 11.9, 4.5 Hz, 1H), 4.02 (d, *J* = 6.3 Hz, 2H), 3.82–3.75 (m, 1H), 3.71 (d, *J* = 10.7 Hz, 1H), 3.36 (d, *J* = 6.9 Hz, 1H), 2.51 (d, *J* = 13.3 Hz, 1H), 2.38 (m, 1H), 1.55 (d, *J* = 6.4 Hz, 3H), 1.42 (s, 3H), 1.12 (s, 3H), 1.02 (s, 3H), 0.95 (s, 3H), 0.94 (s, 3H), 0.92 (s, 3H). ^13^C NMR (125 MHz, pyridine-*d*_5_): *δ* 132.68, 131.67, 106.83, 84.48, 82.12, 75.99, 73.52, 73.46, 73.32, 71.77, 64.75, 64.54, 53.59, 52.65, 47.88, 47.50, 46.14, 44.19, 42.71, 39.15, 38.23, 36.81, 36.63, 35.19, 34.15, 32.11, 26.51, 26.26, 24.33, 21.36, 20.57, 19.29, 18.08, 18.00, 13.53. ESI-HRMS (*m*/*z*) calcd for C_67_H_86_NaO_13_ [M + Na]^+^ 641.4024, found 641.4021.

#### 3.2.20. Synthesis of Compound **26**

To a stirred mixture of compound **24** (97 mg, 0.15 mmol), compound **22** (70 mg, 0.13 mmol), and 4Å molecular sieves (300 mg) in CH_2_Cl_2_ (3 mL), PPh_3_AuNTf_2_ (19 mg, 0.03 mmol) was added. The mixture was stirred at room temperature for 1 h before it was quenched with NEt_3_.The mixture was filtered through a pad of celite and washed with EtOAc. The filtrate was concentrated under vacuum. The residue was purified by flash column chromatography (petroleum ether/EtOAc = 5:1) to give compound **26** (106 mg, 84%) as a white foam. R*_f_* = 0.3 (silica, PE/EtOAc = 4:1); [α${]}_{D}^{25}$ = 125.0 (c = 1.0, CHCl_3_); ^1^H NMR (500 MHz, CDCl_3_): *δ* 8.09 (d, *J* = 7.3 Hz, 2H), 7.97 (d, *J* = 7.3 Hz, 2H), 7.76 (d, *J* = 7.4 Hz, 2H), 7.59 (t, *J* = 7.4 Hz, 1H), 7.53–7.43 (m,3H), 7.38 (m, 3H), 7.21 (t, *J* = 7.7 Hz, 2H), 5.97 (d, *J* = 10.4 Hz, 1H), 5.77 (dd, *J* = 10.4, 8.0 Hz, 1H), 5.69 (d, *J* = 3.3 Hz, 1H), 5.54 (dd, *J* = 10.3, 2.7 Hz, 1H), 5.49 (dd, *J* = 10.5, 3.4 Hz, 1H), 4.71 (d, *J* = 7.9 Hz, 1H), 4.00 (dd, *J* = 12.7, 6.2 Hz, 1H), 3.86 (d, *J* = 7.9 Hz, 1H), 3.73 (d, *J* = 11.5 Hz, 1H), 3.66 (d, *J* = 11.6 Hz, 1H), 3.55 (dd, *J* = 11.5, 4.6 Hz, 1H), 3.44 (d, *J* = 8.0 Hz, 1H), 2.68 (m, 1H), 1.33 (d, *J* = 6.3 Hz, 3H), 1.22 (s, 9H), 1.15 (s, 3H), 0.95 (s, 3H), 0.93 (s, 3H), 0.91 (s, 3H), 0.86 (s, 3H), 0.59 (s, 3H). ^13^C NMR (125 MHz, CDCl_3_): *δ* 212.98, 177.29, 166.18, 165.78, 165.40, 133.47, 133.34, 133.25, 133.15, 130.12, 129.90, 129.80, 129.38, 129.20, 128.97, 128.79, 128.59, 128.50, 128.32, 103.28, 84.14, 82.61, 75.52, 72.28, 71.17, 69.96, 69.64, 64.51, 56.27, 54.99, 52.70, 49.57, 47.63, 44.38, 42.25, 41.70, 39.07, 38.87, 38.26, 35.91, 35.49, 33.36, 31.61, 31.00, 27.47, 25.43, 24.25, 23.23, 20.06, 19.49, 17.96, 17.17, 16.56, 12.04. ESI-HRMS (*m*/*z*) calcd for C_62_H_76_NaO_12_ [M + Na]^+^ 1035.5229, found 1035.5219.

#### 3.2.21. Synthesis of Prosaikogenin G (**4**)

To a stirred solution of compound **26** (90 mg, 0.09 mmol) in *i*-PrOH (3 mL) at −20 °C, NaBH_4_ (7 mg, 0.18 mmol) was added. The mixture was stirred for 3 h, the solution was quenched by addition of saturated NH_4_Cl solution and diluted with brine and extracted with CH_2_Cl_2_. The extract was dried over anhydrous Na_2_SO_4_, filtered, and concentrated in vacuo. The residue was purified by silica gel column chromatography (petroleum ether/EtOAc = 8:1) to give the 16-OH product (86 mg, 95%) as a white foam.

To a stirred solution of the above product (70 mg, 0.07 mmol) in MeOH (3 mL), KOH (193 mg, 3.5 mmol) was added at room temperature. The mixture was stirred at 55 °C for 24 h before it was quenched with acetic acid. The mixture concentrated under vacuum. The residue was purified by reversed-phase silica gel column chromatography (ODS RP-18) (CHCl_3_/MeOH = 5:1) to give prosaikogenin G (**4**) (35 mg, 83%) as a white powder. R*_f_* = 0.6 (silica, CHCl_3_/MeOH = 4:1); [α${]}_{D}^{25}$ = 54.1 (*c* = 1.0, CH_3_OH); ^1^H NMR (500 MHz, pyridine-*d*_5_): *δ* 6.30 (d, *J* = 4.0 Hz, 1H), 6.07 (d, *J* = 10.4 Hz, 1H), 5.72 (dd, *J* = 10.2, 2.6 Hz, 1H), 4.99 (d, *J* = 7.7 Hz, 1H), 4.38 (t, *J* = 8.4 Hz, 2H), 4.32 (dd, *J* = 11.8, 4.5 Hz, 1H), 3.79 (q, *J* = 6.3 Hz, 1H), 3.72 (d, *J* = 9.5 Hz, 1H), 3.60(d, *J* = 7.0 Hz, 1H), 3.35 (d, *J* = 7.0 Hz, 1H), 2.72–2.65 (m, 1H), 2.53 (m, 1H), 2.40 (m, 1H), 1.64 (s, 3H), 1.56 (d, *J* = 6.4 Hz, 3H), 1.38 (s, 3H), 1.06 (s, 3H), 1.04 (s, 3H), 0.98 (s, 3H), 0.96 (s, 3H). ^13^C NMR (125 MHz, pyridine-*d*_5_): *δ* 132.52, 132.45, 106.80, 85.38, 82.20, 78.30, 77.61, 75.99, 73.47, 73.32, 71.77, 64.84, 53.52, 51.84, 47.94, 45.83, 44.20, 44.09, 42.38, 39.14, 38.87, 37.31, 36.82, 35.94, 34.25, 32.39, 32.04, 31.79, 26.52, 24.90, 20.06, 19.37, 18.60, 18.08, 17.97, 13.55. ESI-HRMS (*m*/*z*) calcd for C_36_H_58_NaO_8_ [M + Na]^+^ 641.4024, found 641.4050.

#### 3.2.22. Synthesis of Prosaikogenin (**6**)

To a stirred solution of the compound **26** (100 mg, 0.1 mmol) in MeOH (3 mL), KOH (360 mg, 5 mmol) was added at room temperature. The mixture was stirred at 55 °C for 24 h before it was quenched with acetic acid. The mixture was concentrated under vacuum. The residue was purified by reversed-phase silica gel column chromatography (ODS RP-18) (MeOH/H_2_O = 5:1) to give compound prosaikogenin (**6**) (57 mg, 93%) as a white powder. R*_f_* = 0.6 (silica, MeOH/H_2_O = 4:1); [α${]}_{D}^{25}$ = 13.3 (*c* = 1.0, MeOH); ^1^H NMR (500 MHz, pyridine-*d*_5_): *δ* 6.06 (t, *J* = 17.8 Hz, 1H), 5.72 (dd, *J* = 10.3, 2.8 Hz, 1H), 4.99 (d, *J* = 7.7 Hz, 1H), 4.43–4.35 (m, 1H), 4.32 (dd, *J* = 11.9, 4.6 Hz, 1H), 4.08–3.99 (m, 1H), 3.94 (d, *J* = 7.8 Hz, 1H), 3.78 (q, *J* = 6.4 Hz, 1H), 3.71 (d, *J* = 10.8 Hz, 1H), 3.53 (d, *J* = 7.9 Hz, 1H), 2.85 (d, *J* = 14.4 Hz, 1H), 1.55 (d, *J* = 6.4 Hz, 1H), 1.39 (s, 1H), 1.03 (s, 1H), 1.00 (s, 1H), 0.93 (s, 1H), 0.87 (s, 1H), 0.82 (s, 1H). ^13^C NMR (125 MHz, pyridine-*d*_5_): *δ* 212.79, 133.70, 129.96, 106.87, 84.74, 81.88, 75.90, 73.38, 73.25, 71.75, 64.55, 56.85, 55.57, 53.27, 50.27, 47.74, 45.25, 44.16, 42.57, 39.44, 39.00, 36.72, 36.36, 33.80, 32.13, 31.82, 26.49, 24.98, 23.60, 20.71, 20.29, 19.13, 18.01, 17.90, 13.52. ESI-HRMS (*m*/*z*) calcd for C_42_H_67_O_13_ [M]^+^ 639.3867, found 639.3869.

#### 3.2.23. Synthesis of Compound **27**

To a stirred solution of the compound **10** (225 mg, 0.4 mmol) in MeOH (3 mL), CH_3_ONa (12 mg, 0.2 mmol) was added. The mixture was stirred at room temperature for 8 h before it was quenched with acid resin until pH = 7. The mixture was filtered through a pad of celite and washed with MeOH. The filtrate was concentrated under vacuum.

To a stirred mixture of the above product in pyridine (5 mL), triphenylmethyl chloride (1.04 g, 3.8 mmol) and DMAP (45 mg, 0.4 mmol) were added. The mixture was stirred for 8 h at 75 °C, then benzoyl chloride (0.43 mL, 3.8 mmol) was added in the flask at 0 °C. The mixture was stirred for 4 h at room temperature, the solution was quenched by addition of saturated NaHCO_3_ solution, diluted with brine and extracted with CH_2_Cl_2_. The combined organic phases were dried over anhydrous Na_2_SO_4_, filtered, and concentrated in vacuo. The residue was purified by silica gel column chromatography (petroleum ether/EtOAc = 5:1) to give compound **27** (431 mg, 97%) as a white foam. R*_f_* = 0.3 (silica, PE/EtOAc = 4:1); [α${]}_{D}^{25}$ = 50.0 (*c* = 1.0, CHCl_3_); ^1^H NMR (500 MHz, CDCl_3_): *δ* 8.19 (d, *J* = 8.0 Hz, 2H), 8.14 (d, *J* = 7.4 Hz, 1H), 7.99 (d, *J* = 8.1 Hz, 2H), 7.73 (t, *J* = 6.2 Hz, 3H), 7.63 (m, 2H), 7.57–7.46 (m, 11H), 7.43–7.28 (m, 6H), 7.25–7.19 (m, 4H), 7.14 (m, 3H), 7.01–6.92 (m, 4H), 6.81 (d, *J* = 8.4 Hz, 2H), 5.99 (s, 1H), 5.80 (d, *J* = 3.4 Hz, 1H), 5.72 (m, 1H), 5.54 (m, 2H), 5.43 (m, 1H), 5.20 (d, *J* = 7.6 Hz, 1H), 4.96–4.88 (m, 1H), 4.48 (d, *J* = 6.0 Hz, 1H), 3.96–3.90 (m, 1H), 3.76 (s, 3H), 3.62–3.54 (m, 1H), 3.30 (d, *J* = 10.5 Hz, 1H), 1.23 (d, *J* = 6.0 Hz, 3H). ^13^C NMR (125 MHz, CDCl_3_): *δ* 166.30, 165.85, 165.60, 164.97, 164.63, 155.40, 151.01, 143.74, 133.71, 133.53, 133.21, 133.17, 133.09, 132.62, 130.26, 130.23, 130.06, 129.80, 129.77, 129.74, 129.38, 129.28, 129.10, 128.99, 128.89, 128.70, 128.66, 128.56, 128.47, 128.30, 128.27, 128.00, 127.97, 127.13, 118.54, 114.72, 102.05, 96.66, 87.00, 74.24, 74.13, 73.26, 73.23, 72.06, 71.17, 70.04, 66.39, 62.90, 55.72, 16.50. ESI-HRMS (*m*/*z*) calcd for C_73_H_62_NaO_16_ [M + Na]^+^ 1217.3930, found 1217.3938.

#### 3.2.24. Synthesis of Compound **29**

To a stirred solution of compound **27** (420 mg, 0.4 mmol), **28** (438 mg, 0.7 mmol), and 4Å molecular sieves (400 mg) in CH_2_Cl_2_ (4 mL), TfOH (62 μL, 0.7 mmol) was added at −20 °C. The mixture was stirred at the same temperature for 1.5 h before it was quenched with NEt_3_ (5.0 mL). The mixture was filtered through a pad of celite and washed with CH_2_Cl_2_ (3 × 2 mL). The filtrate was concentrated under vacuum. The residue was purified by flash column chromatography (petroleum ether/EtOAc = 3:1) to give compound **29** (400 mg, 74%) as an orange foam. R*_f_* = 0.3 (silica, PE/EtOAc = 4:1); [α${]}_{D}^{25}$ = 21.2 (*c* = 0.25, CHCl_3_); ^1^H NMR (500 MHz, CDCl_3_): *δ* 8.17 (d, *J* = 8.0 Hz, 2H), 8.14–8.07 (m, 4H), 7.89 (d, *J* = 7.9 Hz, 2H), 7.83 (m, 4H), 7.78 (d, *J* = 7.9 Hz, 2H), 7.66–7.61 (m, 3H), 7.61–7.28 (m, 23H), 7.20 (t, *J* = 7.5 Hz, 1H), 7.16 (t, *J* = 7.8 Hz, 2H), 7.04 (d, *J* = 8.9 Hz, 2H), 6.87 (t, *J* = 7.7 Hz, 2H), 6.77 (d, *J* = 9.0 Hz, 2H), 6.00–5.90 (m, 2H), 5.82 (d, *J* = 3.6 Hz, 1H), 5.74 (t, *J* = 9.5 Hz, 1H), 5.66 (t, *J* = 9.8 Hz, 1H), 5.57 (dd, *J* = 11.6, 6.1 Hz, 1H), 5.48–5.42 (m, 2H), 5.24 (m, 2H), 4.89 (d, *J* = 6.1 Hz, 1H), 4.83 (d, *J* = 7.7 Hz, 1H), 4.73 (m, 2H), 4.58–4.47 (m, 2H), 4.09 (m, 1H), 4.00 (m, 1H), 3.94 (d, *J* = 8.1 Hz, 1H), 3.72 (s, 3H), 1.39 (d, *J* = 6.2 Hz, 3H). ^13^C NMR (125 MHz, CDCl_3_): *δ* 166.68, 166.30, 165.88, 165.74, 165.52, 165.40, 165.32, 165.20, 164.58, 155.65, 150.88, 133.60, 133.55, 133.51, 133.41, 133.32, 133.24, 133.20, 132.64, 130.35, 129.99, 129.92, 129.86, 129.80, 129.67, 129.40, 129.27, 129.03, 128.86, 128.80, 128.68, 128.65, 128.59, 128.56, 128.52, 128.47, 128.42, 128.26, 127.93, 119.74, 114.71, 102.25, 101.31, 97.14, 75.51, 75.12, 73.91, 73.00, 72.50, 72.34, 71.66, 71.16, 69.41, 69.31, 65.85, 63.14, 55.66, 16.66. ESI-HRMS (*m*/*z*) calcd for C_88_H_74_NaO_25_ [M + Na]^+^ 1553.4411, found 1553.4409.

#### 3.2.25. Synthesis of Compound **30**

To a stirred solution of compound **29** (350 mg, 0.2 mmol) in CH_3_CN/H_2_O (2 mL/0.5 mL), ceric ammonium nitrate (275 mg, 0.5 mmol) was added. The mixture was stirred at 0 °C for 2 h before it was quenched with NaHCO_3_ and extracted with EtOAc. The combined organic phases were washed with brine, dried over anhydrous Na_2_SO_4_, filtered, and concentrated under vacuum. The residue was purified by flash column chromatography (petroleum ether/EtOAc = 2:1) to give the lactol (266 mg, 81%) as an orange foam. The α/β anomers were difficult to separate.

The lactol (230 mg, 0.16 mmol), *ortho*-(cyclopropylethynyl) benzoic acid (42 mg, 0.24 mmol), EDCI (44 mg, 0.2 mmol) and DMAP (6 mg, 0.05 mmol) were dissolved in CH_2_Cl_2_ (5 mL). The mixture was stirred at room temperature for 4 h and concentrated under vacuum. The residue was purified by flash chromatography (petroleum/EtOAc = 3:1) to give compound **30** (250 mg, 99%) as a white foam. R*_f_* = 0.3 (silica, PE/EtOAc = 2:1); The α/β anomers were difficult to separate.

#### 3.2.26. Synthesis of Compound **31**

To a stirred mixture of compound **30** (220 mg, 0.14 mmol), compound **17** (68 mg, 0.1 mmol), and 4Å molecular sieves (300 mg) in CH_2_Cl_2_ (3 mL), PPh_3_AuNTf_2_ (16 mg, 0.03 mmol) was added. The mixture was stirred at 0 °C for 1 h before it was quenched with NEt_3_. The mixture was filtered through a pad of celite and washed with EtOAc. The filtrate was concentrated under vacuum. The residue was purified by flash column chromatography (petroleum ether/EtOAc = 4:1) to give compound **31** (171 mg, 79%) as a white foam. R*_f_* = 0.3 (silica, PE/EtOAc = 4:1); [α${]}_{D}^{25}$ = 36.3 (*c* = 1, CHCl_3_); ^1^H NMR (500 MHz, CDCl_3_): *δ* 8.14 (d, *J* = 7.4 Hz, 2H), 8.05 (m, 4H), 7.89 (d, *J* = 7.6 Hz, 2H), 7.85 (d, *J* = 7.5 Hz, 2H), 7.81 (d, *J* = 7.5 Hz, 2H), 7.68–7.51 (m, 9H), 7.44 (m, 10H), 7.38–7.27 (m, 10H), 7.13 (t, *J* = 7.8 Hz, 2H), 7.01 (t, *J* = 7.7 Hz, 2H), 5.92 (d, *J* = 9.6 Hz, 1H), 5.90–5.84 (m, 1H), 5.68 (m, 2H), 5.53 (t, *J* = 9.7 Hz, 1H), 5.47 (m, 2H), 5.42–5.38 (m, 1H), 5.35 (d, *J* = 7.9 Hz, 2H), 5.14 (td, *J* = 9.9, 4.5 Hz, 2H), 4.69–4.61 (m, 2H), 4.54–4.46 (m, 2H), 4.45–4.40 (m, 1H), 4.08 (dd, *J* = 9.9, 3.7 Hz, 1H), 3.96 (d, *J* = 6.9 Hz, 4H), 3.56 (d, *J* = 11.5 Hz, 1H), 3.46 (m, 2H), 3.19 (d, *J* = 7.2 Hz, 1H), 1.37 (d, *J* = 6.2 Hz, 3H), 1.25 (s, 3H), 1.18 (s, 9H), 1.10 (s, 9H), 1.03 (s, 3H), 0.97 (s, 3H), 0.94 (s, 3H), 0.87 (s, 3H), 0.85 (s, 3H). ^13^C NMR (125 MHz, CDCl_3_): *δ* 178.28, 177.34, 166.86, 166.28, 165.96, 165.68, 165.32, 165.23, 164.61, 164.46, 133.70, 133.56, 133.23, 132.72, 130.49, 129.96, 129.91, 129.85, 129.75, 129.65, 129.53, 129.27, 128.94, 128.90, 128.73, 128.68, 128.56, 128.52, 128.45, 128.34, 128.22, 127.96, 102.90, 101.81, 101.15, 83.94, 81.91, 78.18, 75.11, 73.41, 73.11, 72.71, 72.39, 72.31, 71.87, 71.59, 69.64, 69.47, 69.38, 68.30, 63.13, 52.78, 51.70, 47.46, 45.42, 45.27, 42.13, 41.75, 39.06, 38.99, 37.43, 35.85, 34.18, 33.47, 31.64, 31.52, 30.96, 29.83, 27.48, 27.32, 25.34, 23.77, 20.48, 19.56, 17.93, 17.22, 17.03, 12.01. ESI-HRMS (*m*/*z*) calcd for C_121_H_130_NaO_29_ [M + Na]^+^ 2069.8595, found 2069.8590.

#### 3.2.27. Synthesis of Clinoposaponin I (**7**)

To a stirred solution of the compound **31** (51mg, 0.02 mmol) in MeOH (3 mL), KOH (111 mg, 2.0 mmol) was added. The mixture was stirred at 55 °C for 24 h before it was quenched with acetic acid. The mixture concentrated under vacuum. The residue was purified by reversed-phase silica gel column chromatography (ODS RP-18) (MeOH/H_2_O = 2:1) to give compound **7** (21 mg, 89%) as a white powder. R*_f_* = 0.5 (reverse silica, MeOH/H_2_O = 2:1); [α${]}_{D}^{25}$ = 28.5 (*c* = 0.25, CH_3_OH); ^1^H NMR (500 MHz, pyridine-*d*_5_): *δ* 6.01 (m, 1H), 5.69 (dd, *J* = 10.3, 2.8 Hz, 1H), 5.28 (d, *J* = 7.8 Hz, 1H), 5.09 (d, *J* = 7.7 Hz, 3H), 5.00 (d, *J* = 7.8 Hz, 1H), 4.86 (d, *J* = 10.9 Hz, 1H), 4.53 (m, 1H), 4.45–4.37 (m, 3H), 4.28 (dt, *J* = 11.9, 5.9 Hz, 3H), 4.22 (t, *J* = 8.0 Hz, 2H), 4.19–3.98 (m, 5H), 3.89 (dd, *J* = 11.7, 5.1 Hz, 2H), 3.72 (d, *J* = 10.6 Hz, 1H), 3.37 (d, *J* = 6.9 Hz, 1H), 2.52 (m, 1H), 2.32 (d, *J* = 9.8 Hz, 1H), 1.52 (d, *J* = 6.3 Hz, 3H), 1.41 (s, 3H), 1.12 (s, 3H), 1.00 (s, 3H), 0.94 (s, 3H), 0.93 (s, 3H), 0.91 (s, 3H). ^13^C NMR (125 MHz, pyridine-*d*_5_): *δ* 132.68, 131.67, 106.73, 106.59, 105.98, 85.42, 84.47, 82.12, 78.88, 77.81, 75.99, 75.79, 73.52, 72.59, 72.18, 71.97, 71.52, 64.52, 63.06, 53.59, 52.62, 50.18, 47.82, 47.49, 46.11, 44.20, 42.68, 39.14, 38.20, 36.75, 34.13, 32.10, 26.26, 24.31, 21.34, 20.56, 19.24, 17.82, 13.53. ESI-HRMS (*m*/*z*) calcd for C_48_H_78_NaO_18_ [M + Na]^+^ 965.5080, found 965.5077.

## 4. Conclusions

Here, we report the facile synthesis of seven saikosaponins; namely, saikosaponin A (**1**), D (**2**), prosaikosaponin F (**3**), G (**4**), saikosaponin Y (**5**), prosaikogenin (**6**), and clinoposaponin I (**7**), which have been identified from *Radix Bupleuri*, a common traditional Chinese medicine and relevant plants. These mono-, di-, and trisaccharide saponins feature triterpene aglycones of high oxidation states, which bear 13,28-epoxy-ether moiety and oxo groups at C16 and C23. The methods previously developed for the selective hydroxylation of the triterpenes of low oxidation states, such as oleanolic acid, have enabled the present synthesis. In addition, the gold(I)-catalyzed glycosylation with *o*-alkynylbenzoates as donors has been applied successfully in the present synthesis. Given the conserved nature of the structures of saikosaponins, the work reported herein offers the prospect of being able to access many more members of saikosaponins and their natural and synthetic analogs, thus facilitating in-depth studies on biological and pharmacological activities of these components of folk medicines.

## Data Availability

The data presented in this study are available in the article and Supplementary Material.

## Supplementary Materials

The following are available online at https://www.mdpi.com/article/10.3390/molecules26071941/s1. Copies of NMR spectra of compounds **1**–**7**, **10**–**12**, **14**–**27**, **29** and **31**.

## Sample Availability

Samples of the compounds **1**–**7** are available from the authors.
