# Supplementary material for: Facile Synthesis of Saikosaponins
Source: Molecules. 2021 Mar 30;26(7):1941. doi: 10.3390/molecules26071941 (PMC8037928; doi:10.3390/molecules26071941)
Supplement: Supplementary file 1 [file molecules-26-01941-s001.pdf]

Facile Synthesis of Saikosaponins

Ziqiang Wang [1], Bingcheng Wei [2], Tong Mu [2], Peng Xu [2,3,*] and Biao Yu [2,3,*]

[1] Department of Chemistry, University of Science and Technology of China, 96 Jinzhai Road, Hefei 230026, China
[2] State Key Laboratory of Bio-organic and Natural Products Chemistry, Center for Excellence in Molecular Synthesis, Shanghai Institute of Organic Chemistry, Chinese Academy of Sciences, 345 Lingling Road, Shanghai 200032, China
[3] School of Chemistry and Materials Science, Hangzhou Institute for Advanced Study, University of Chinese Academy of Sciences, 1 Sub-lane Xiangshan, Hangzhou 310024, China
*Correspondence: peterxu@sioc.ac.cn (PX) & byu@sioc.ac.cn (BY)

Table of Contents

Copies of ^{1}H NMR and ^{13}C NMR spectra of compounds **1-7**, **10-12**, **14-27**, **29** and **31**…………………2

Comparison of ^{13}C NMR data of the synthetic **1-5** and **7** with those reported for the natural products…..31

^{1}H NMR of Saikosaponin A (**1**) (500 MHz, pyridine-d_5)

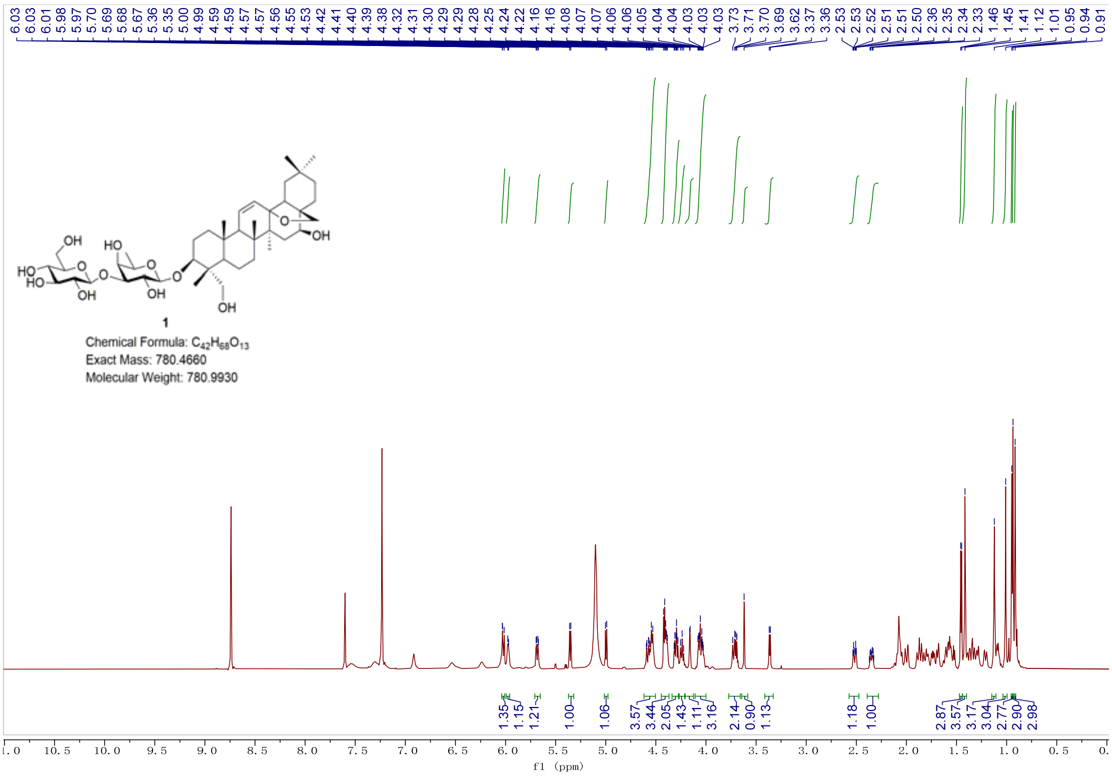

^{13}C NMR of Saikosaponin A (**1**) (125 MHz, pyridine-d_5)

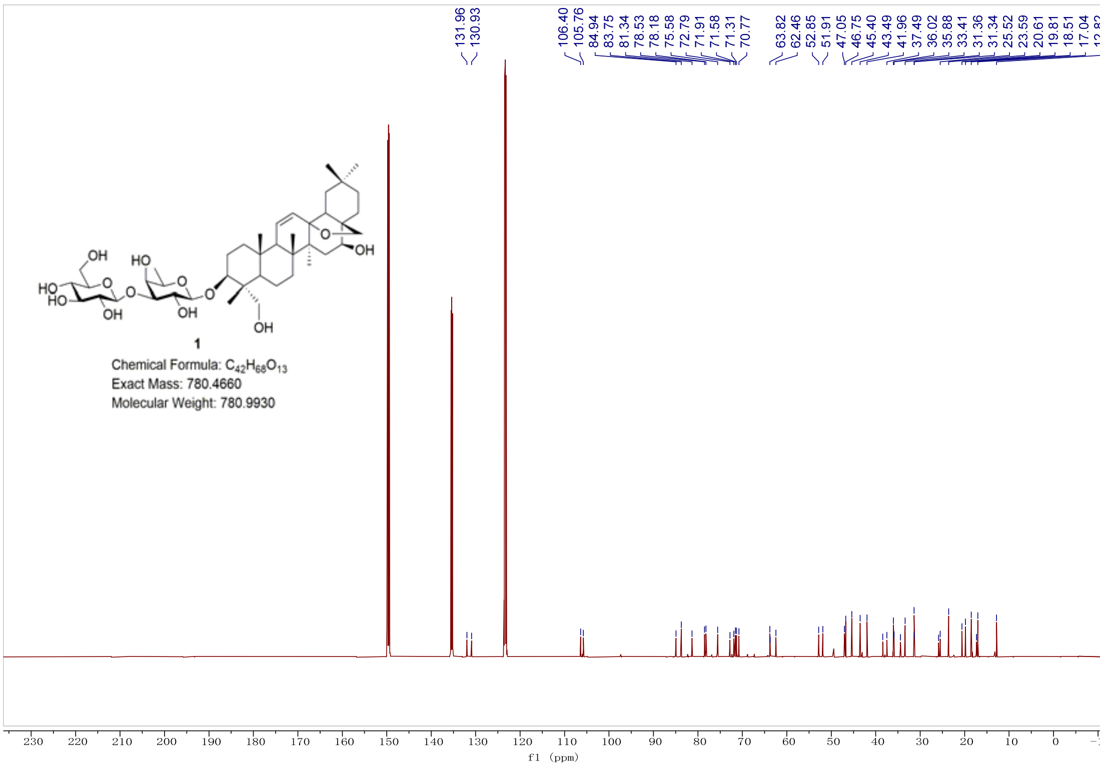

^{1}H NMR of Saikosaponin D (**2**) (500 MHz, pyridine-d_5)

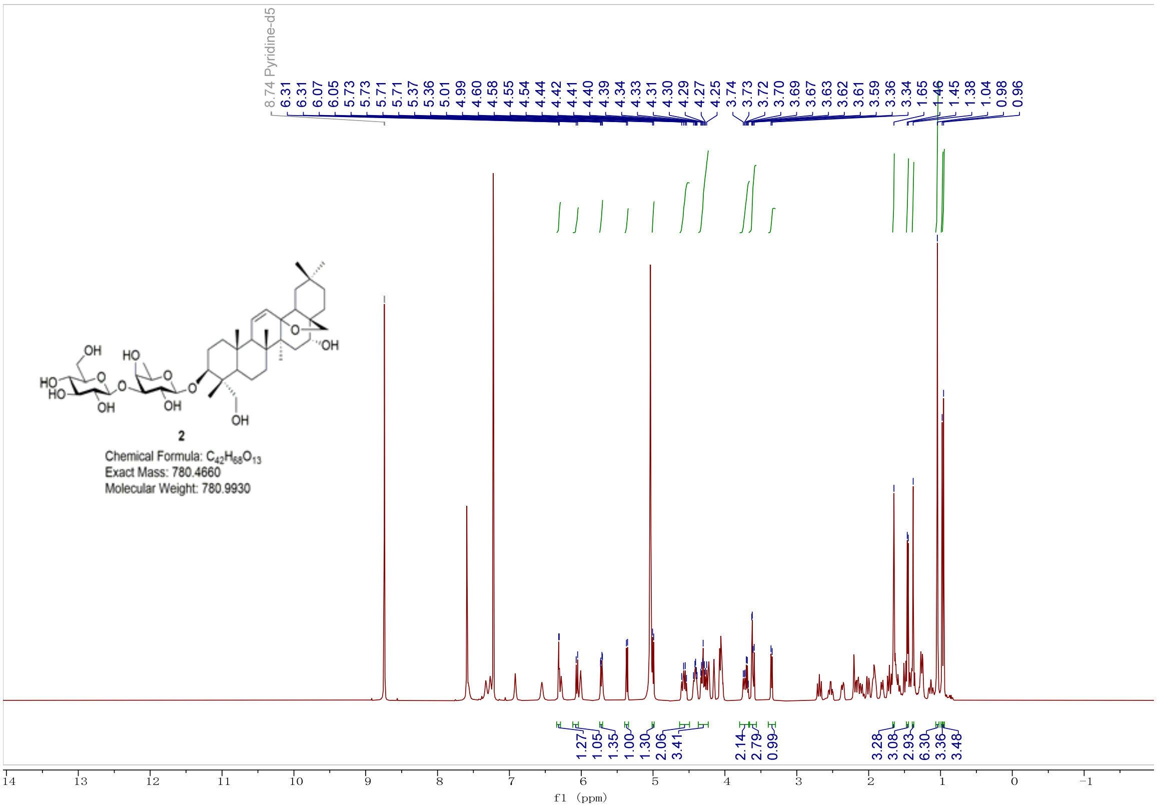

^{13}C NMR of Saikosaponin D (**2**) (125 MHz, pyridine-d_5)

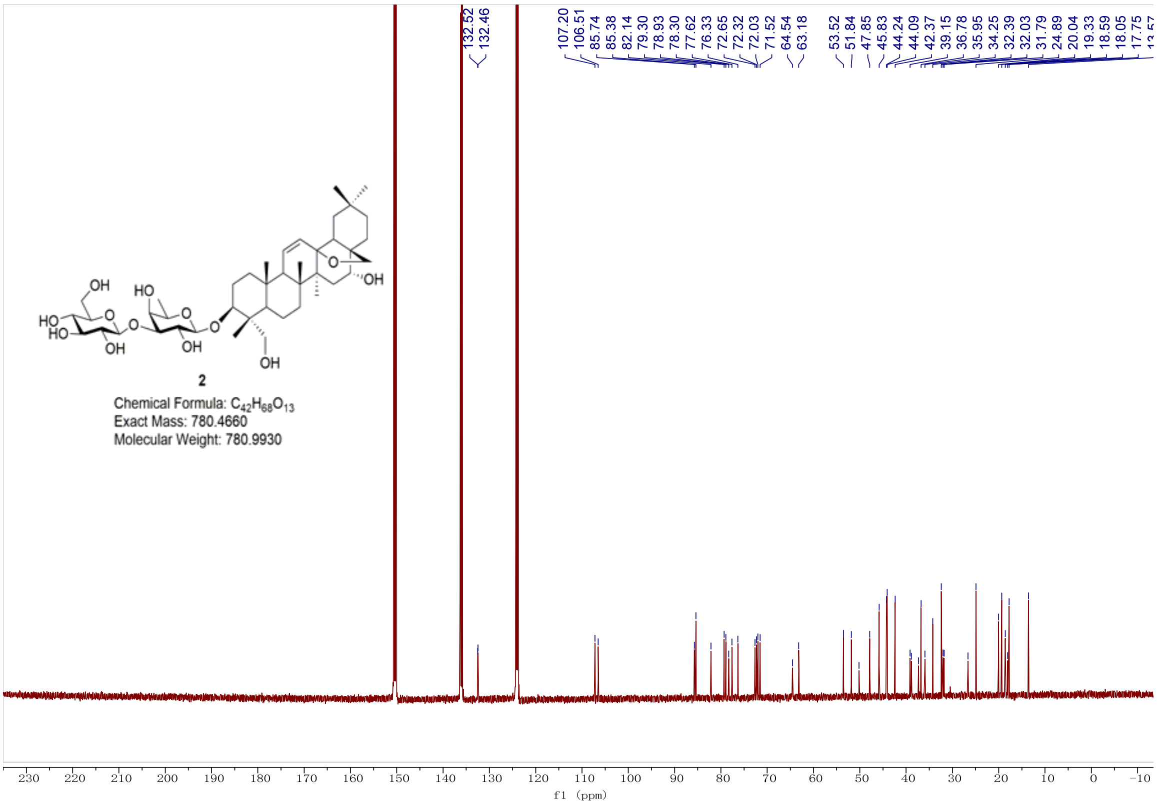

^{1}H NMR of Prosaikogenin F (**3**) (500 MHz, pyridine-d_5)

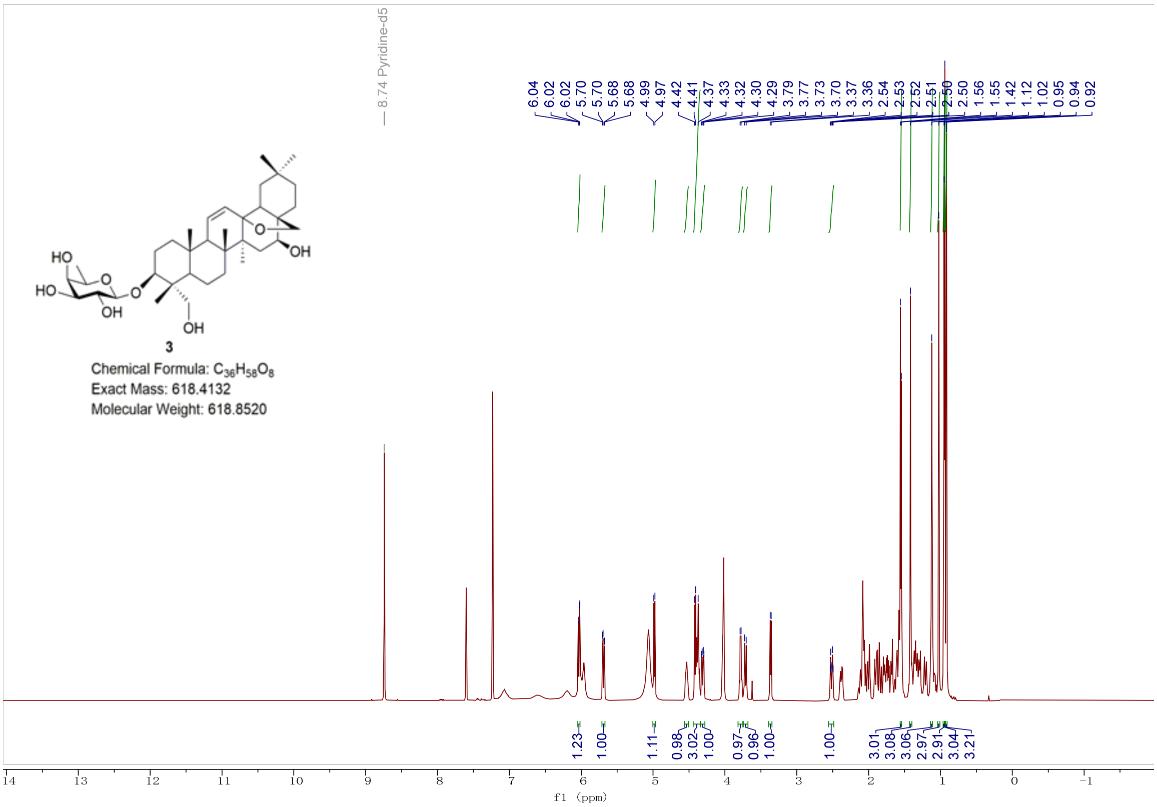

^{13}C NMR of Prosaikogenin F (**3**) (125 MHz, pyridine-d_5)

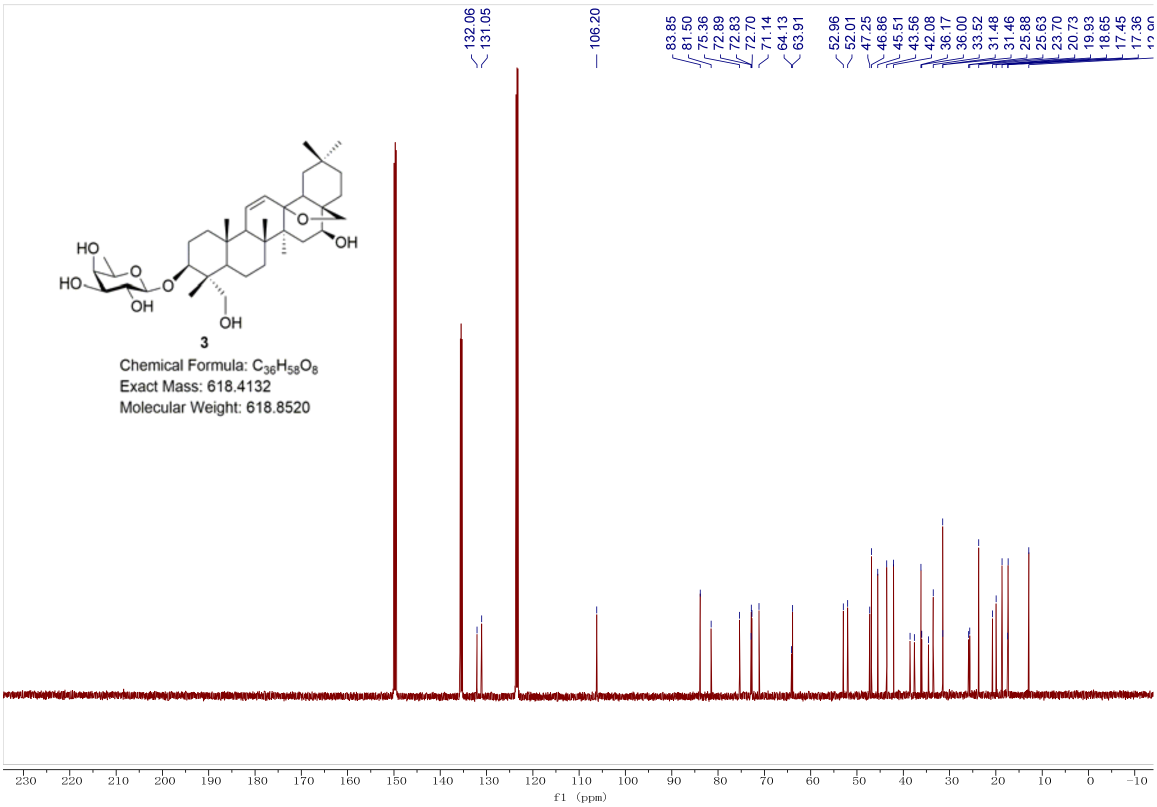

^{1}H NMR of Prosaikogenin G (**4**) (500 MHz, pyridine-d_5)

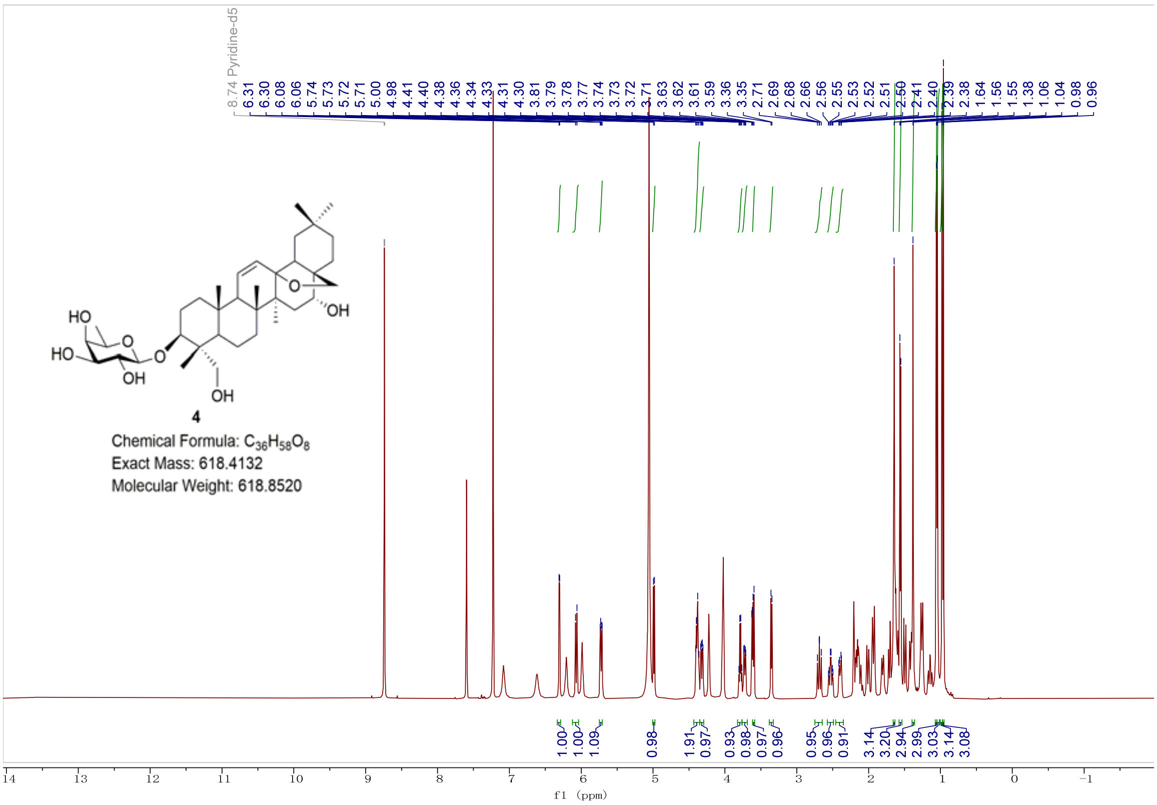

^{13}C NMR of Prosaikogenin G (**4**) (125 MHz, pyridine-d_5)

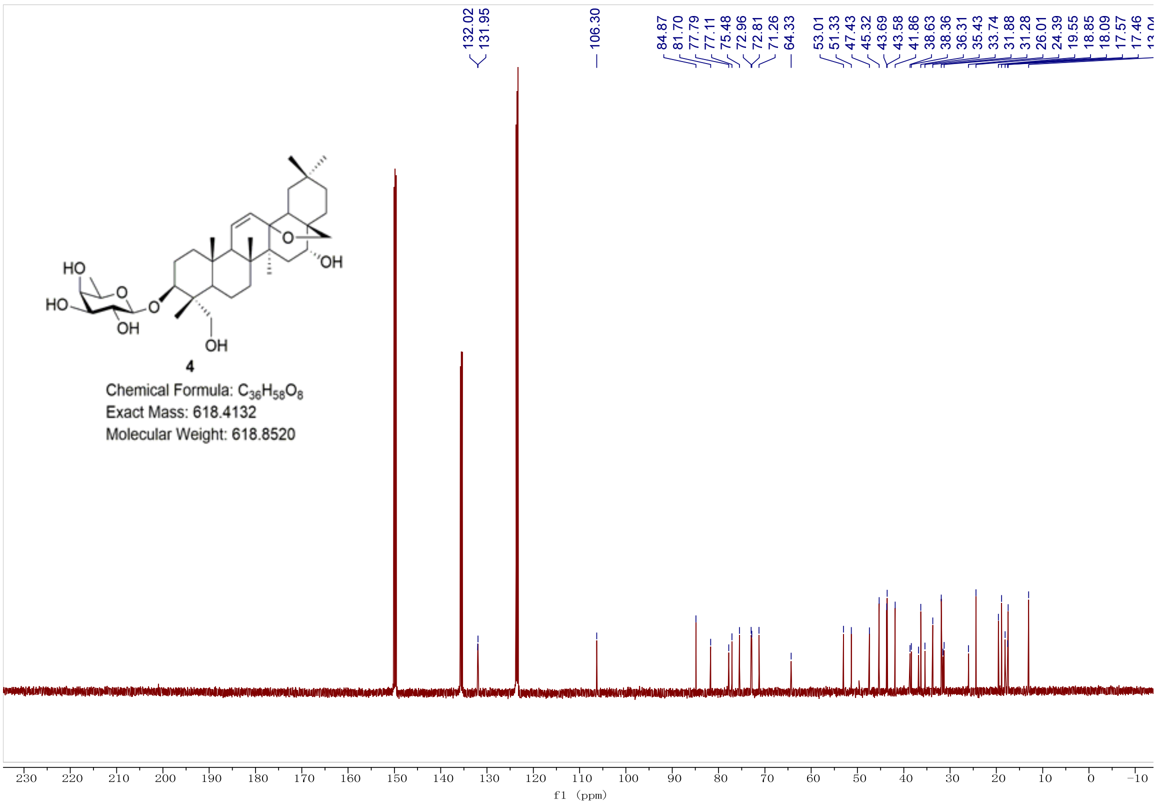

^{1}H NMR of Saikogenin **5** (500 MHz, methanol-*d*4)

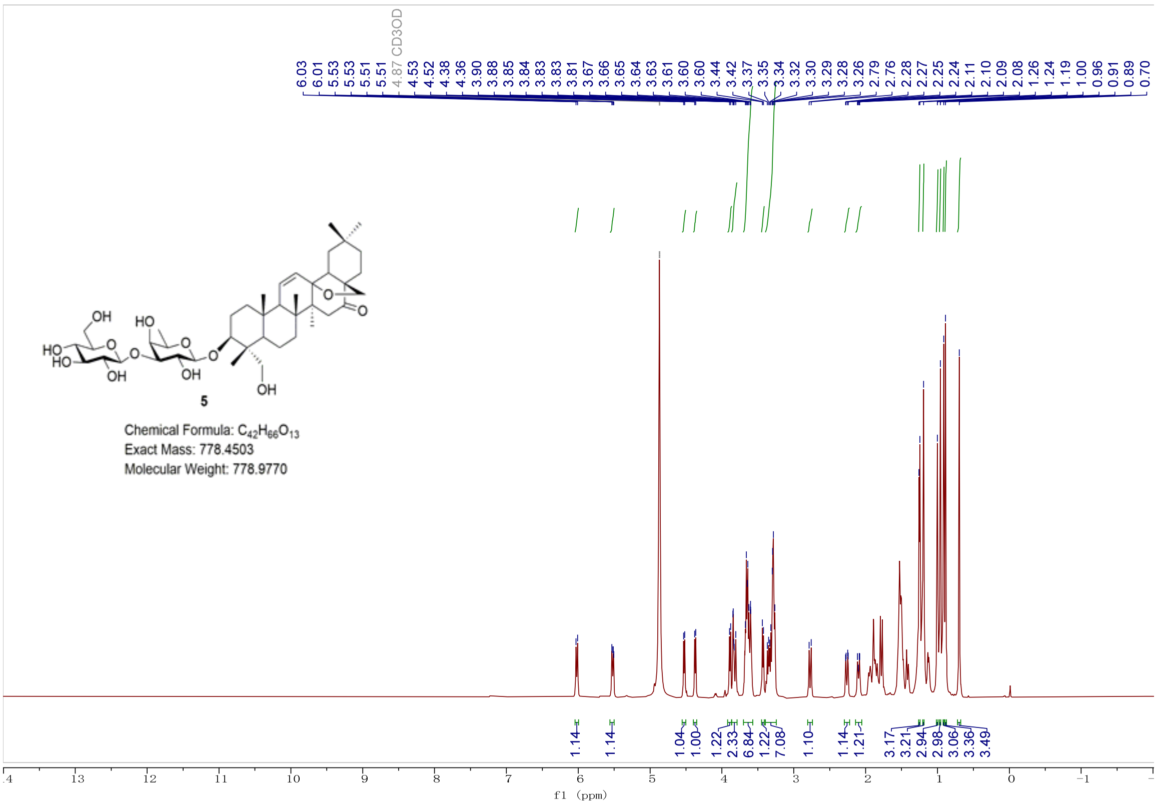

^{13}C NMR of Saikogenin **5** (125 MHz, methanol-*d*4)

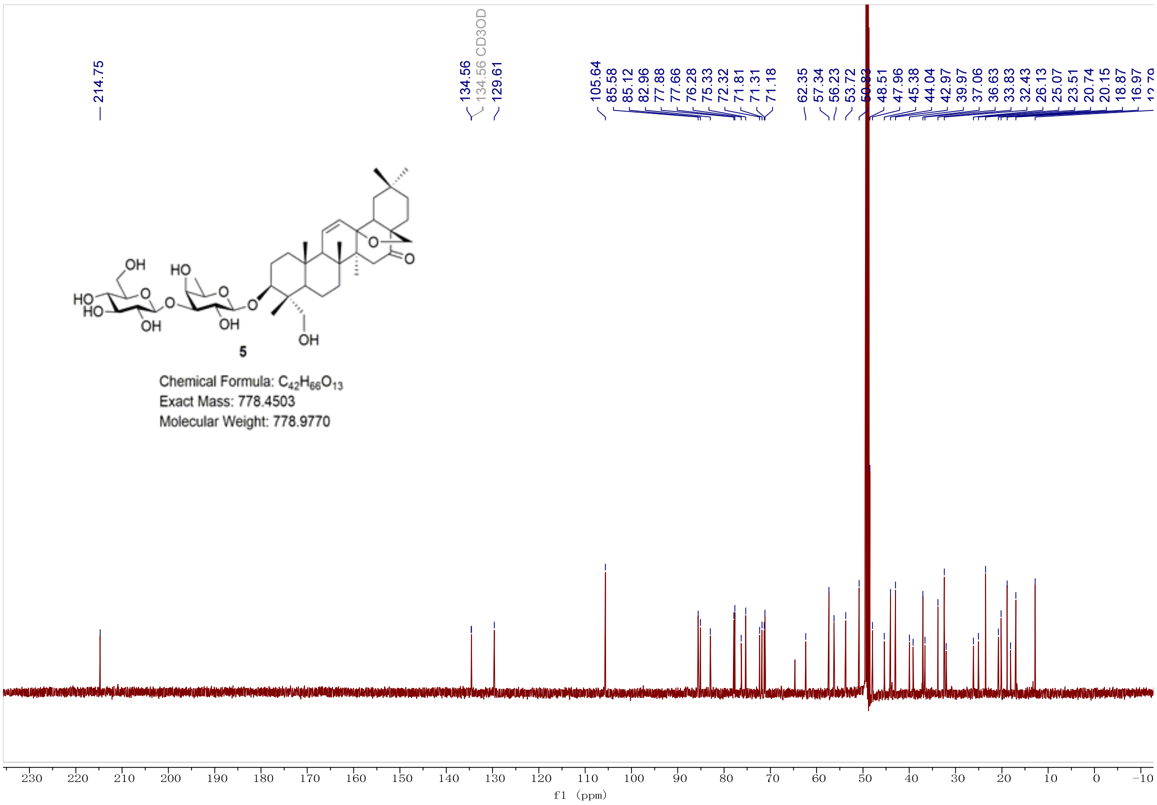

^{1}H NMR of Prosaikogenin **6** (500 MHz, Pyridine-d_5)

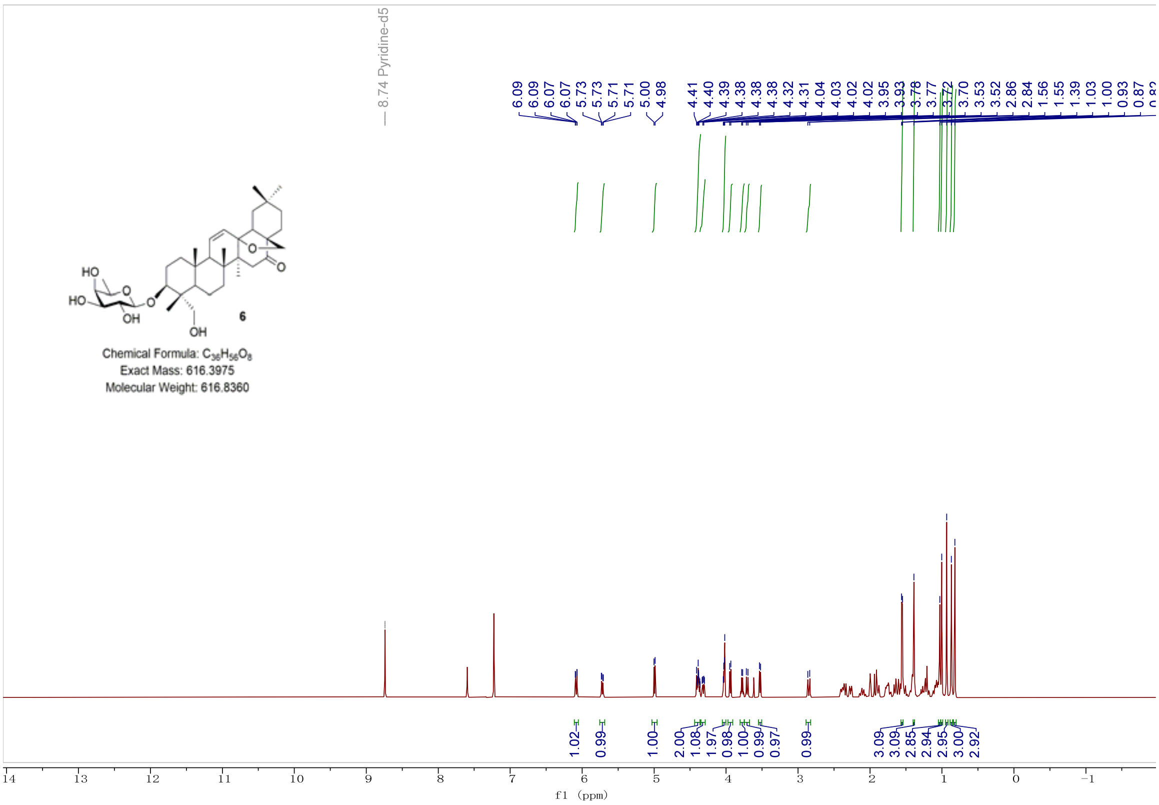

^{13}C NMR of Prosaikogenin **6** (125 MHz, Pyridine-d_5)

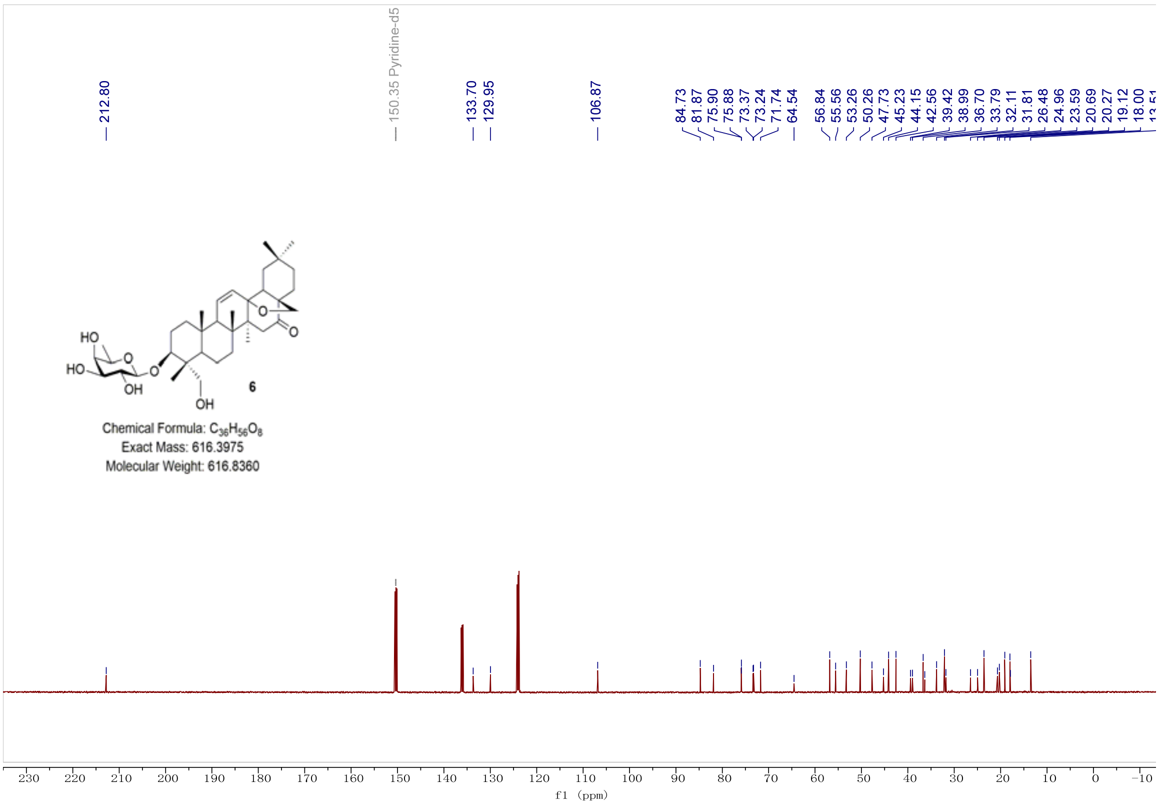

^{1}H NMR of Clinoposaponin I (**7**) (500 MHz, Pyridine-d_5)

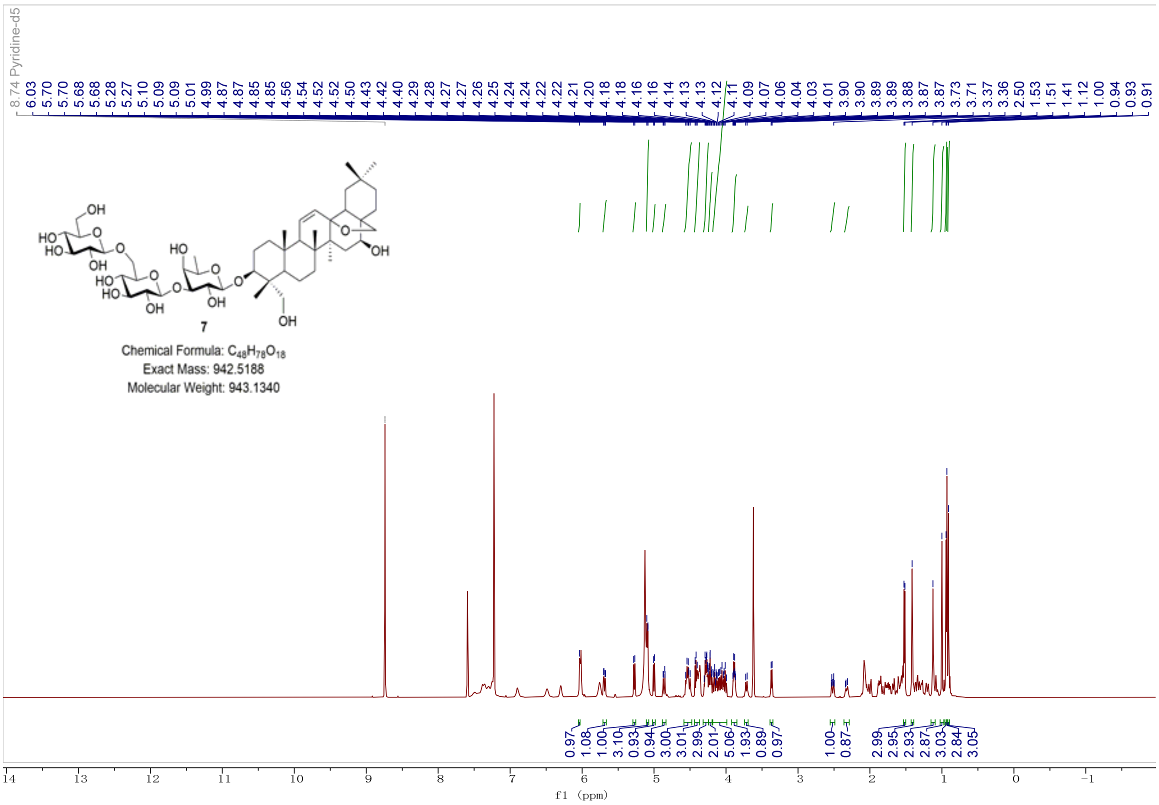

^{13}C NMR of Clinoposaponin I (**7**) (125 MHz, Pyridine-d_5)

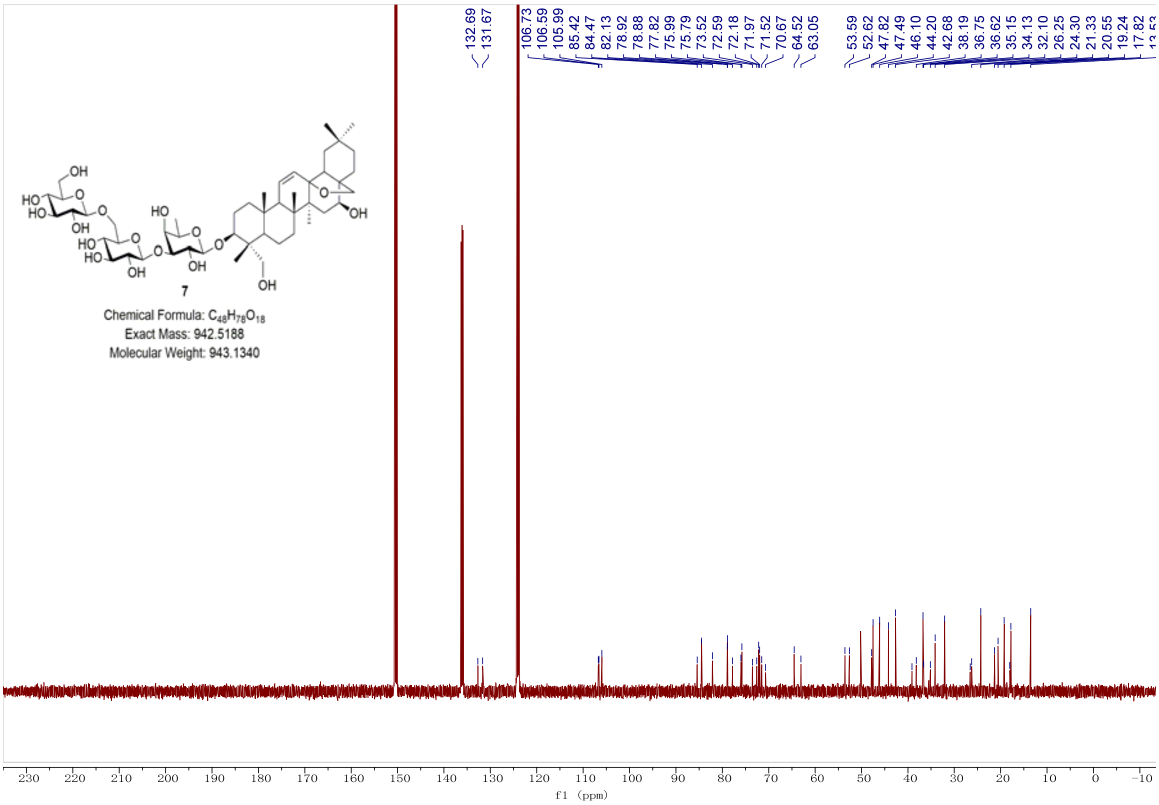

^{1}H NMR of Compound **10** (500 MHz, $CDCl_3$)

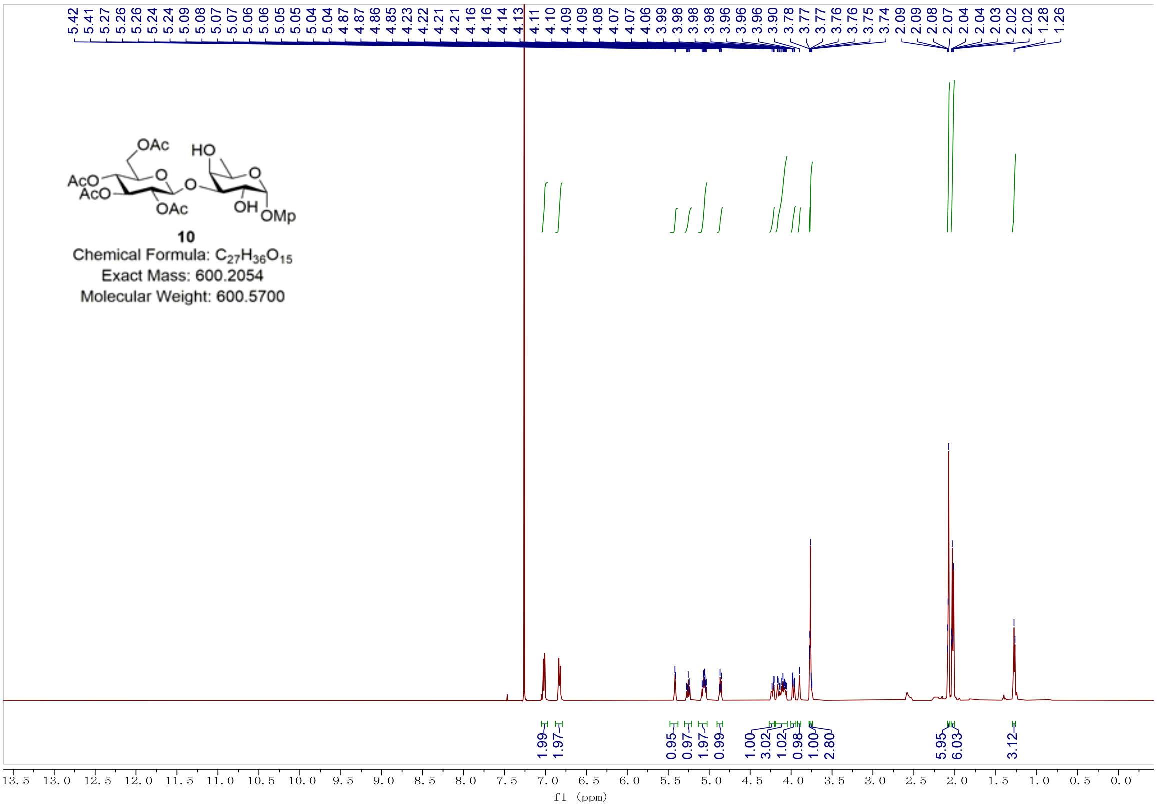

^{13}C NMR of Compound **10** (125 MHz, $CDCl_3$)

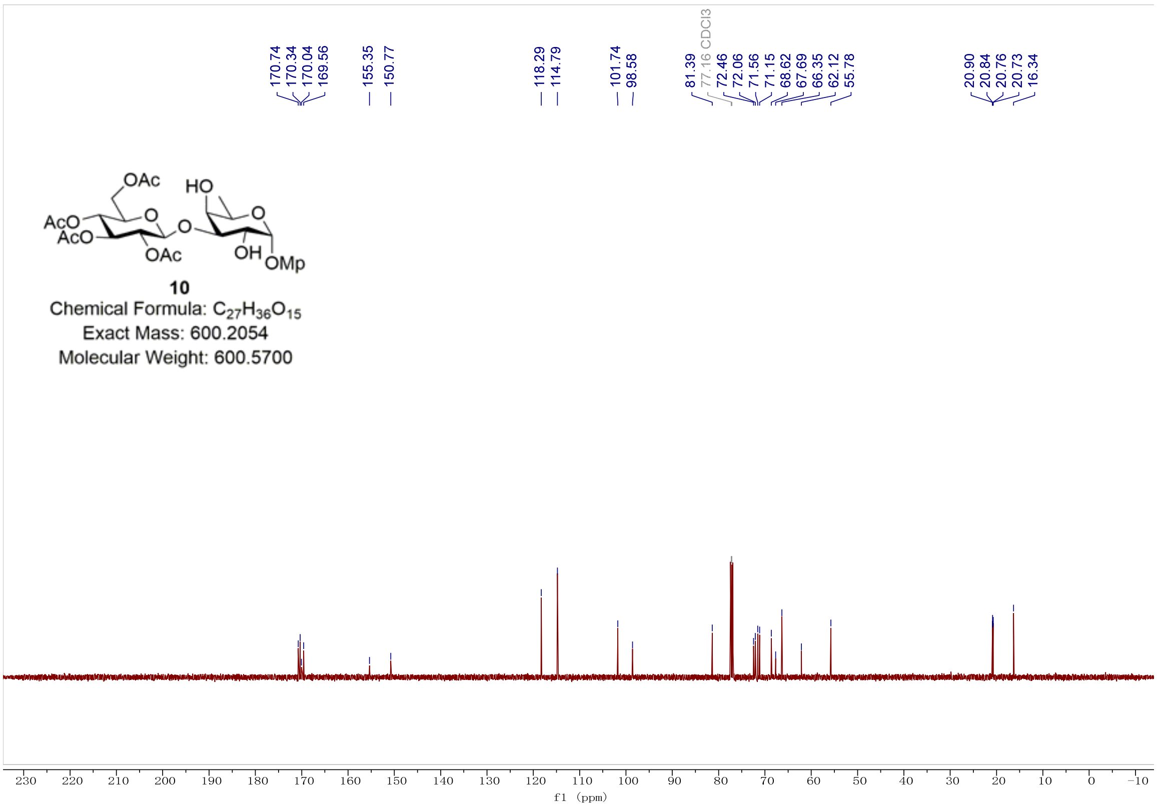

^{1}H NMR of Compound **11α** (500 MHz, $CDCl_3$)

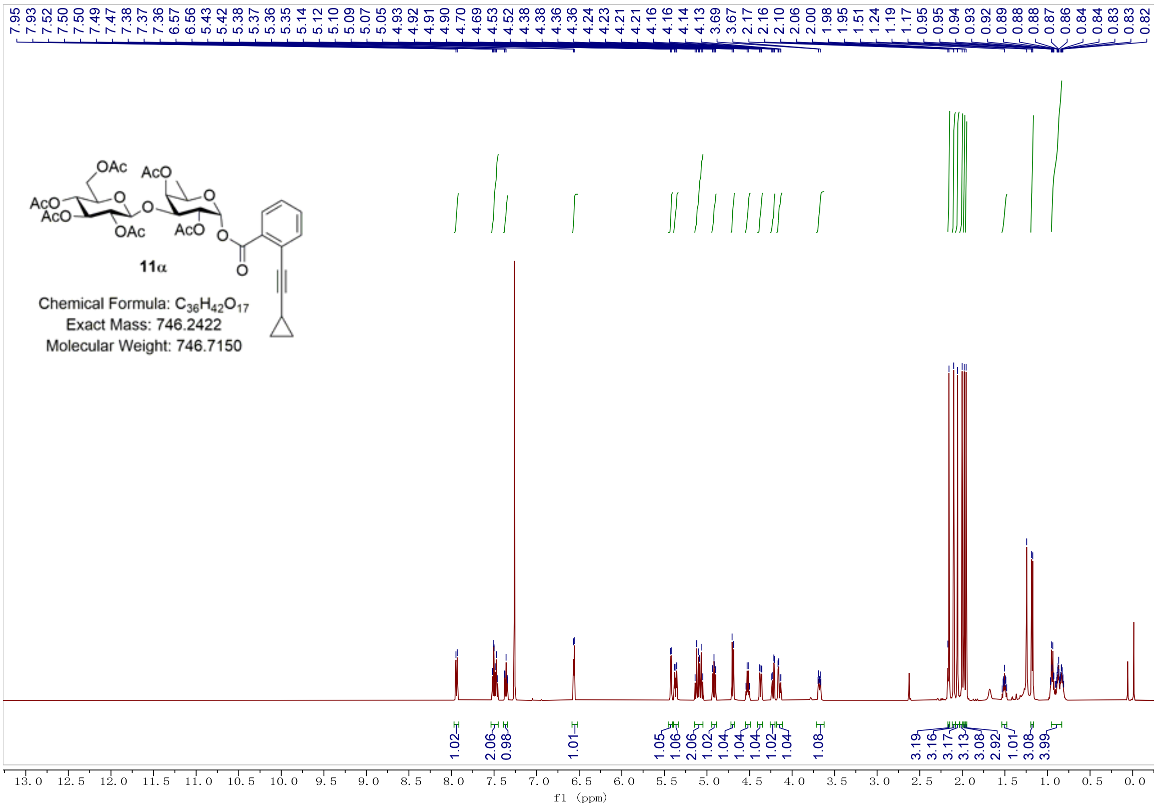

^{13}C NMR of Compound **11α** (125 MHz, $CDCl_3$)

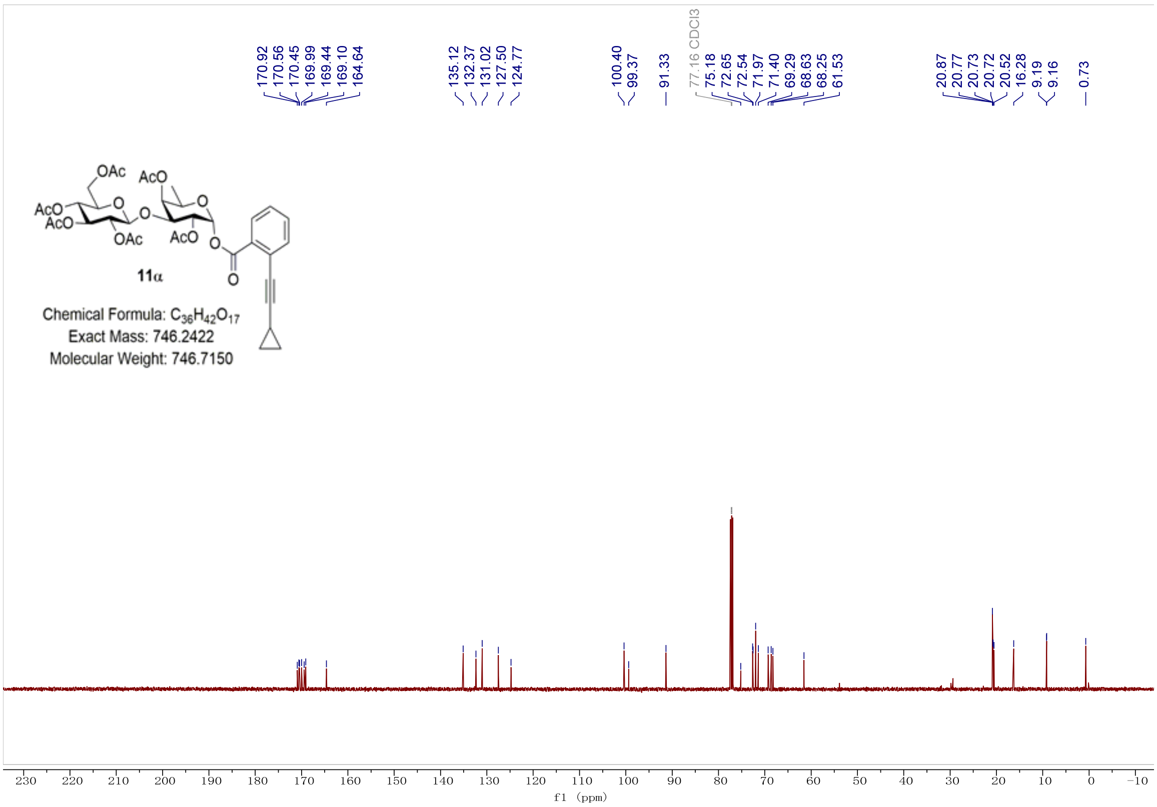

^{1}H NMR of Compound **11β** (500 MHz, $CDCl_3$)

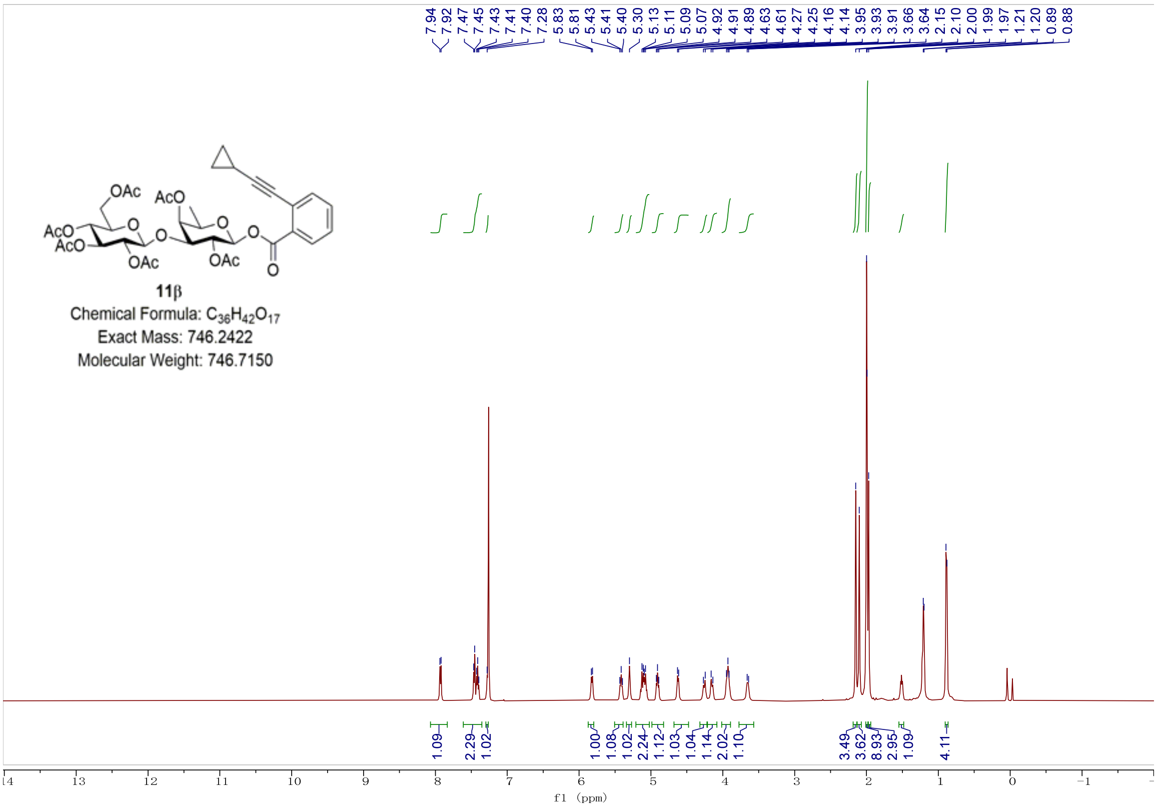

^{13}C NMR of Compound **11β** (125 MHz, $CDCl_3$)

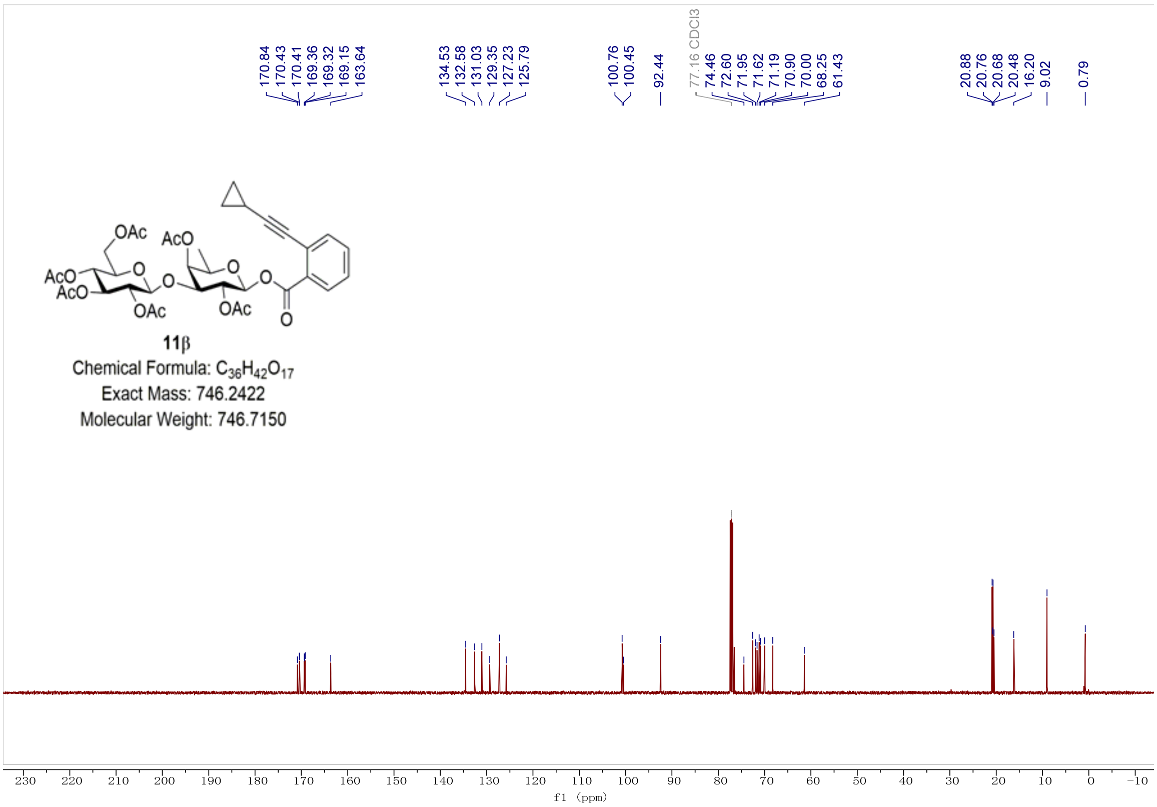

^{1}H NMR of Compound **12α** (500 MHz, $CDCl_3$)

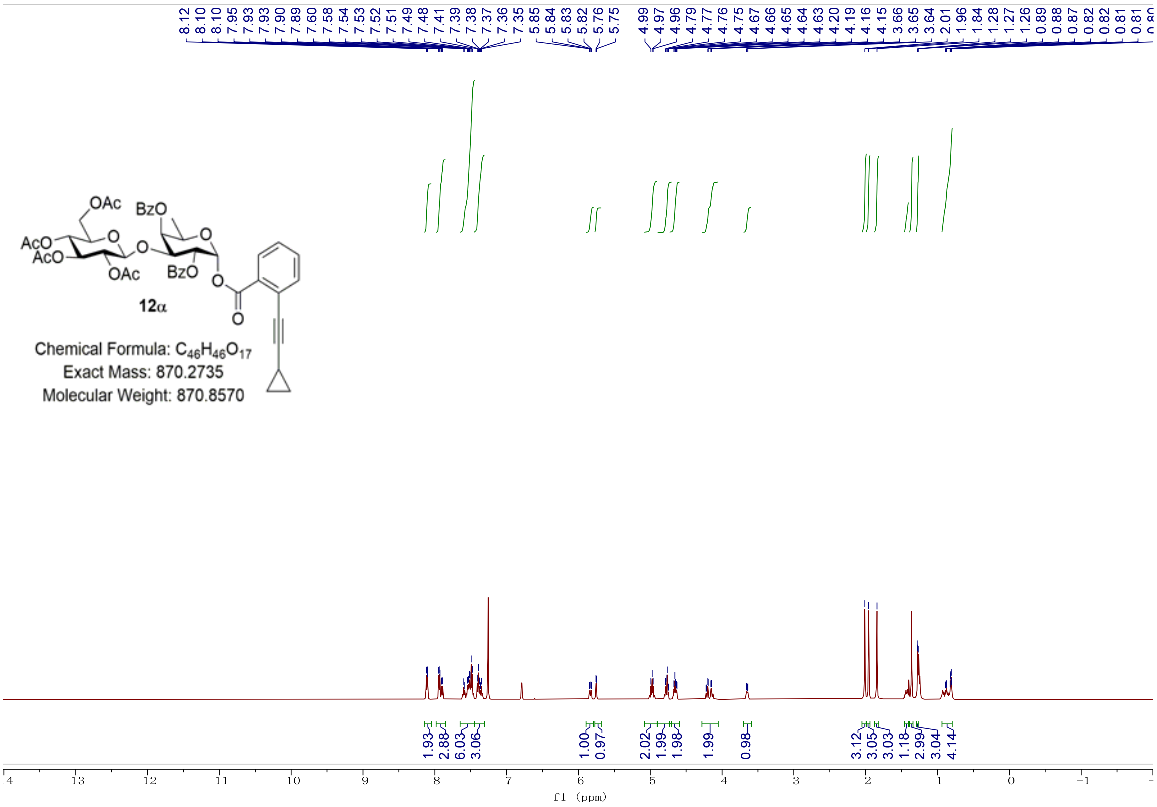

^{13}C NMR of Compound **12α** (125 MHz, $CDCl_3$)

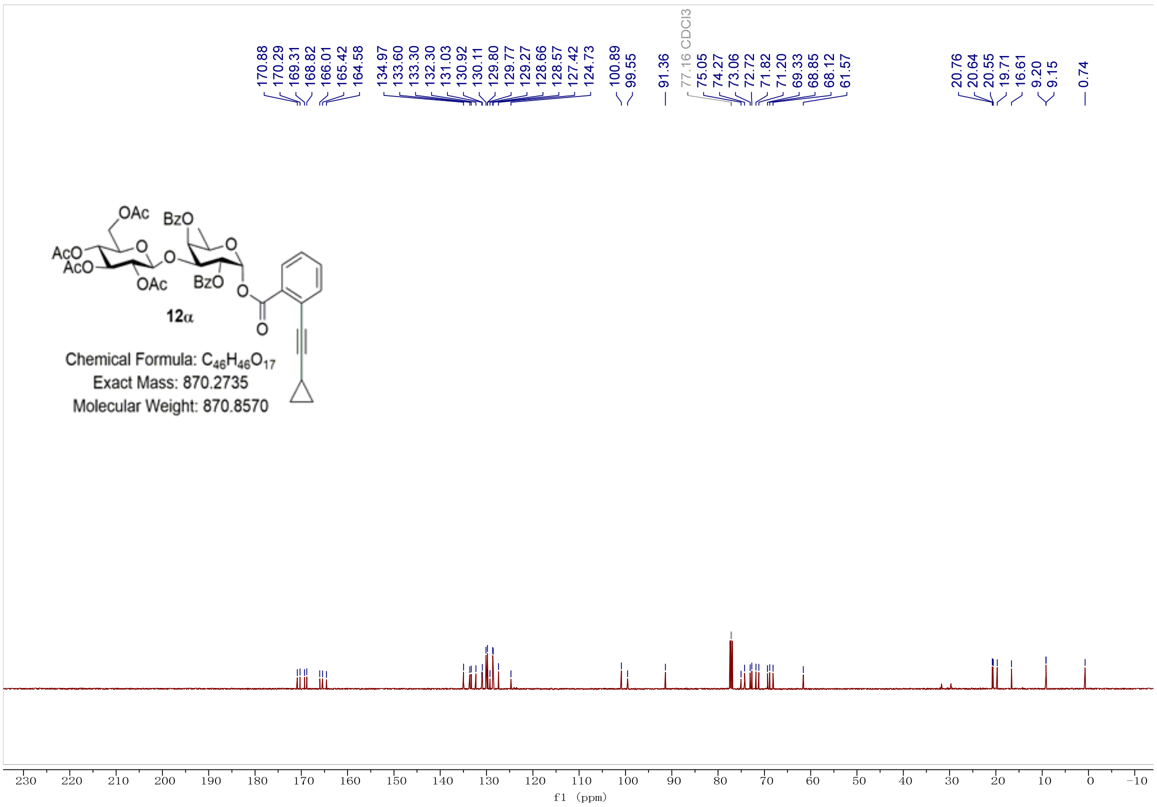

^{1}H NMR of Compound **12β** (500 MHz, $CDCl_3$)

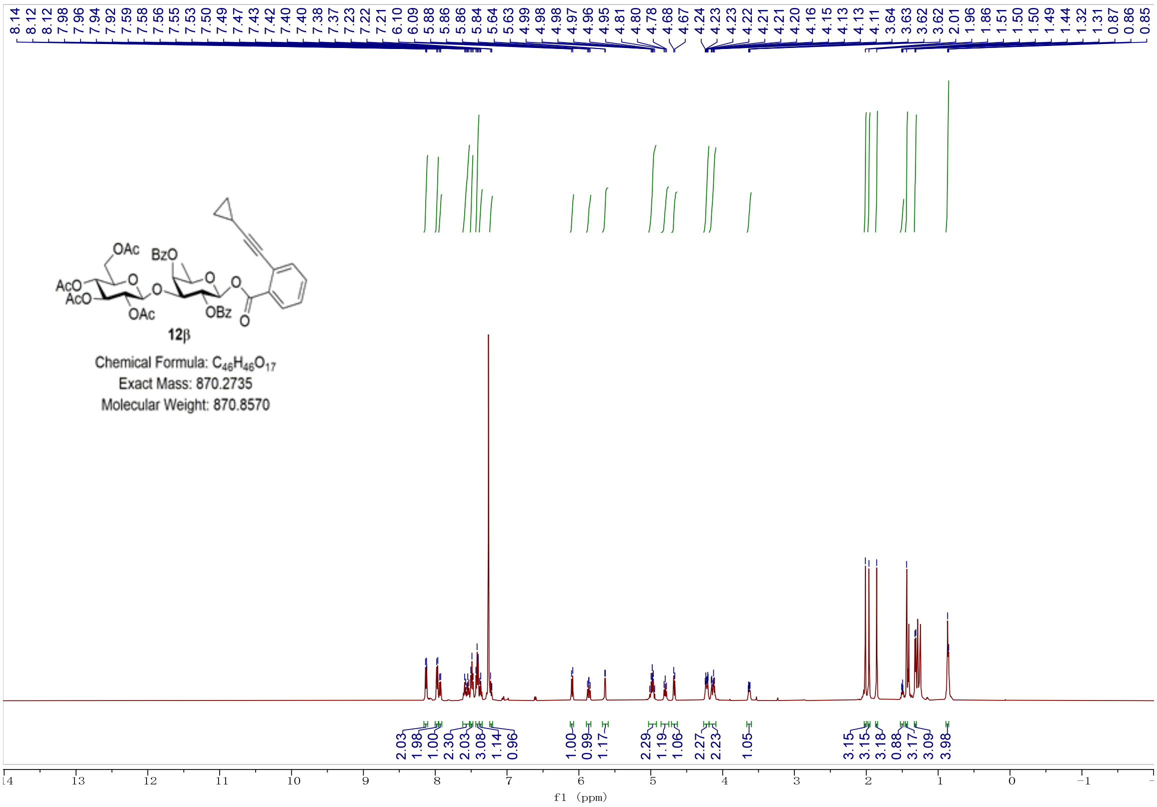

^{13}C NMR of Compound **12β** (125 MHz, $CDCl_3$)

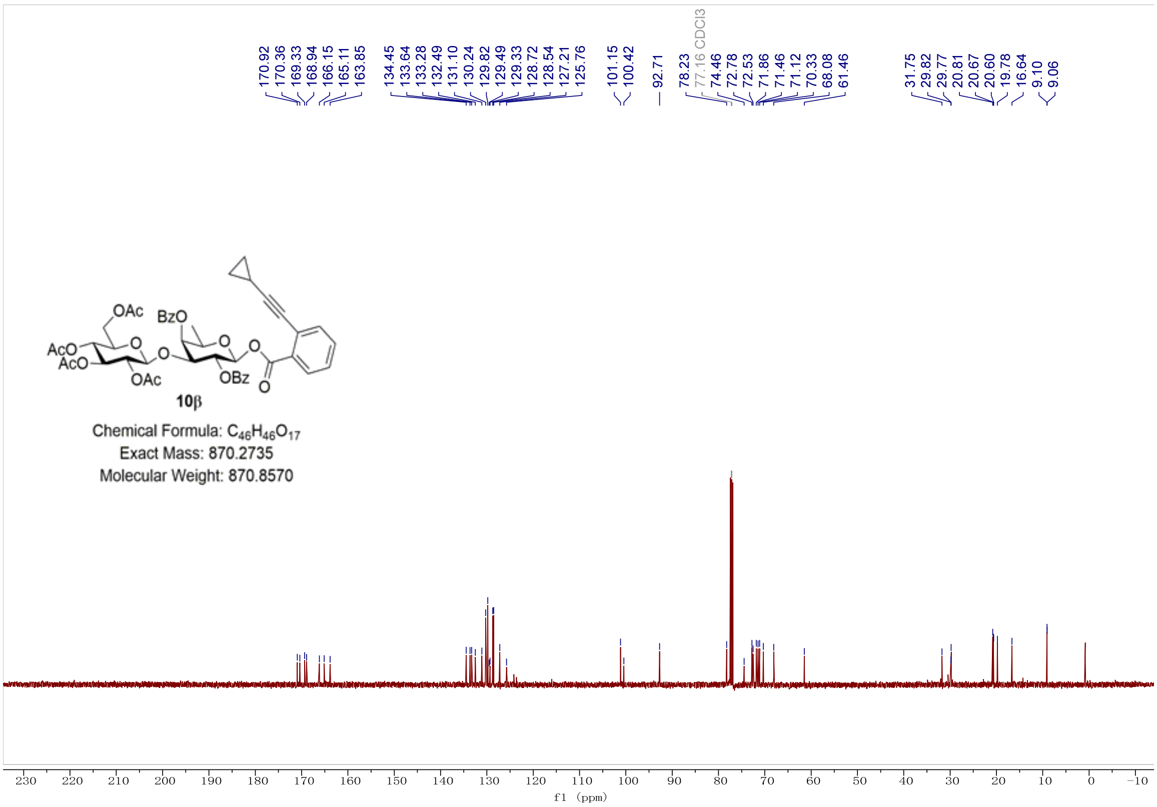

^{1}H NMR of Compound **14** (500 MHz, $CDCl_3$)

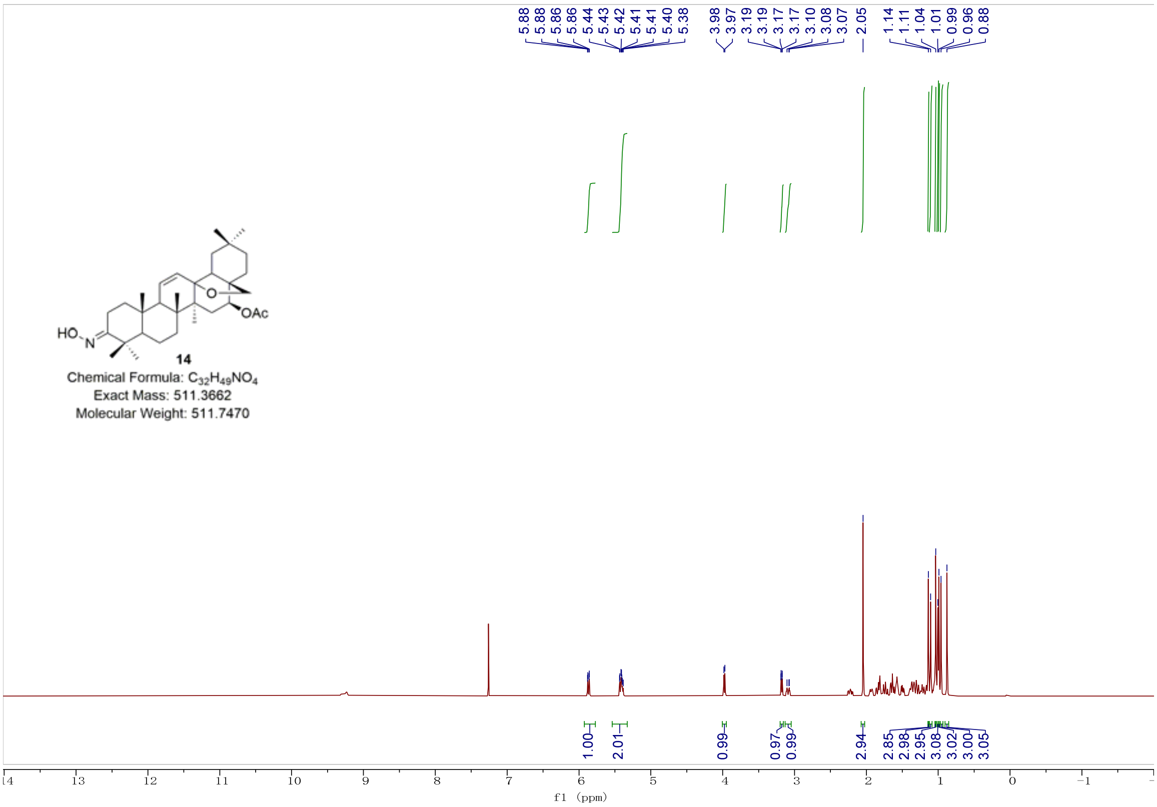

^{13}C NMR of Compound **14** (125 MHz, $CDCl_3$)

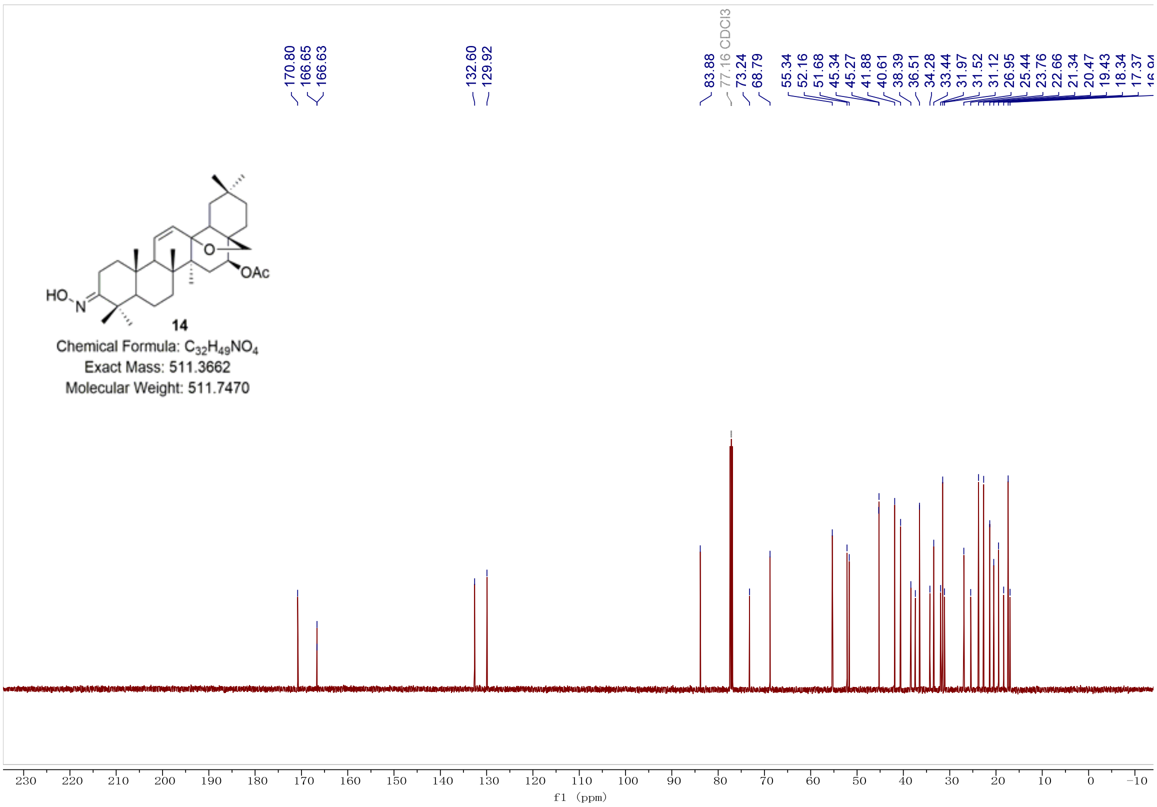

^{1}H NMR of Compound **15** (500 MHz, $CDCl_3$)

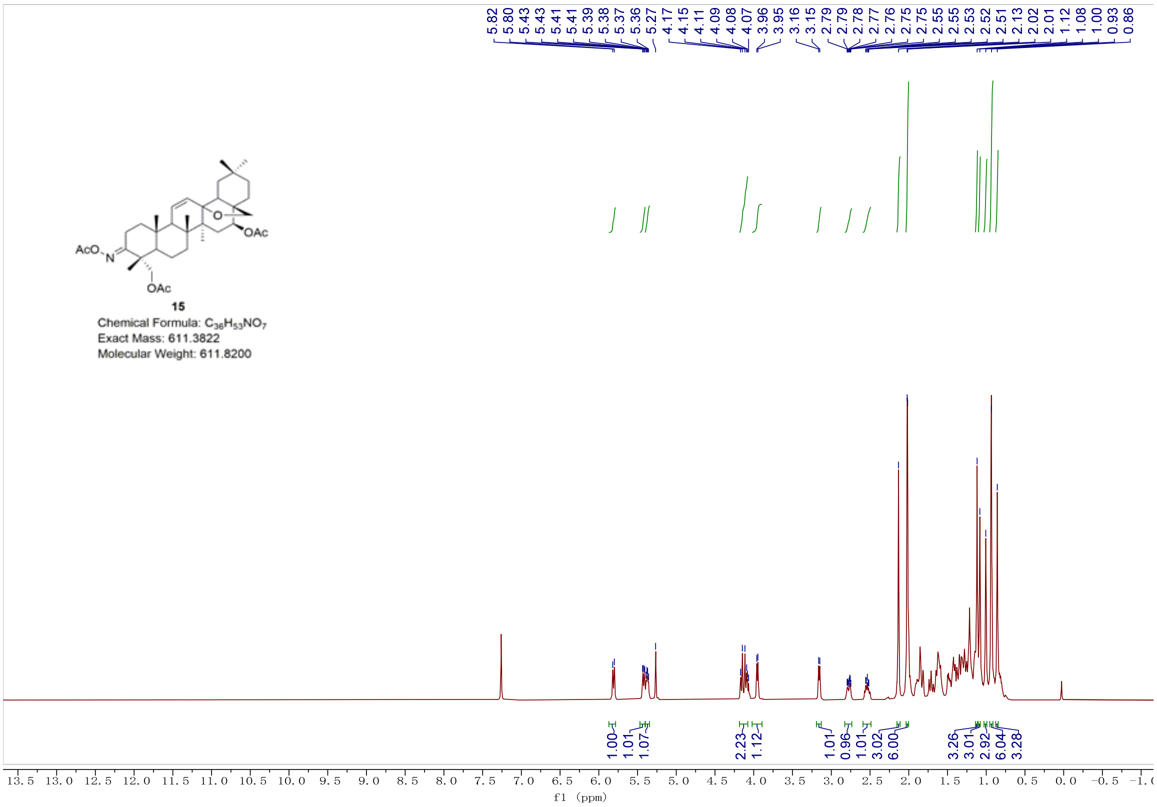

^{13}C NMR of Compound **15**(125 MHz, $CDCl_3$)

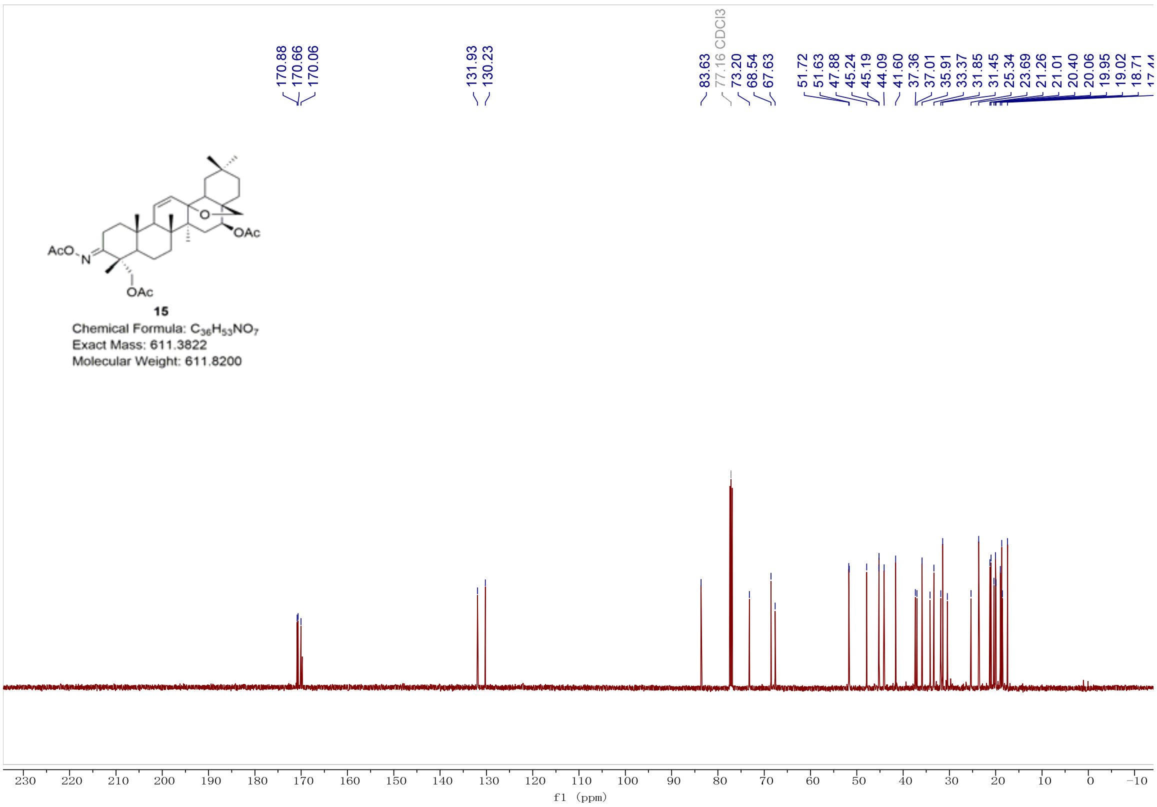

^{1}H NMR of Compound **16** (500 MHz, $CDCl_3$)

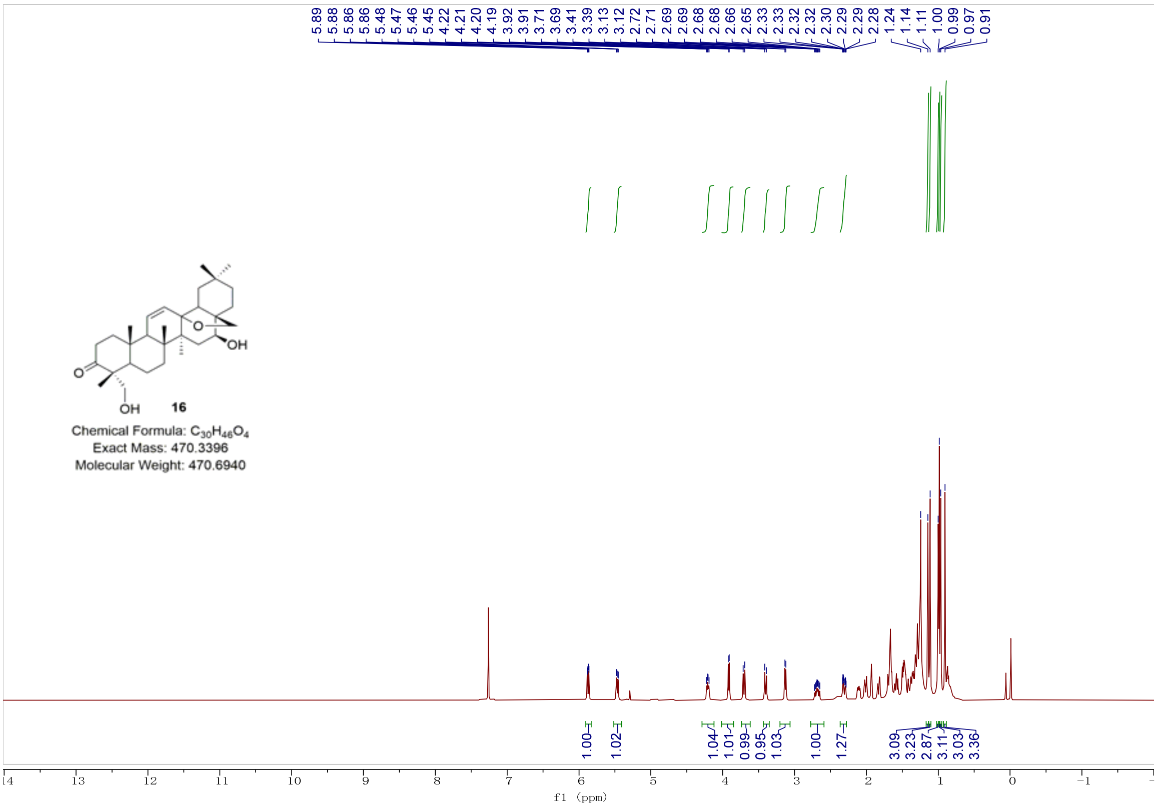

^{13}C NMR of Compound **16** (125 MHz, $CDCl_3$)

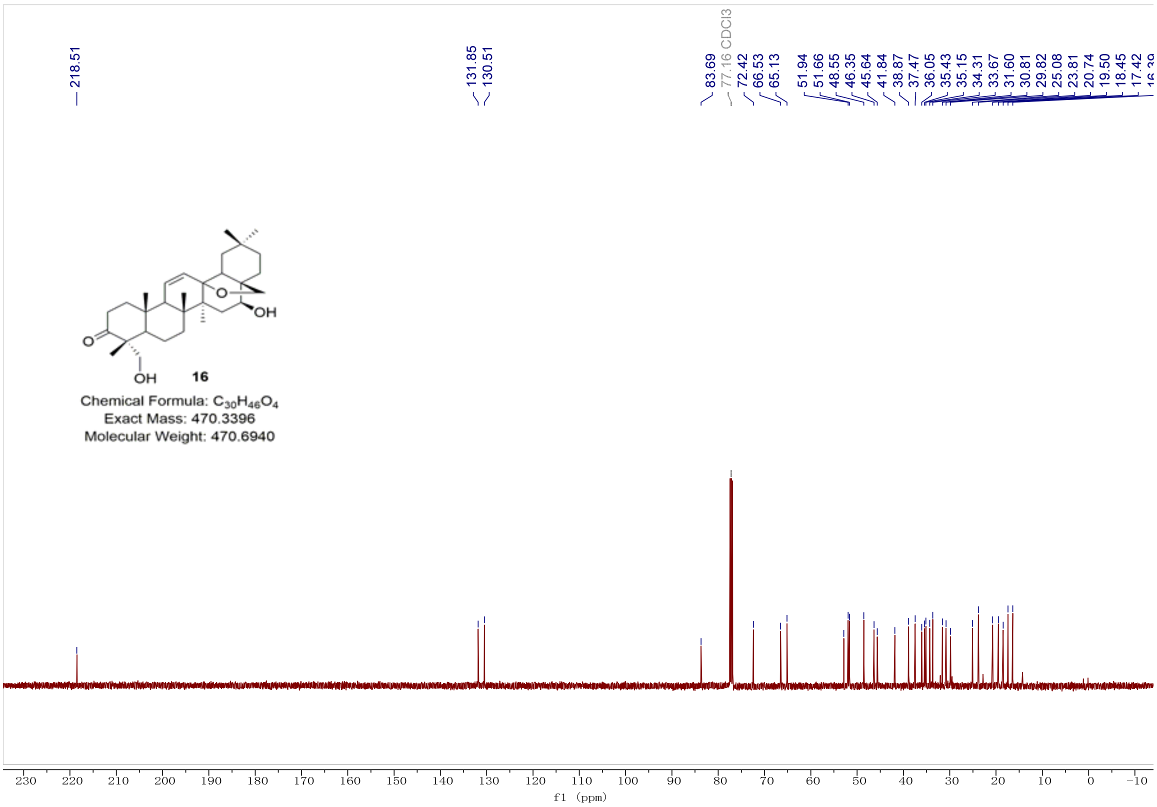

^{13}C NMR of Compound **17** (500 MHz, $CDCl_3$)

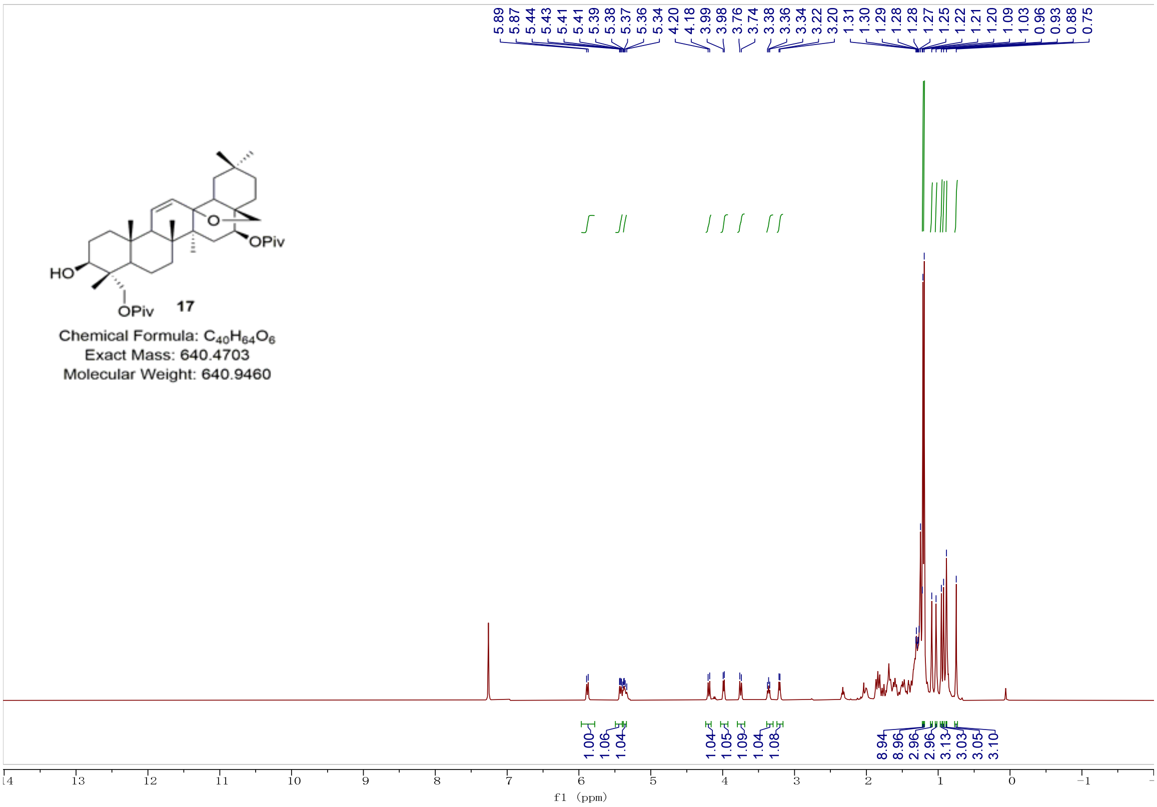

^{13}C NMR of Compound **17** (125 MHz, $CDCl_3$)

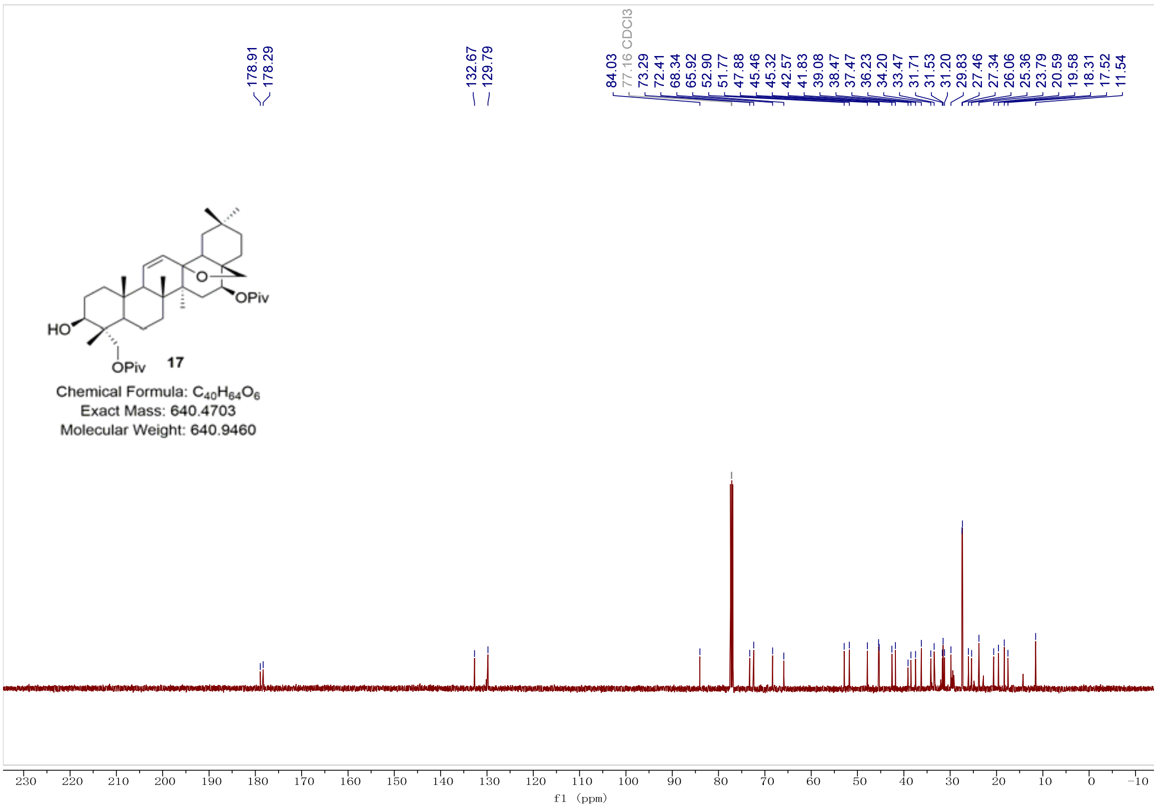

^{1}H NMR of Compound **18** (500 MHz, $CDCl_3$)

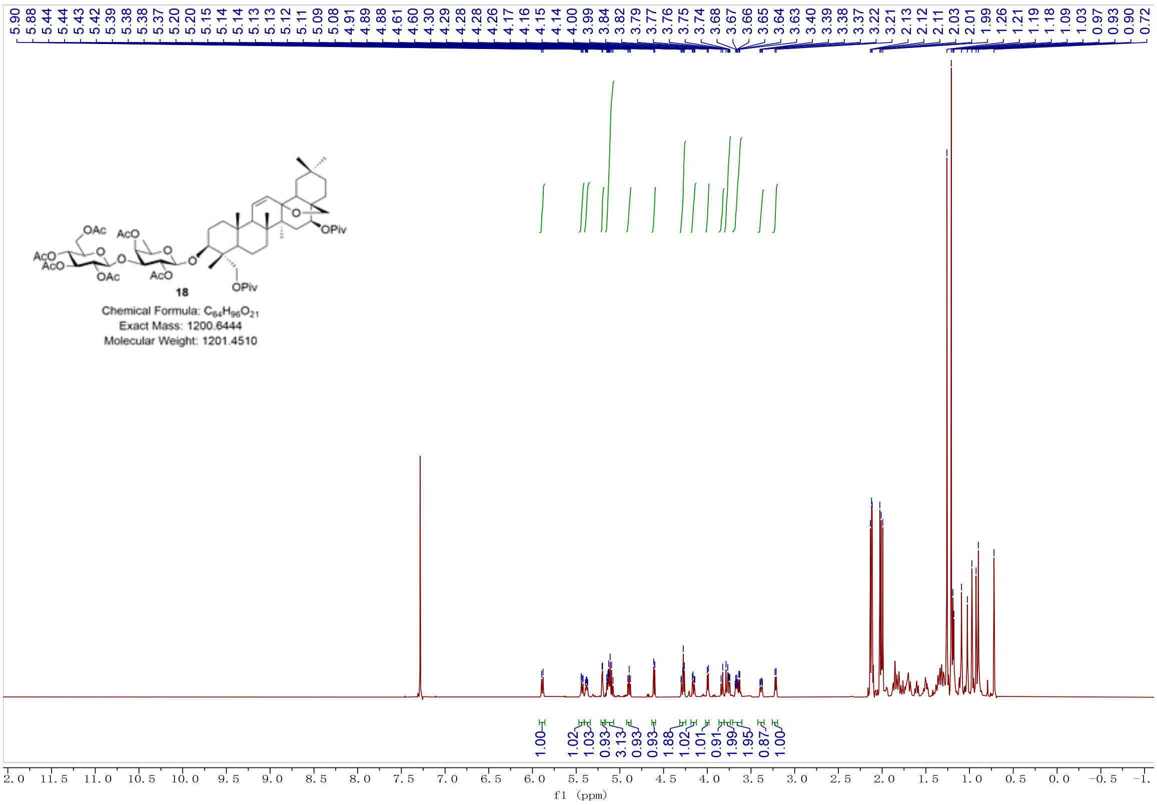

^{13}C NMR of Compound **18** (125 MHz, $CDCl_3$)

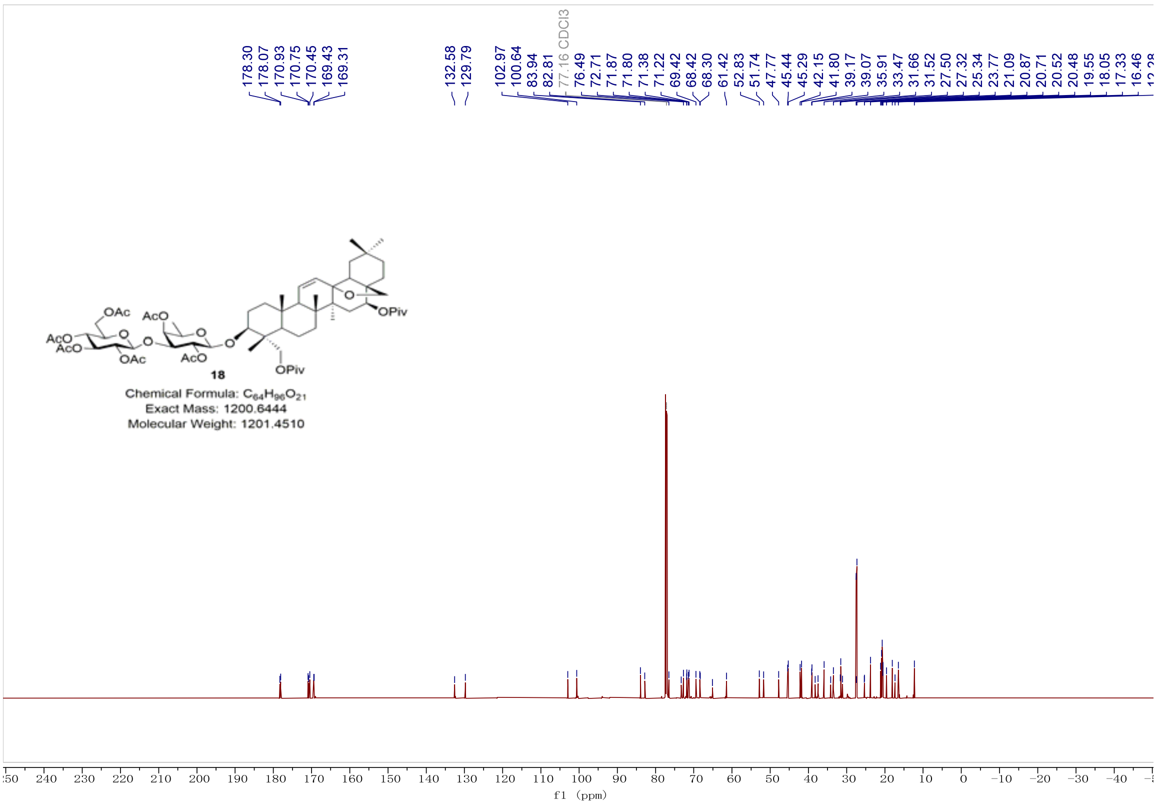

^{1}H NMR of Compound **19** (500 MHz, pyridine-d_5)

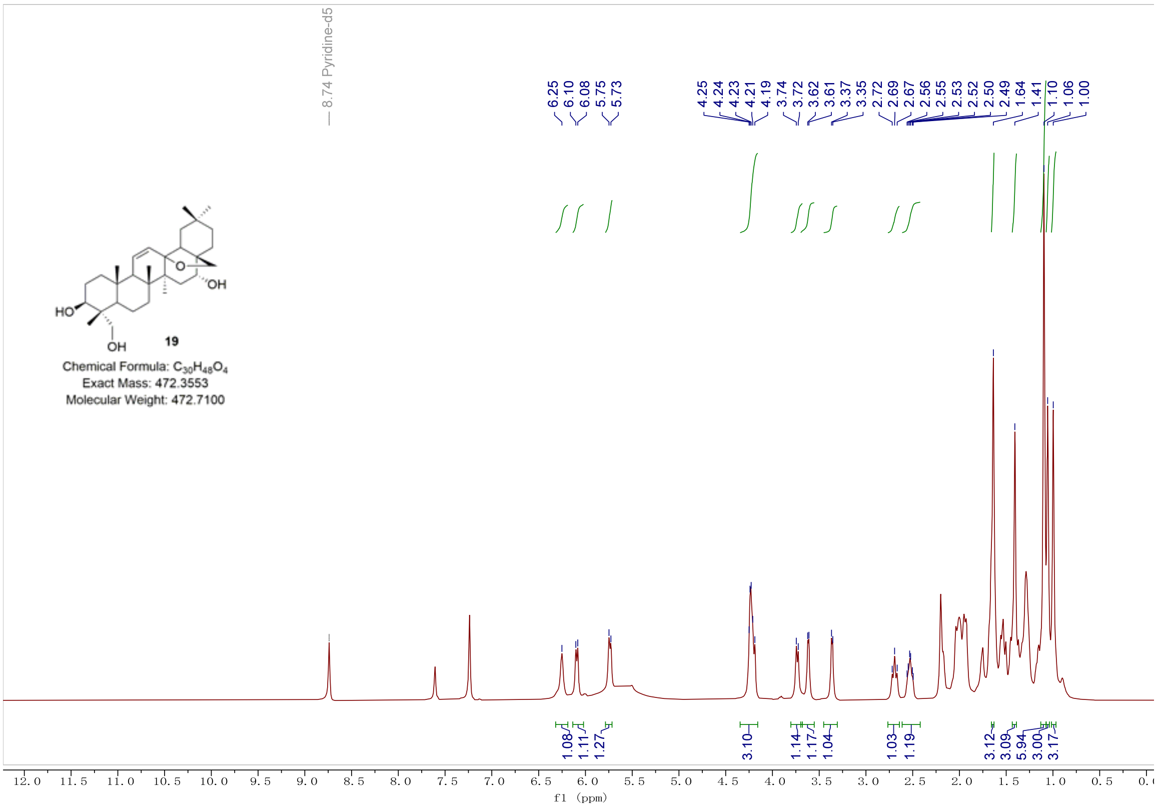

^{13}C NMR of Compound **19** (125 MHz, pyridine-d_5)

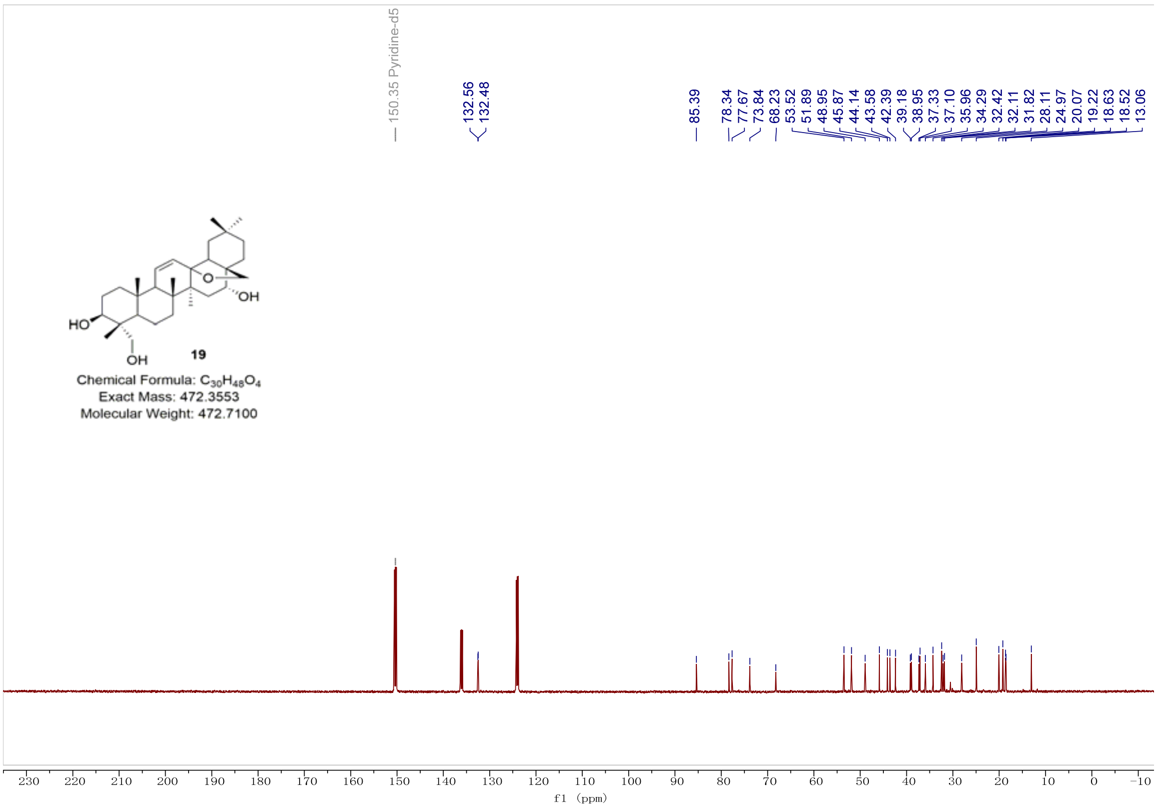

^{1}H NMR of Compound **20** (500 MHz, $CDCl_3$)

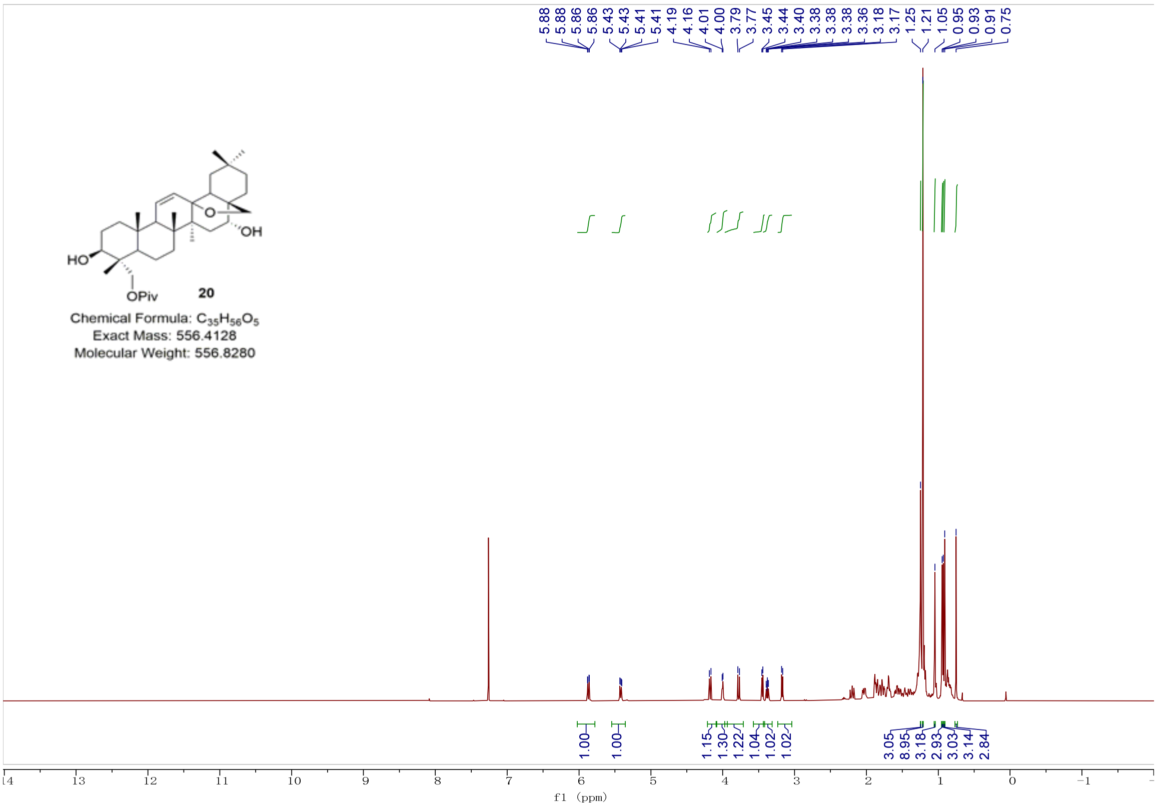

^{13}C NMR of Compound **20** (125 MHz, $CDCl_3$)

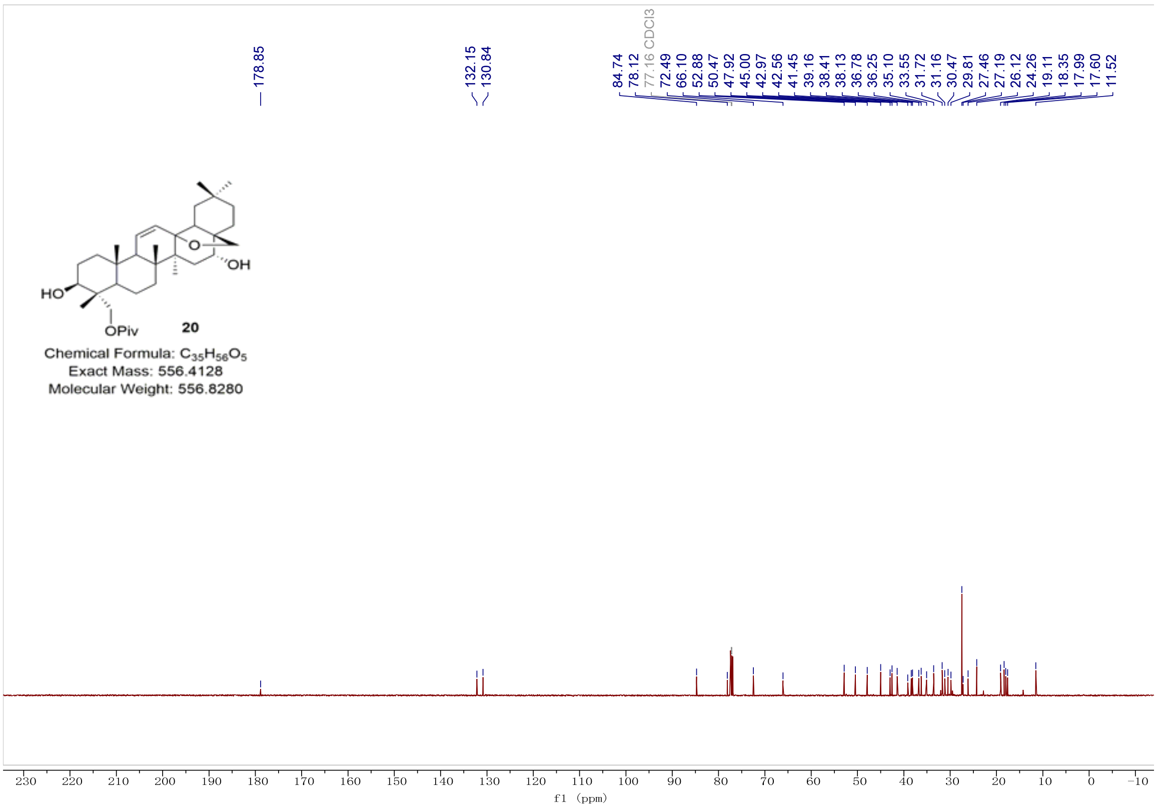

^{1}H NMR of Compound **21** (500 MHz, $CDCl_3$)

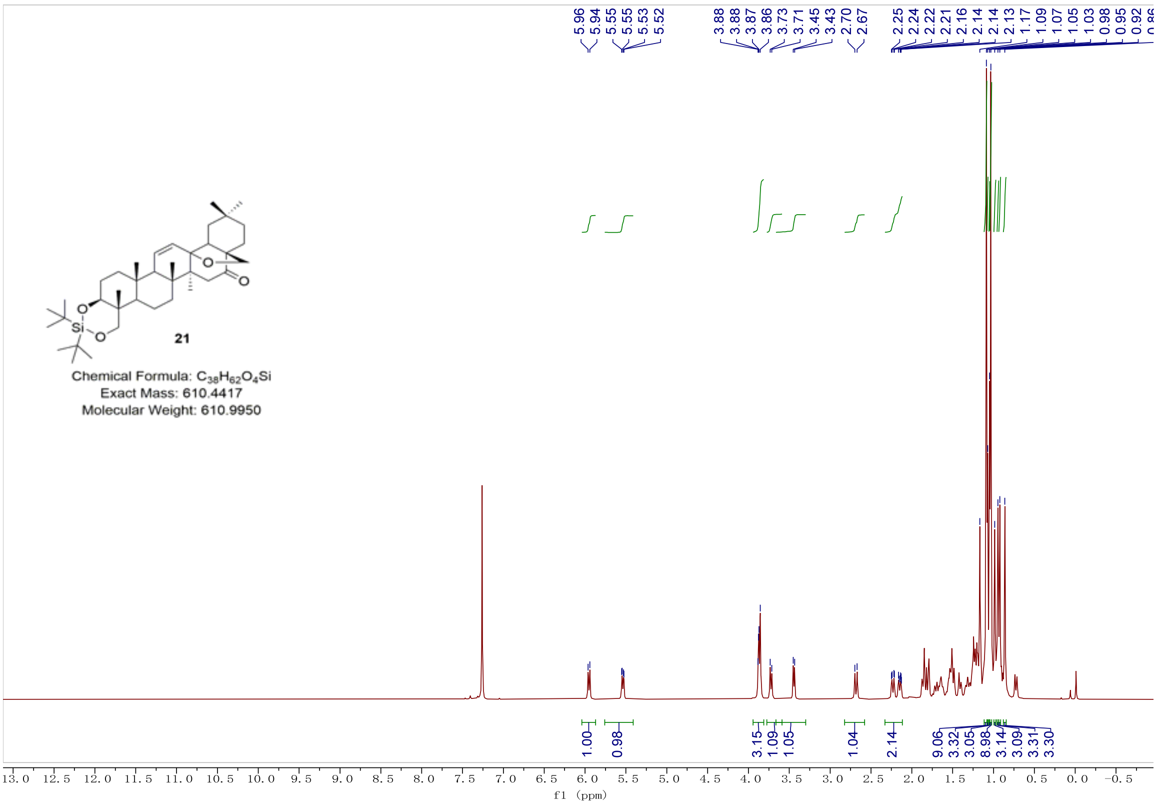

^{13}C NMR of Compound **21** (125 MHz, $CDCl_3$)

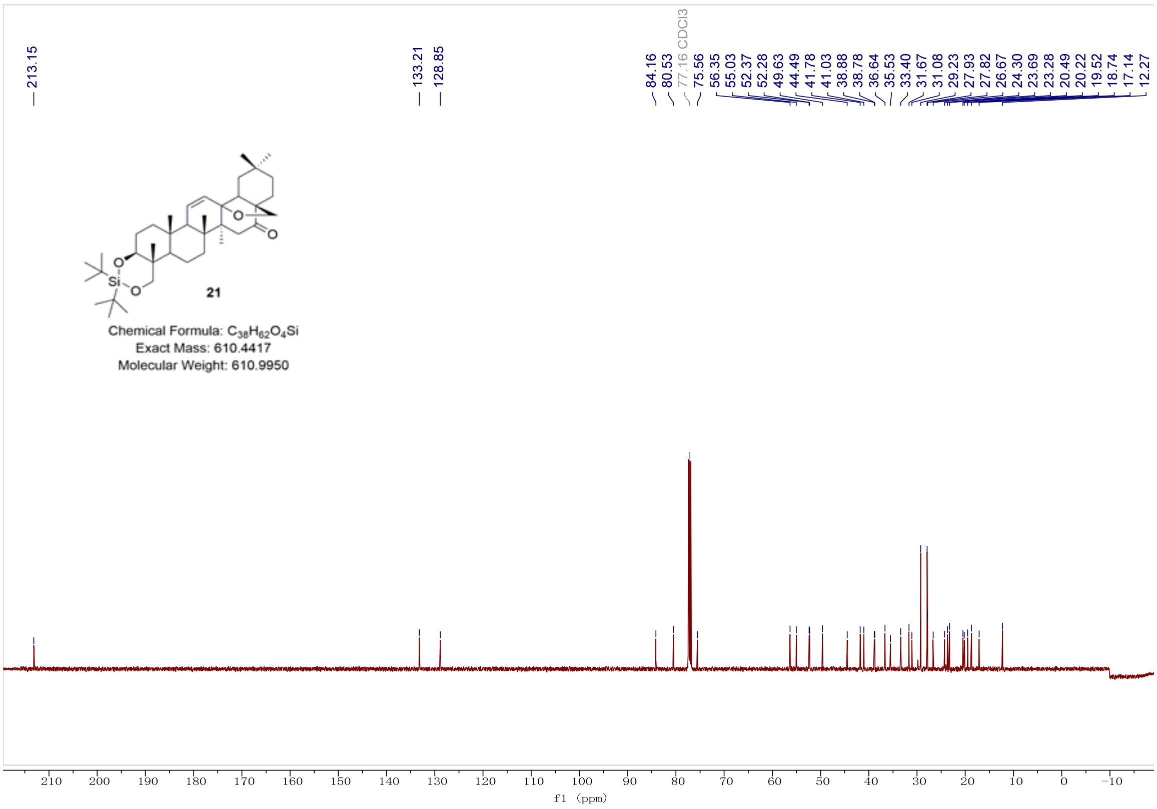

^{1}H NMR of Compound **22** (500 MHz, $CDCl_3$)

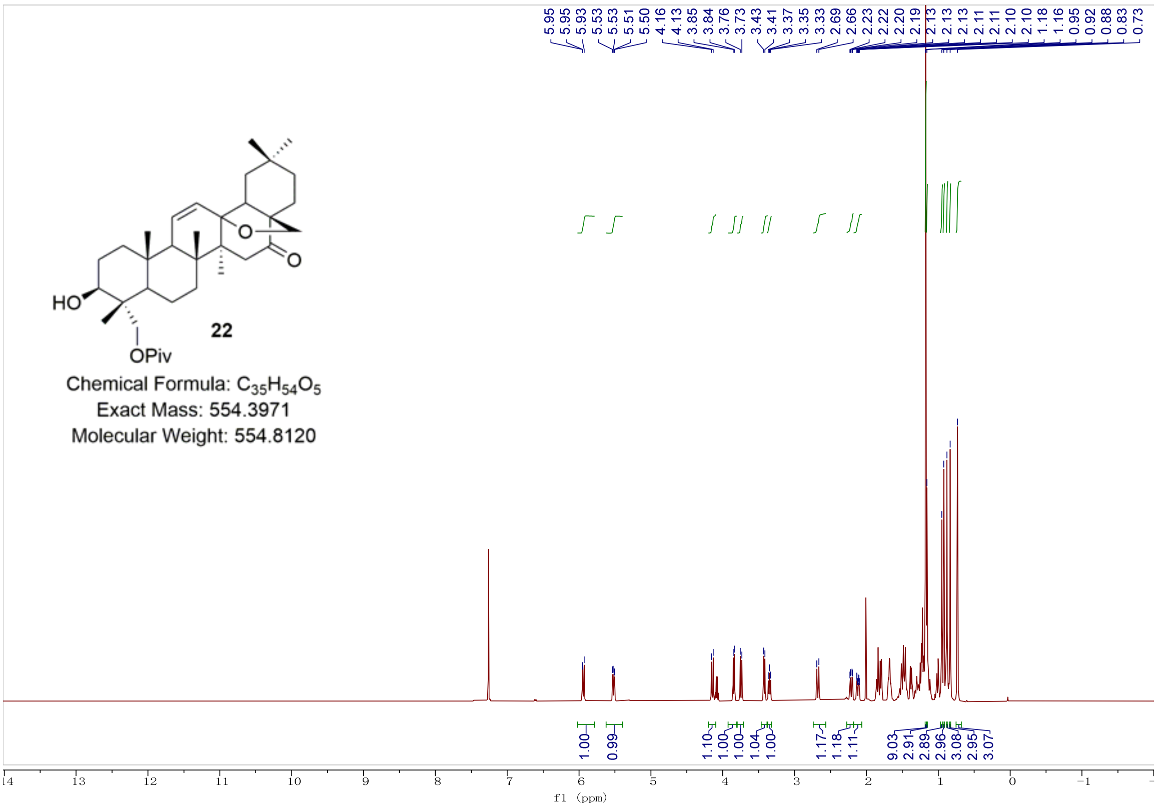

^{13}C NMR of Compound **22** (125 MHz, $CDCl_3$)

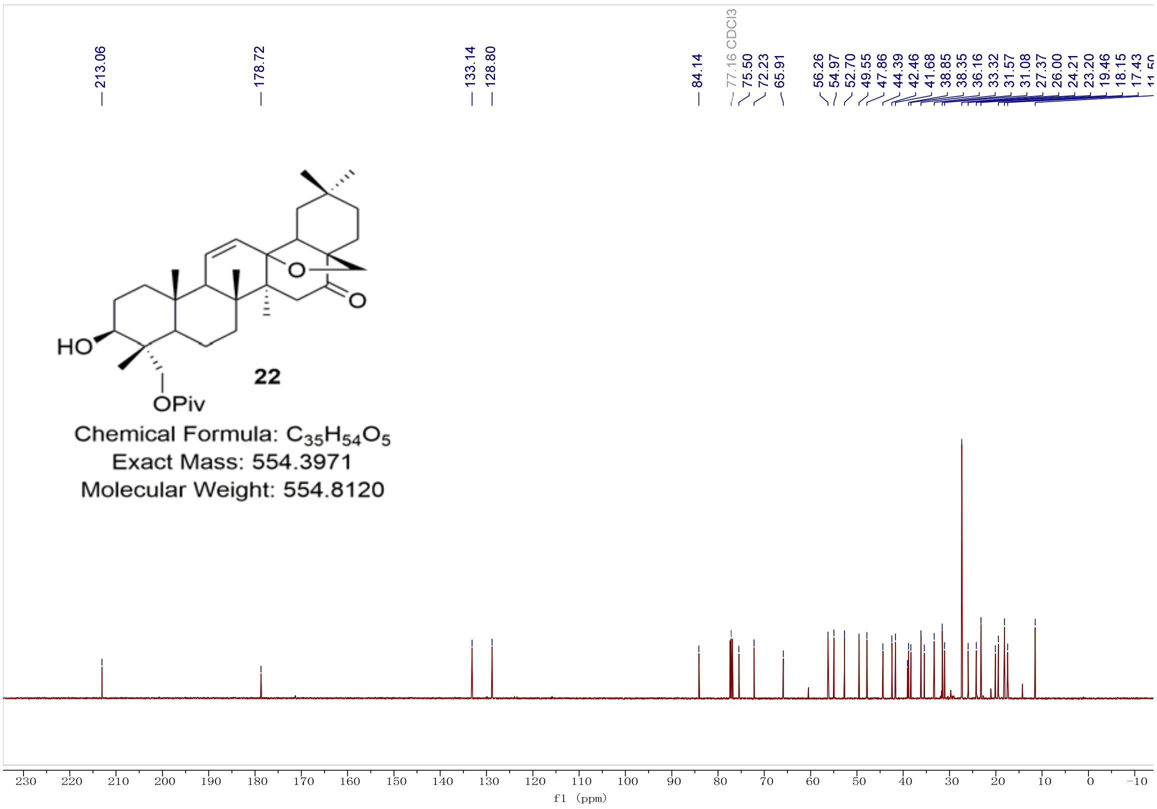

^{1}H NMR of Compound **23** (500 MHz, $CDCl_3$)

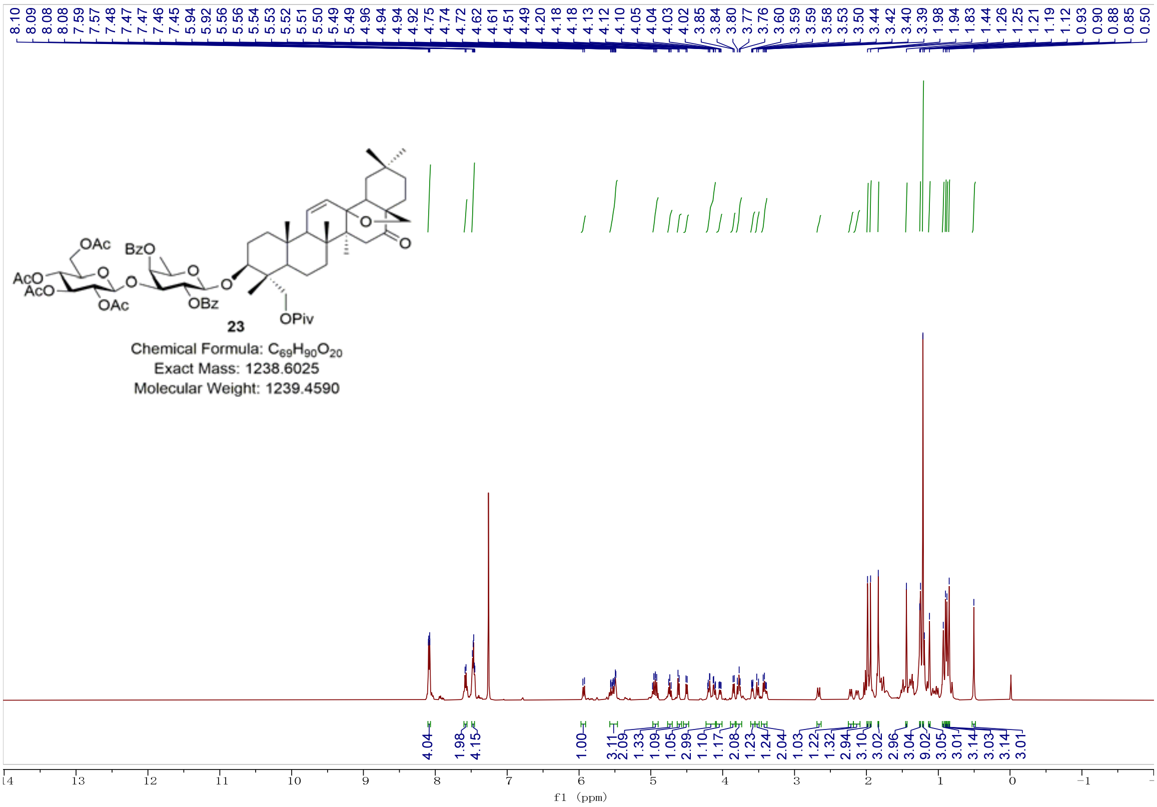

^{13}C NMR of Compound **23** (125 MHz, $CDCl_3$)

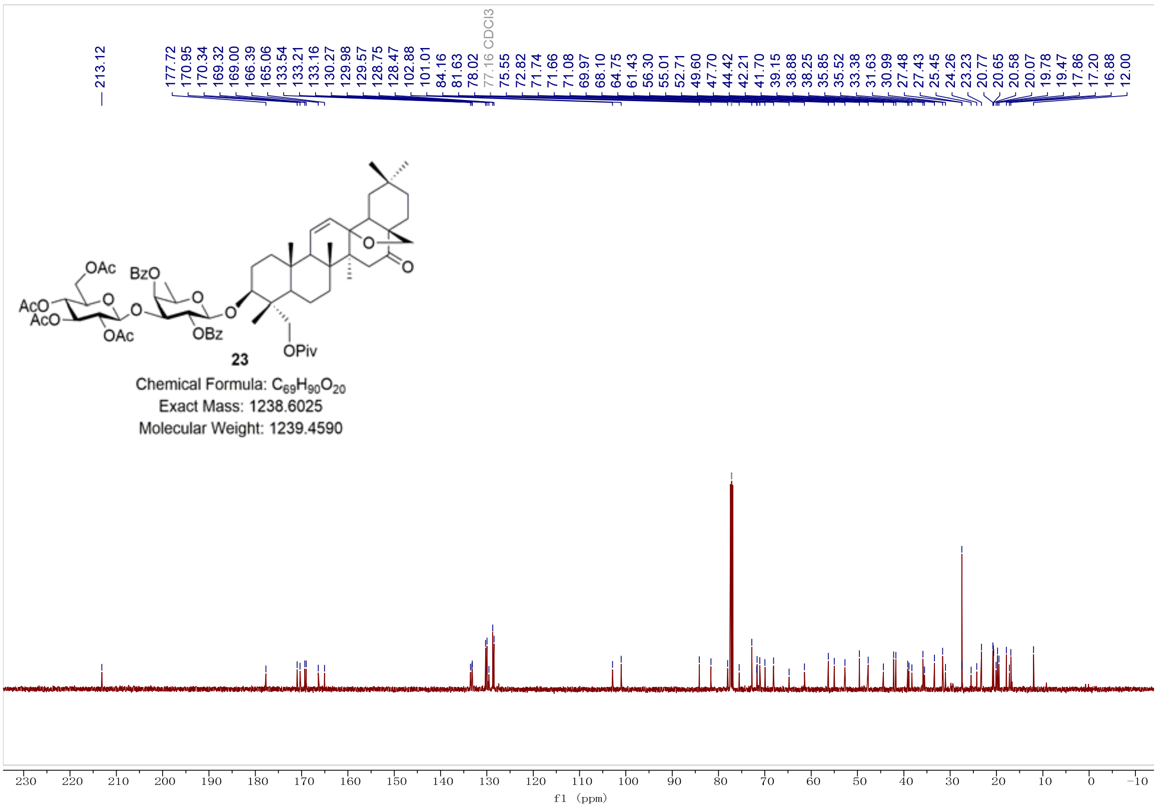

^{1}H NMR of Compound **24α** (500 MHz, $CDCl_3$)

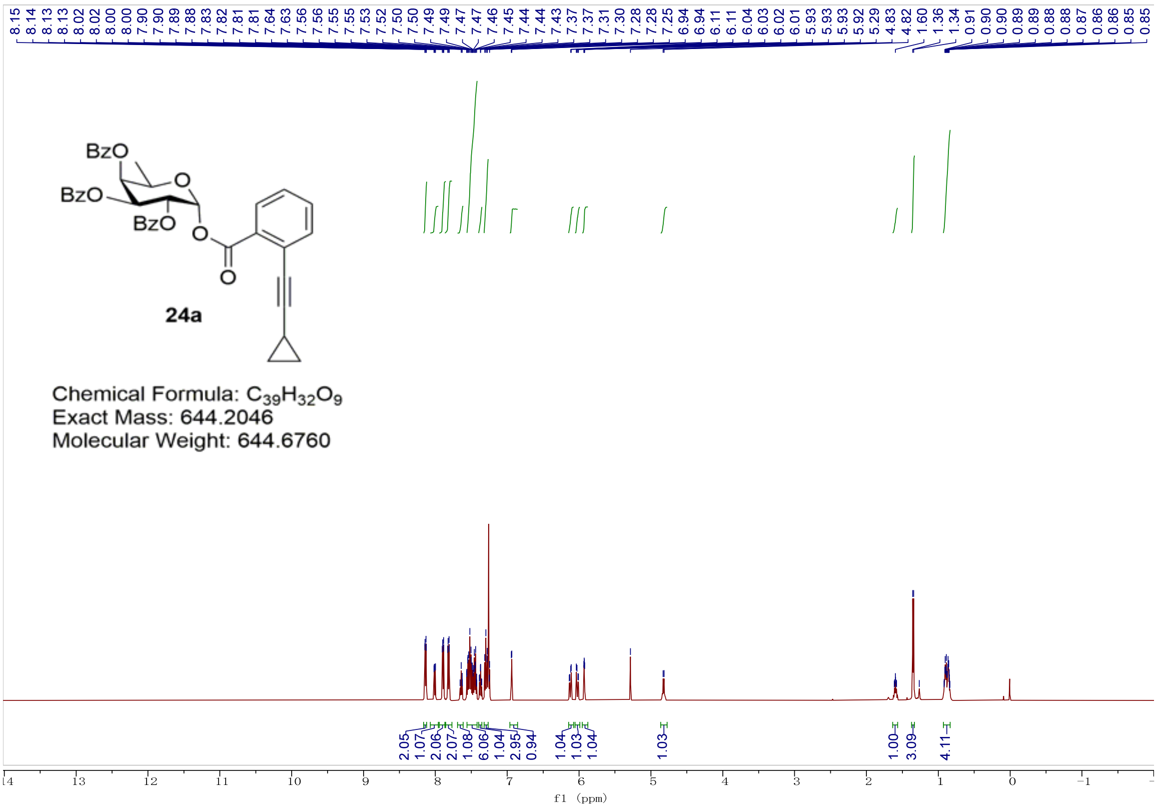

^{13}C NMR of Compound **24α** (125 MHz, $CDCl_3$)

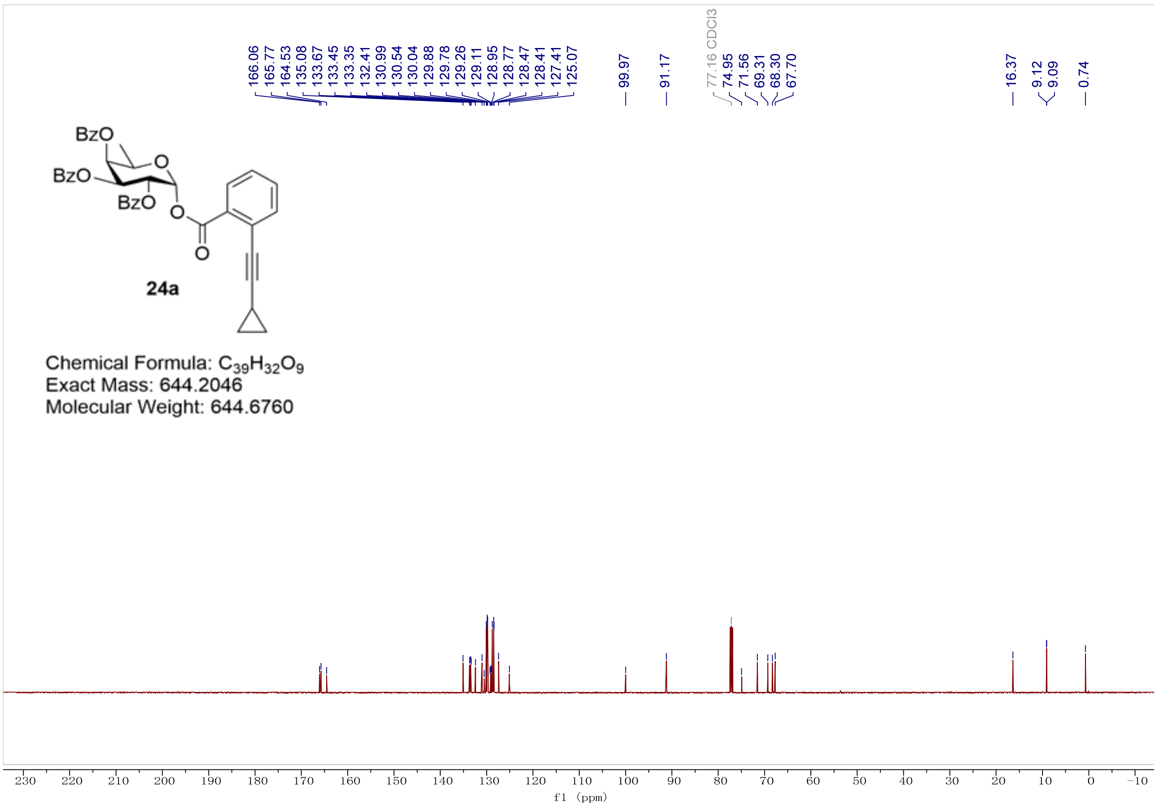

^{1}H NMR of Compound **24β** (500 MHz, $CDCl_3$)

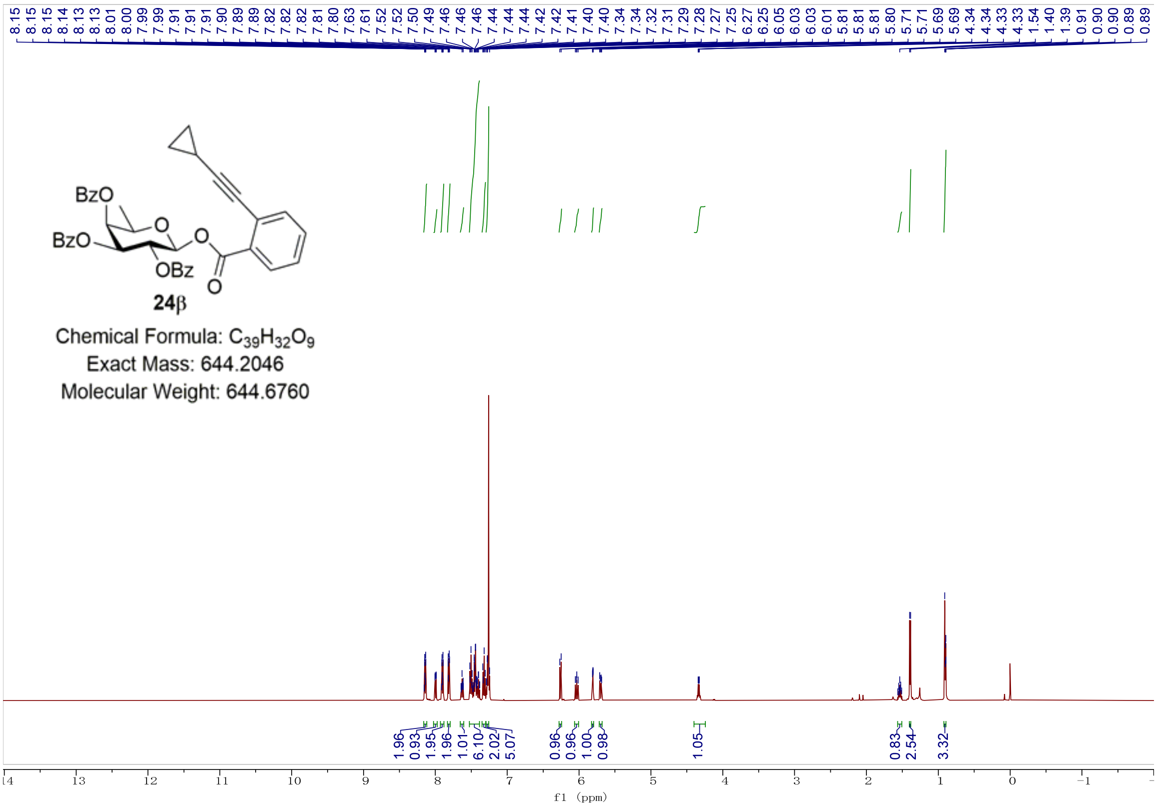

^{13}C NMR of Compound **24β** (125 MHz, $CDCl_3$)

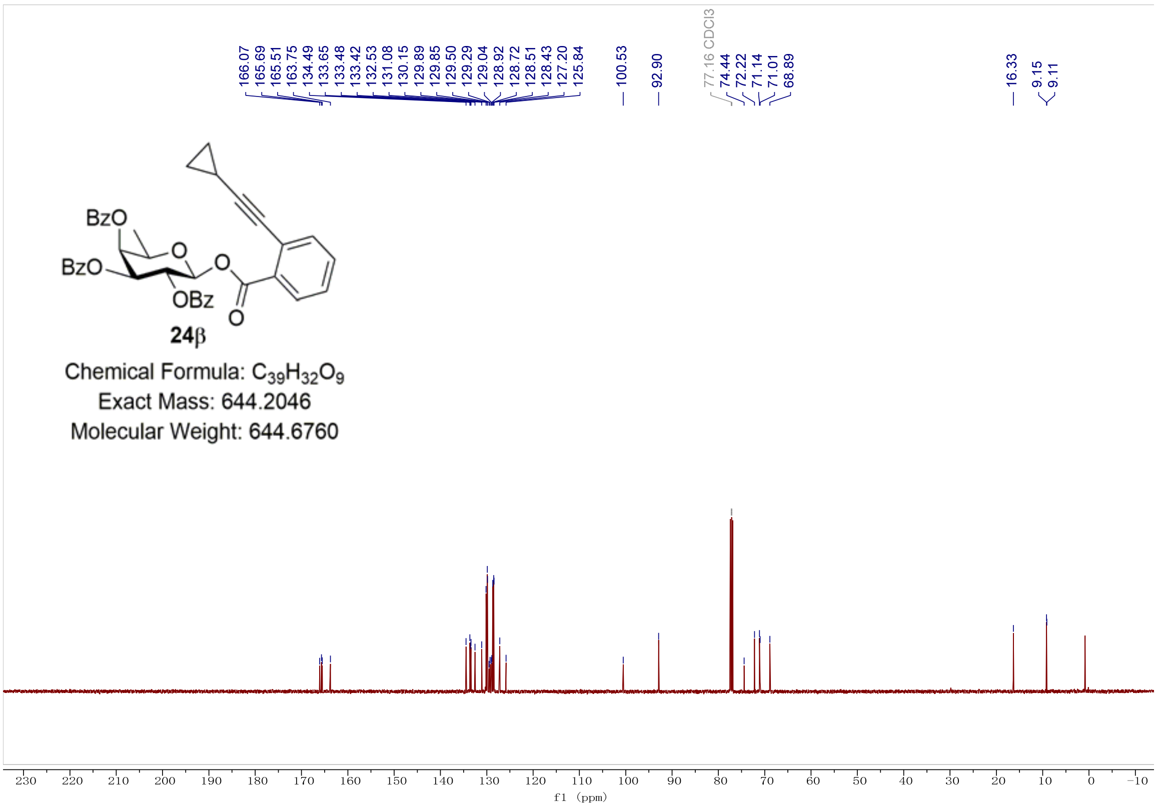

^{1}H NMR of Compound **25** (500 MHz, $CDCl_3$)

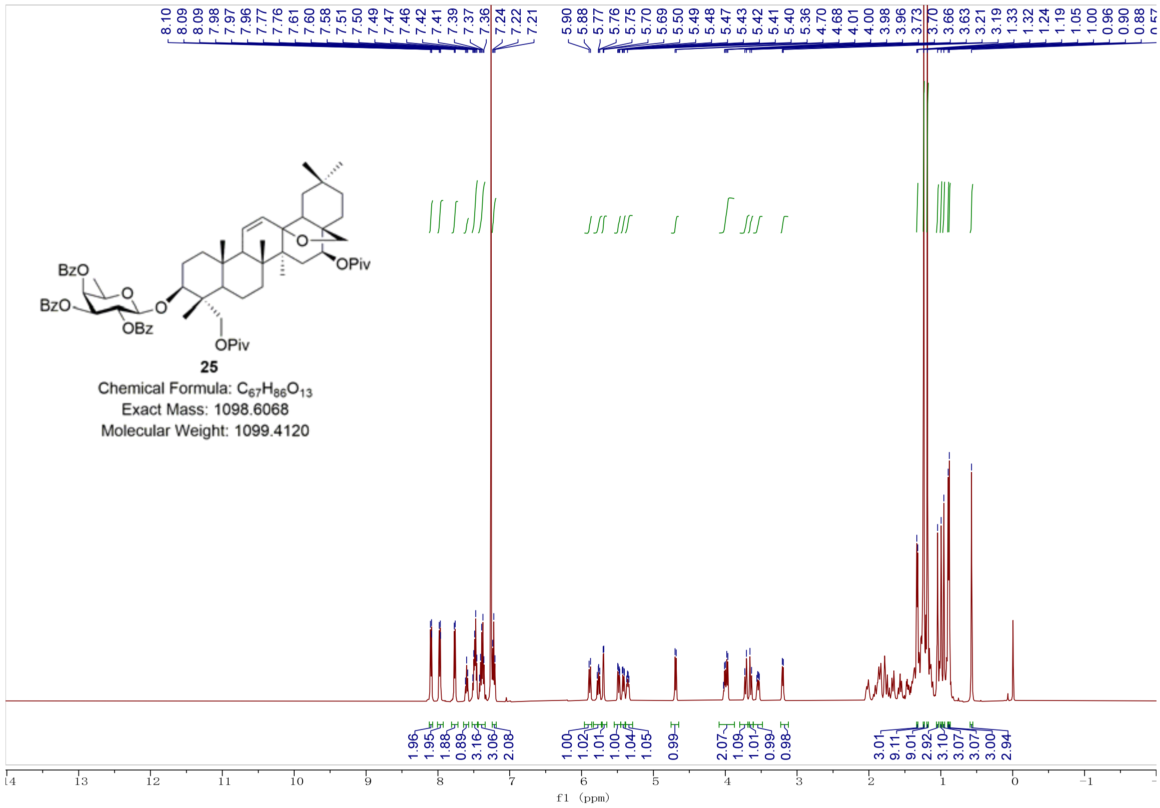

^{13}C NMR of Compound **25** (125 MHz, $CDCl_3$)

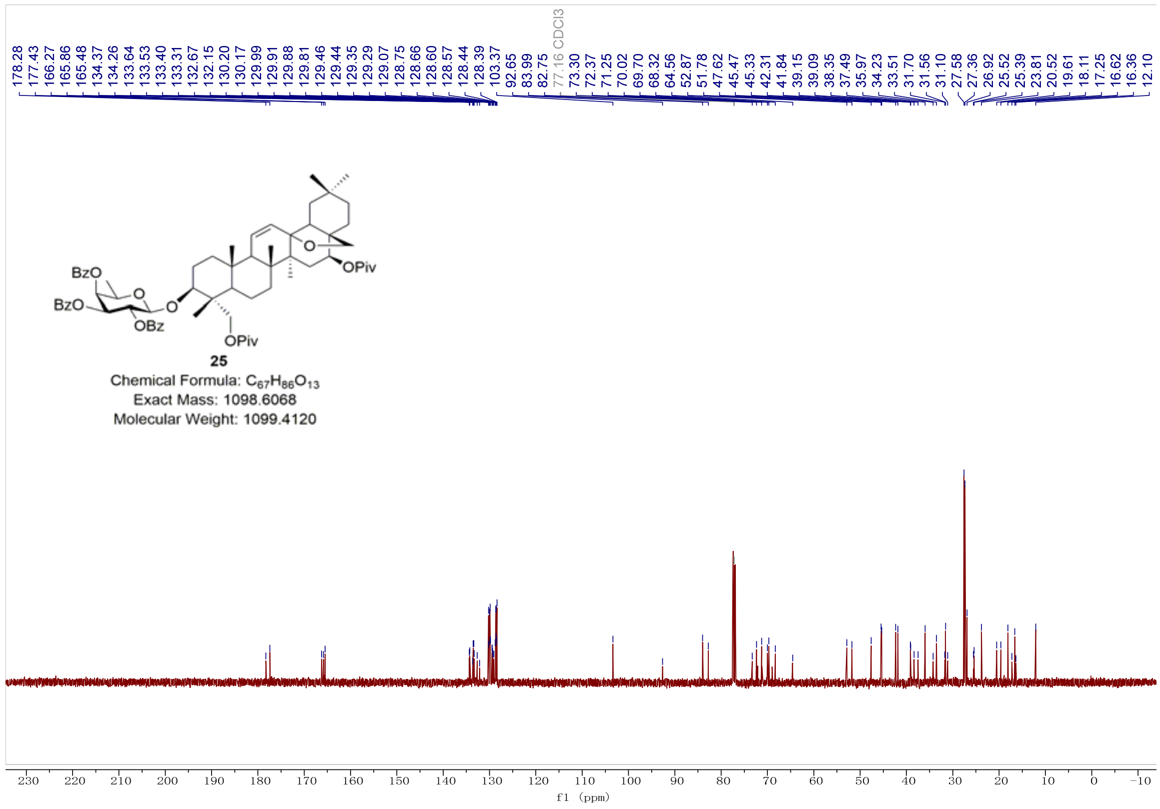

^{1}H NMR of Compound **26** (500 MHz, $CDCl_3$)

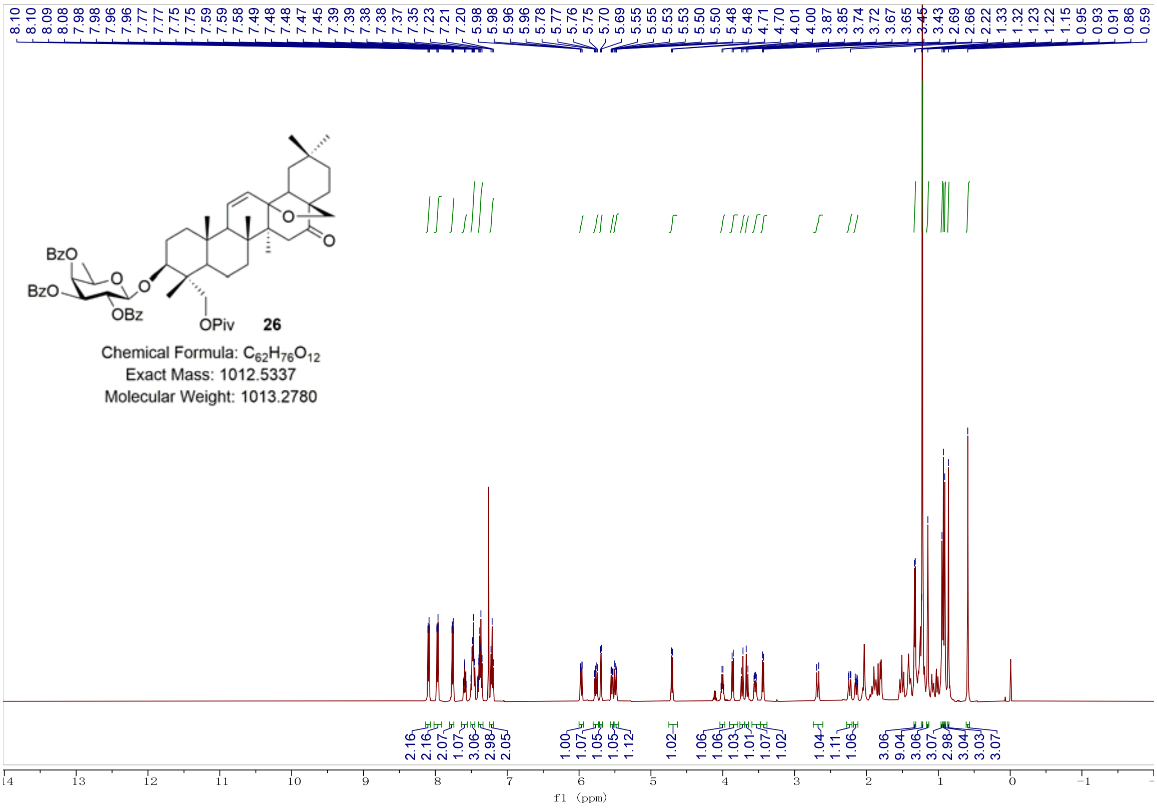

^{13}C NMR of Compound **26** (125 MHz, $CDCl_3$)

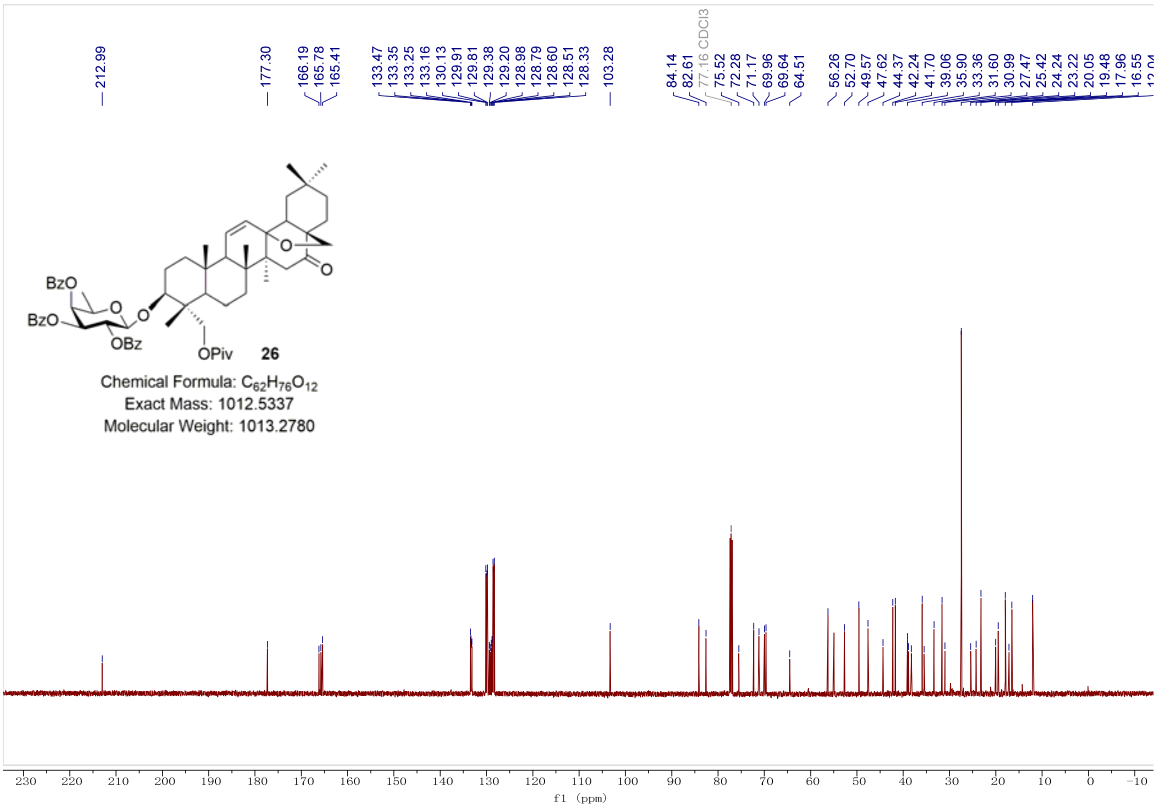

^{1}H NMR of Compound **27** (500 MHz, $CDCl_3$)

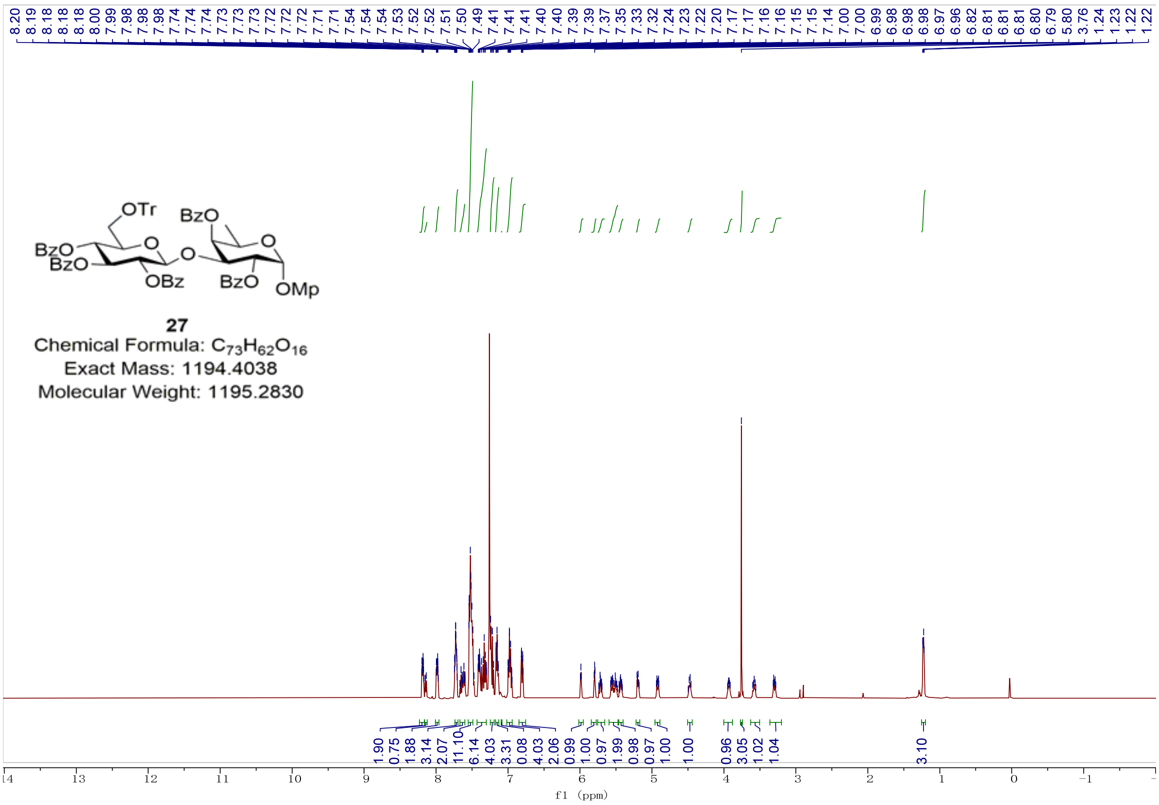

^{13}C NMR of Compound **27** (125 MHz, $CDCl_3$)

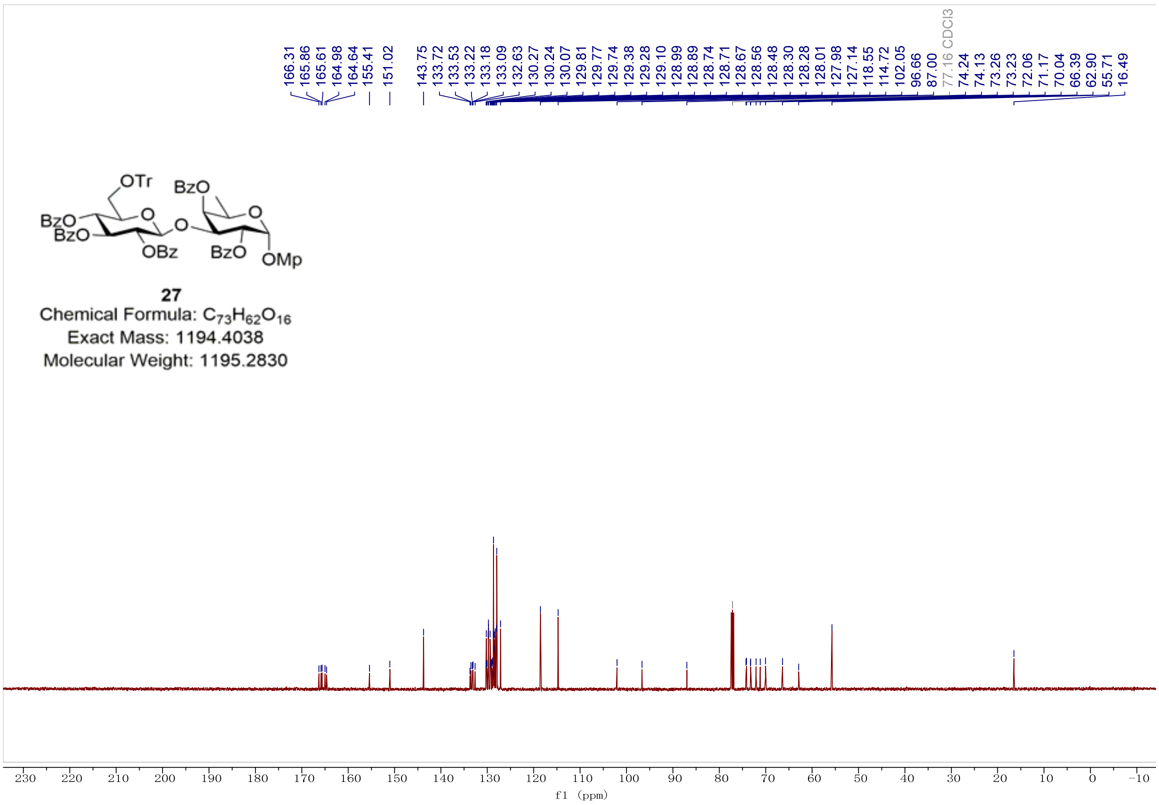

^{1}H NMR of Compound **29** (500 MHz, $CDCl_3$)

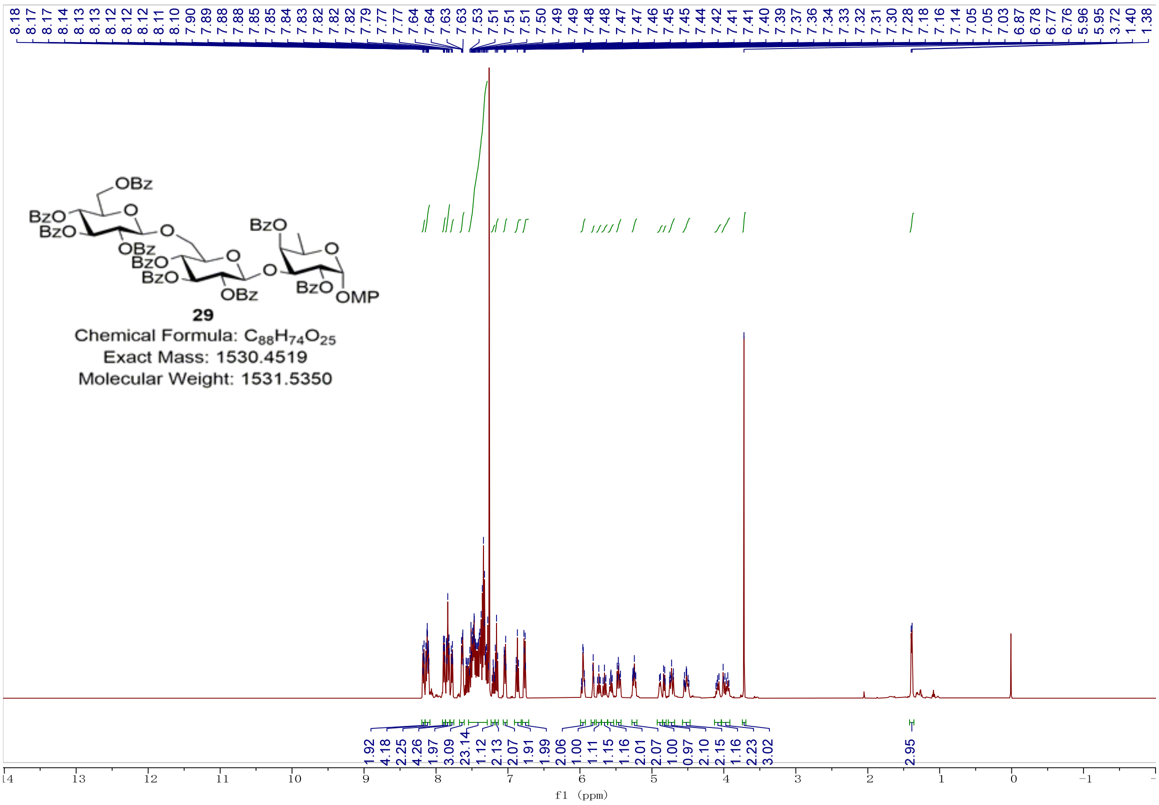

^{13}C NMR of Compound **29** (125 MHz, $CDCl_3$)

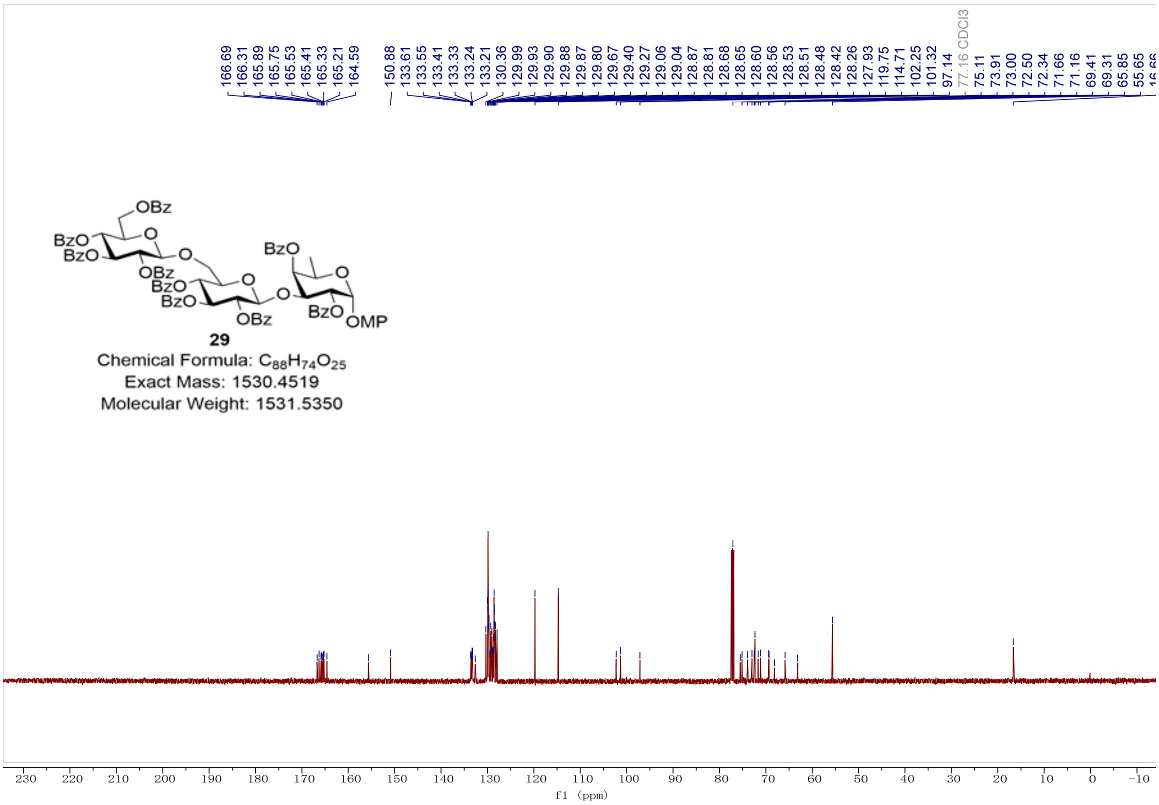

^{1}H NMR of Compound **31** (500 MHz, $CDCl_3$)

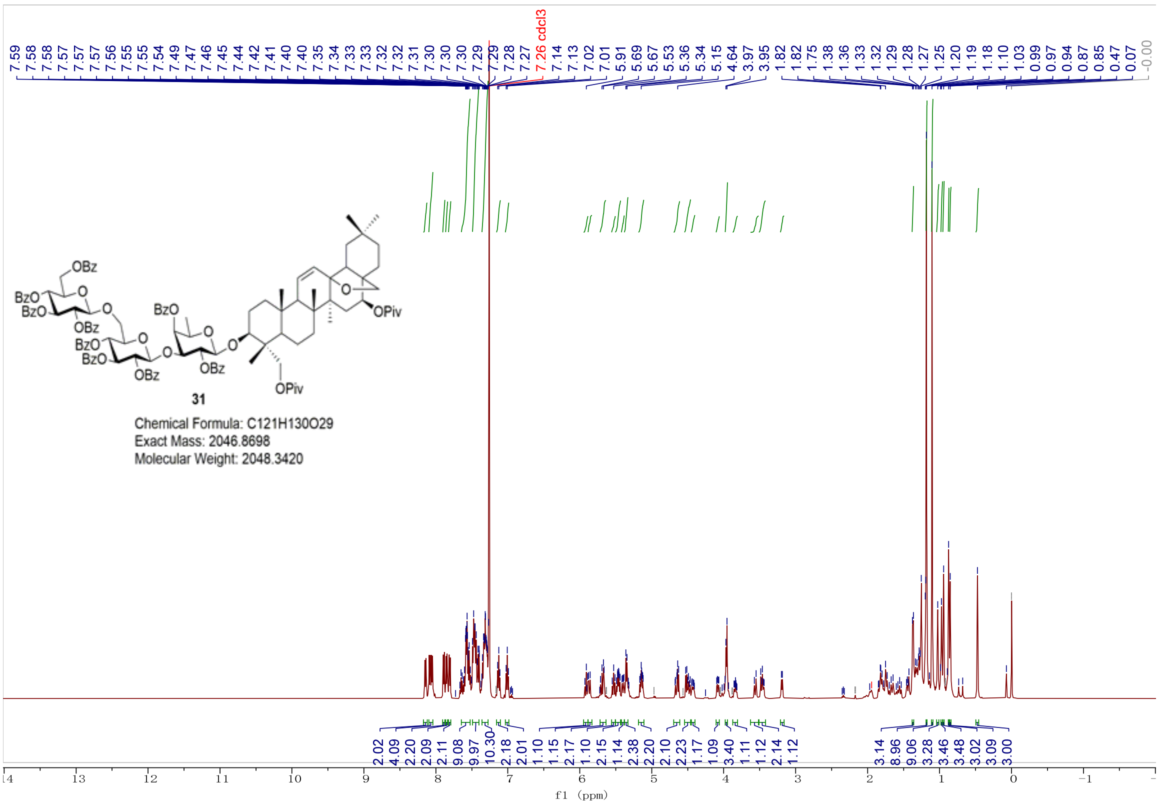

^{13}C NMR of Compound **31** (125 MHz, $CDCl_3$)

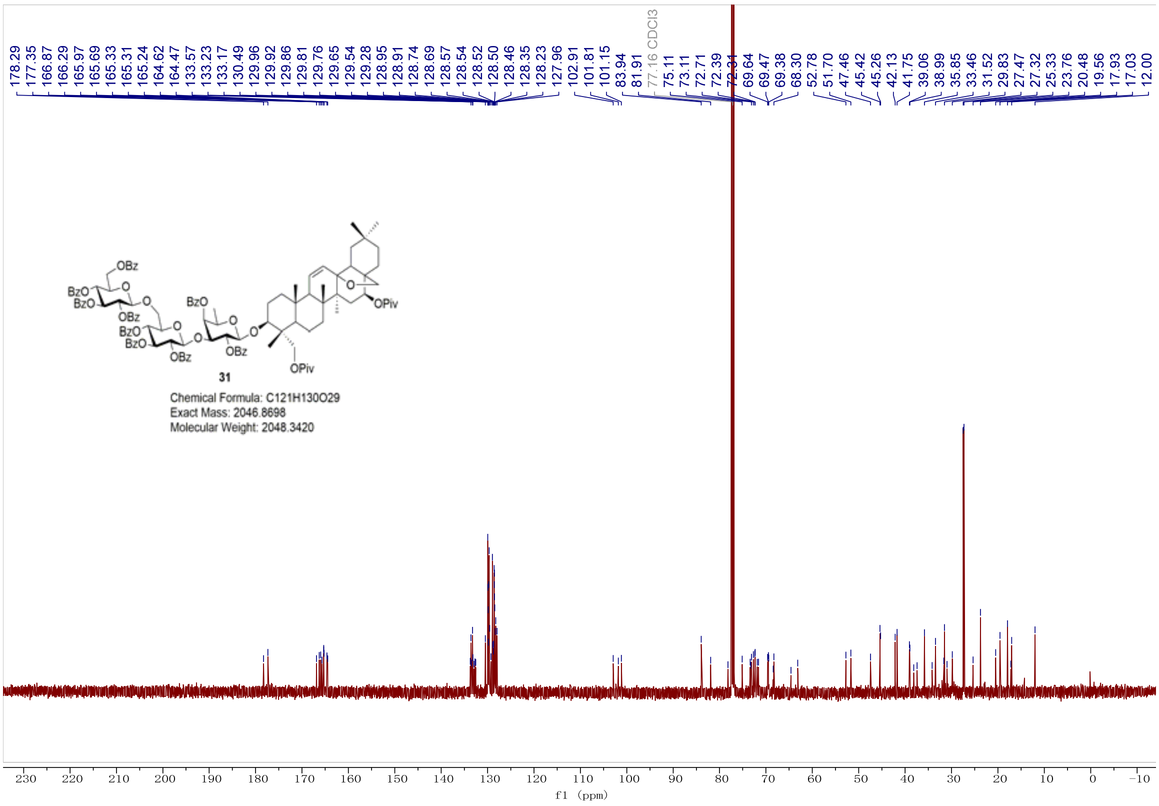

Comparison of ^{13}C NMR data of the synthetic 1-5 and 7 with those reported for the natural products

Table 1. Comparison of the ^{13}C NMR data of synthetic **1** with those of the natural Saikosaponin A

Position	^{13}C		
	Reported[S1]	Synthetic **1**	Δδ
1	38.60	38.60	0
2	26.00	26.06	-0.06
3	81.60	81.53	0.07
4	43.70	43.68	0.02
5	47.30	47.23	0.07
6	17.20	17.49	-0.29
7	31.50	31.55	-0.05
8	42.20	42.15	0.05
9	53.00	53.03	-0.03
10	36.20	36.21	-0.01
11	132.20	132.14	0.06
12	131.10	131.11	-0.01
13	84.00	83.93	0.07
14	45.60	45.58	0.02
15	36.10	36.06	0.04
16	64.00	64.00	0
17	46.90	46.94	-0.04
18	52.10	52.09	0.01
19	37.74	37.67	0.07
20	31.50	31.52	-0.02
21	34.60	34.63	-0.03
22	25.70	25.70	0
23	64.00	63.92	0.08
24	13.00	13.00	0
25	18.70	18.69	0.01
26	20.00	19.99	0.01
27	20.80	20.79	0.01
28	73.00	72.97	0.03
29	33.60	33.59	0.01
30	23.80	23.77	0.03
C-1'	105.90	105.95	-0.05
C-2'	71.50	71.49	0.01
C-3'	85.10	85.12	-0.02
C-4'	71.70	71.77	-0.07
C-5'	70.90	70.96	-0.06
C-6'	17.20	17.22	-0.02
C-1''	106.50	106.58	-0.08
C-2''	75.70	75.76	-0.06

C-3"	78.70	78.71	-0.01
C-4"	72.10	72.09	0.01
C-5"	78.40	78.36	0.04
C-6"	62.70	62.64	0.06

Table 2. Comparison of the ^{13}C NMR data of synthetic **2** with those of the natural natural Saikosaponin D

Position	^{13}C		
	Reported[S2]	Synthetic **2**	Δδ
1	39.00	39.05	-0.05
2	26.50	26.53	-0.03
3	82.00	82.04	-0.04
4	44.00	43.99	0.01
5	47.80	47.75	0.05
6	18.00	17.95	0.05
7	32.30	32.29	0.01
8	42.30	42.27	0.03
9	53.40	53.42	-0.02
10	37.20	37.20	0
11	132.40	132.3	0.1
12	132.40	132.36	0.04
13	85.30	85.28	0.02
14	44.10	44.14	-0.04
15	36.70	36.68	0.02
16	77.50	77.52	-0.02
17	45.70	45.73	-0.03
18	51.70	51.74	-0.04
19	38.80	38.76	0.04
20	31.90	31.93	-0.03
21	35.90	35.85	0.05
22	31.70	31.69	0.01
23	64.40	64.43	-0.03
24	13.50	13.47	0.03
25	18.50	18.49	0.01
26	19.20	19.23	-0.03
27	19.90	19.94	-0.04
28	78.20	78.20	0
29	34.10	34.15	-0.05
30	24.80	24.79	0.01
C-1'	106.40	106.41	-0.01
C-2'	72.00	71.93	0.07
C-3'	85.60	85.64	-0.04

C-4'	72.20	72.22	-0.02
C-5'	71.40	71.42	-0.02
C-6'	17.60	17.65	-0.05
C-1"	107.20	107.10	0.1
C-2"	76.20	76.23	-0.03
C-3"	79.20	79.20	0
C-4"	72.60	72.55	0.05
C-5"	78.90	78.83	0.07
C-6"	63.10	63.08	0.02

Table 3. Comparison of ^{13}C NMR data of the synthetic **3** with those of the natural Prosaikogenin F[S1]

Position	^{13}C		
	Reported[S1]	Synthetic **3**	Δδ
1	38.60	38.62	-0.02
2	26.00	25.98	0.01
3	81.70	81.59	0.11
4	43.70	43.66	0.03
5	47.40	47.35	0.05
6	17.40	17.55	-0.15
7	31.60	31.58	0.02
8	42.20	42.18	0.02
9	53.10	53.06	0.04
10	36.30	36.28	0.02
11	132.20	132.15	0.05
12	131.20	131.14	0.06
13	84.00	83.95	0.05
14	45.70	45.61	0.09
15	36.10	36.10	0
16	64.00	64.01	-0.01
17	47.00	46.97	0.03
18	52.10	52.12	-0.02
19	37.80	37.70	0.1
20	31.60	31.81	-0.21
21	34.70	34.66	0.04
22	25.70	25.73	-0.03
23	64.30	64.22	0.08
24	13.00	13.00	0
25	18.80	18.76	0.04
26	20.00	20.04	-0.04
27	20.90	20.83	0.07
28	72.80	72.93	-0.13

29	33.60	33.62	-0.02
30	23.80	23.80	0
C-1’	106.20	106.30	-0.1
C-2’	73.00	72.99	0.01
C-3’	75.50	75.46	0.04
C-4’	72.80	72.79	0.01
C-5’	71.30	71.24	0.06
C-6’	17.30	17.47	-0.17

Table 4. Comparison of ^{13}C NMR data of the synthetic **4** with those of the natural Prosaikogenin G

Position	^{13}C		
	Reported[S2]	Synthetic **4**	Δδ
1	38.70	38.71	-0.01
2	26.10	26.09	0.01
3	81.80	81.78	0.02
4	43.80	43.77	0.03
5	47.60	47.52	0.08
6	17.70	17.66	0.04
7	31.60	31.62	-0.02
8	42.00	41.95	0.05
9	53.10	53.10	0
10	36.40	36.40	0
11	132.00	132.11	-0.11
12	132.00	132.04	-0.04
13	84.90	84.96	-0.06
14	43.70	43.67	0.03
15	35.50	35.52	-0.02
16	77.20	77.20	0
17	45.40	45.41	-0.01
18	51.40	51.42	-0.02
19	38.50	38.45	0.05
20	32.00	31.97	0.03
21	36.90	36.88	0.02
22	31.30	31.37	-0.07
23	64.50	64.42	0.08
24	13.10	13.12	-0.02
25	18.90	18.94	-0.04
26	19.60	19.63	-0.03
27	18.20	18.18	0.02
28	77.90	77.88	0.02
29	33.80	33.83	-0.03

30	24.50	24.48	0.02
C-1’	106.30	106.39	-0.09
C-2’	73.10	73.05	0.05
C-3’	75.50	75.57	-0.07
C-4’	72.90	72.90	0
C-5’	71.40	71.35	0.05
C-6’	17.50	17.55	-0.05

Table 5. Comparison of ^{13}C NMR data of the synthetic **5** with those of the natural saikosaponin Y

Position	^{13}C		
	Reported[S3]	Synthetic **5**	Δδ
1	39.20	39.18	0.02
2	26.10	26.15	-0.05
3	83.00	82.97	0.03
4	44.10	44.06	0.04
5	48.00	47.98	0.02
6	18.10	18.13	-0.03
7	32.00	32.03	-0.03
8	43.00	42.98	0.02
9	53.80	53.73	0.07
10	37.10	37.07	0.03
11	134.60	134.56	0.04
12	129.60	129.62	-0.02
13	85.60	85.59	0.01
14	50.90	50.85	0.05
15	45.40	45.39	0.01
16	214.80	214.74	0.06
17	57.40	57.36	0.04
18	56.30	56.24	0.06
19	40.00	39.99	0.01
20	32.40	32.45	-0.05
21	36.60	36.64	-0.04
22	25.10	25.09	0.01
23	64.70	64.71	-0.01
24	12.70	12.81	-0.11
25	18.80	18.89	-0.09
26	20.10	20.17	0.03
27	20.70	20.76	-0.06
28	76.30	76.29	0.01
29	33.80	33.84	-0.04
30	23.50	23.53	-0.03
C-1’	105.70	105.65	0.05

C-2’	71.90	71.82	0.08
C-3’	85.20	85.13	0.07
C-4’	72.30	72.33	-0.03
C-5’	71.30	71.32	-0.02
C-6’	16.90	16.99	-0.09
C-1"	105.70	105.65	0.05
C-2"	75.40	75.34	0.06
C-3"	77.70	77.67	0.03
C-4"	71.20	71.19	0.01
C-5"	77.90	77.89	0.01
C-6"	62.40	62.36	0.04

Table 6. Comparison of ^{13}C NMR data of the synthetic **7** with those of the natural clinposaponin I

Position	^{13}C		
	Reported[S4]	Synthetic **7**	Δδ
1	38.70	38.71	-0.01
2	26.10	26.14	-0.04
3	81.80	81.69	0.11
4	43.70	43.77	-0.07
5	47.50	47.39	0.11
6	17.60	17.60	0
7	31.60	31.67	-0.07
8	42.30	42.25	0.05
9	53.20	53.16	0.04
10	36.40	36.32	0.08
11	132.20	132.25	-0.05
12	131.20	131.23	-0.03
13	84.00	84.04	-0.04
14	45.70	45.68	0.02
15	36.20	36.19	0.01
16	64.10	64.05	0.05
17	47.00	47.06	-0.06
18	52.20	52.19	0.01
19	37.80	37.77	0.03
20	31.60	31.63	-0.03
21	34.80	34.72	0.08
22	25.80	25.83	-0.03
23	64.20	64.09	0.11
24	13.00	13.1	-0.1
25	18.80	18.81	-0.01
26	20.10	20.12	-0.02
27	20.90	20.90	0

28	73.10	73.09	0.01
29	33.70	33.70	0
30	23.90	23.88	0.02
C-1’	106.00	106.15	-0.15
C-2’	71.70	71.73	-0.03
C-3’	85.00	84.99	0.01
C-4’	72.10	72.16	-0.06
C-5’	71.10	71.08	0.02
C-6’	17.30	17.39	0.09
C-1''	106.20	106.30	-0.1
C-2''	75.30	75.35	-0.05
C-3''	78.40	78.48	-0.08
C-4''	71.80	71.74	0.06
C-5''	77.40	77.38	0.02
C-6''	70.30	70.24	0.06
C-1'''	105.50	105.55	-0.05
C-2'''	75.50	75.56	-0.06
C-3'''	78.40	78.44	-0.04
C-4'''	71.70	71.54	0.16
C-5'''	78.40	78.43	-0.03
C-6'''	62.80	62.62	0.18

Reference:

S1. Lee, S.-W.; Kim, M.-S.; Lim, J.-H.; Chang, J.-S.; Ling, J.; Bae, K.-H.; Lee, W.-S.; Rho, M.-C., Inhibition of VLA-4/VCAM-1-mediated cell adhesion by triterpenoid saponins from Bupleurum falcatum L. *Bull. Korean Chem. Soc.* **2010**, *31*, 1931-1936.

S2. Shimizu, K.; Amagaya, S.; Ogihara, Y., New derivatives of saikosaponins. *Chem. Pharm. Bull.* **1985**, *33*, 3349-3355.

S3. Liu, X.; Latkolik, S.; Atanasov, A. G.; Kunert, O.; Pferschy-Wenzig, E.-M.; Heiss, E. H.; Malainer, C.; Schinkovitz, A.; Kollroser, M.; Dirsch, V. M.; Bauer, R., *Bupleurum chinense* roots: a bioactivity-guided approach toward saponin-type NF-κB inhibitors. *Planta. Med.* **2017**, *83*, 1242-1250.

S4. Yamamoto, A.; Miyase, T.; Ueno, A.; Maeda, T., Clinoposaponins I-V, new oleanane-triterpene saponins from Clinopodium gracile O. Kuntze. *Chem. Pharm. Bull.* **1993**, *41*, 1270-1274.
